# Well-balanced methods for computational astrophysics

**DOI:** 10.1007/s41115-022-00014-6

**Published:** 2022-10-19

**Authors:** Roger Käppeli

**Affiliations:** Seminar for Applied Mathematics, Eidgenössische Technische Hochschule Zürich, Rämistrasse 101, 8092 Zurich, Switzerland

**Keywords:** Numerical methods, Hydrodynamics, Source terms, Well-balanced schemes

## Abstract

We review well-balanced methods for the faithful approximation of solutions of systems of hyperbolic balance laws that are of interest to computational astrophysics. Well-balanced methods are specialized numerical techniques that guarantee the accurate resolution of non-trivial steady-state solutions, that balance laws prominently feature, and perturbations thereof. We discuss versatile frameworks and techniques for generic systems of balance laws for finite volume and finite difference methods. The principal emphasis of the presentation is on the algorithms and their implementation. Subsequently, we specialize in hydrodynamics’ Euler equations to exemplify the techniques and give an overview of the available well-balanced methods in the literature, including the classic hydrostatic equilibrium and steady adiabatic flows. The performance of the schemes is evaluated on a selection of test problems.

## Introduction

Numerical methods for the approximate solution of balance laws play a central role in the simulation of many interesting and challenging phenomena in computational astrophysics. Balance laws take the generic form

1 $$\begin{aligned} \frac{\partial \varvec{u}}{\partial t} + \nabla \cdot {{{\textbf {{\textsf {f}}}}}} = \varvec{s} , \end{aligned}$$

where $\varvec{u}$ is the vector of conserved variables, ${{{\textbf {{\textsf {f}}}}}}$ the flux tensor and $\varvec{s}$ the vector of source terms, respectively. Examples of balance laws include (ideal) hydrodynamics or Euler equations, (ideal) magnetohydrodynamics, and radiation (magneto)hydrodynamics, as well as their relativistic counterparts. The origin of the source term on the right-hand side may be physical (e.g., chemical reactions, external forces, non-ideal effects), geometric (e.g., curvilinear coordinates) or both (e.g., curved spacetime).

The faithful modeling of complex astrophysical phenomena with balance laws is generally not feasible with (semi-)analytical methods alone. Hence, solutions can only be sought approximately by numerical means. Numerical methods for (hyperbolic) conservation laws, that is, the homogeneous Eq. (1) with $\varvec{s} \equiv 0$, are in a mature stage of development. We refer, for example, to the recent comprehensive review by Balsara (2017) and references therein. The approximation of balance laws is often not much more involved and can easily be done by supplementing a consistent discretization of the source term $\varvec{s}$. In this way, highly accurate solution approximations can be obtained efficiently by computational means.

However, there are particular regimes where conventional numerical methods encounter difficulties.^1^ Balance laws often possess non-trivial steady-state solutions

2 $$\begin{aligned} \nabla \cdot {{{\textbf {{\textsf {f}}}}}} = \varvec{s} , \end{aligned}$$

where the flux divergence exactly balances the source term. Numerical methods do not necessarily satisfy a discrete version of this subtle equilibrium balance. Consequently, steady states are not resolved exactly but are approximated with an error of the order of the method’s truncation error. To simulate phenomena near steady states, the numerical resolution needs to be high enough such that the continuous pile-up of these truncation errors does not obscure the processes of interest during the simulation timeframe. Especially in multi-dimensional simulations, the required resolution may entail prohibitively high computational costs.

The shortcoming near steady states was realized early on in the development of numerical methods for balance laws. This lead to the suggestion to exploit the steady-state solutions in the discrete representation of the approximate solution (see, e.g., Liu 1979; Glaz and Liu 1984; Glimm et al. 1984; van Leer 1984; Huang and Liu 1986; Roe 1987). The idea is to replace the common (piecewise) polynomial approximate solution representation with a (piecewise) steady one that fulfills the subtle balance Eq. (2) either exactly or approximately. Thereby, only deviations from steady state induce dynamics which is indeed highly desirable. As a matter of fact, this is a generalization of the fundamental property of numerical methods for the homogeneous equations that only deviations from a constant state trigger wave motion. As noted by van Leer (1984), the construction of such steady-state distributions is in general difficult as it requires the local solution of a boundary value problem for Eq. (2). The solvability is challenging and a solution can usually only be obtained numerically. The pioneering work by Mellema et al. (1991), Eulderink and Mellema (1995) suggests constructing an equilibrium subgrid model by locally approximating equivalent initial value problems numerically. For example, such equilibrium subgrid models were successfully constructed for hydrostatic equilibrium by Zingale et al. (2002) and even for general relativity by Kastaun (2006). This led to much improved numerical resolution near equilibrium states.

An additional design principle was introduced by Cargo and LeRoux (1994) to overcome the challenges near steady states. They constructed a scheme for the Euler equations with gravity source terms capable of preserving exactly a discrete form of hydrostatic equilibrium and termed the scheme as *well-balanced* (or, in French, “un schéma équilibre”). A well-balanced numerical method satisfies a discrete form of the equilibrium balance Eq. (2) exactly, independent of the resolution. Therefore, these methods can accurately resolve solutions that are small perturbations of equilibrium data. Many such schemes have been developed since, especially for the shallow water equations with bottom topography used in environmental applications. In the context of the shallow water equations, the well-balanced property is also referred to as the *exact C-Property* put forward in a seminal paper by Bermudez and Vazquez (1994). We refer to the comprehensive reviews by Noelle et al. (2009), Xing and Shu (2014), Kurganov (2018), the textbook by Bouchut (2004) and the references therein for further information. An extensive review of well-balanced and related schemes for many applications can also be found in the textbook by Gosse (2013). Moreover, we refer to Amadori and Gosse (2015) for an extensive theoretical treatment and rigorous numerical analysis of well-balanced schemes on simple balance laws.

Well-balanced methods for balance laws commonly used in computational astrophysics have received much attention in the literature recently. Pioneering schemes for the Euler equations have been developed by Cargo and LeRoux (1994), coining the term well-balanced, and LeVeque et al. (1998), LeVeque and Bale (1999). The latter apply the quasi-steady wave-propagation algorithm of LeVeque (1998). Botta et al. (2004) designed a well-balanced finite volume scheme for numerical weather prediction applications. More recently, a multitude of well-balanced numerical schemes have been elaborated for the Euler equations in the literature (LeVeque 2010; Xu et al. 2010; Luo et al. 2011; Xing and Shu 2013; Käppeli and Mishra 2014; Vides et al. 2014; Desveaux et al. 2014; Chandrashekar and Klingenberg 2015; Desveaux et al. 2015; Ghosh and Constantinescu 2015; Li and Xing 2016b; Käppeli and Mishra 2016; Li and Xing 2016a; Touma et al. 2016; Ghosh and Constantinescu 2016; Franck and Mendoza 2016; Bispen et al. 2017; Käppeli 2017; Chandrashekar and Zenk 2017; Berberich et al. 2018; Li and Xing 2018b, a; Chertock et al. 2018; Gaburro et al. 2018; Qian et al. 2018; Grosheintz-Laval and Käppeli 2019; Popov et al. 2019; Thomann et al. 2019; Klingenberg et al. 2019; Veiga et al. 2019; Varma and Chandrashekar 2019; Krause 2019; Thomann et al. 2020; Grosheintz-Laval and Käppeli 2020; Berberich et al. 2019; Padioleau et al. 2019; Kanbar et al. 2020; Castro and Parés 2020; Berberich et al. 2021a, b; Parés and Parés-Pulido 2021; Li and Gao 2021; Wu and Xing 2021; Edelmann et al. 2021; Gómez-Bueno et al. 2021a, b). Magneto-hydrostatic steady-state preserving well-balanced schemes were devised by Fuchs et al. (2010a, 2010b, 2011). Well-balanced schemes for relativistic hydrodynamics on curved spacetime were considered by Kastaun (2006), LeFloch and Makhlof (2014), Gosse (2015), LeFloch et al. (2020), Gaburro et al. (2021).

A popular framework for the construction of well-balanced numerical methods is rooted in the piecewise steady or subgrid equilibrium representation. The framework combines a piecewise steady reconstruction, consisting of an equilibrium subgrid model and a piecewise polynomial equilibrium-preserving reconstruction, and a well-balanced source term discretization. Many of the aforementioned schemes above have been constructed along these ingredients. In this text, we focus on this framework as it combines conceptual simplicity and versatility in that it applies to a wide range of numerical methods for balance laws ranging from finite volume to discontinuous Galerkin over finite difference methods. For the clarity and conciseness of the presentation, we concentrate on particular flavors of these numerical methods. In particular, we focus the presentation on higher-order Godunov-type finite volume methods and finite difference methods with flux splitting. Moreover, the emphasis of this review is on algorithmic ideas, not necessarily on the underlying theory.

Another versatile framework to construct well-balanced methods is based on the reformulation of Eq. (1) as a homogeneous quasi-linear PDE system of the form

3 $$\begin{aligned} \frac{\partial \varvec{u}}{\partial t} + {{{\textbf {{\textsf {A}}}}}}(\varvec{u}) \cdot \nabla \varvec{u} = 0 . \end{aligned}$$

This framework is applied in the context of hyperbolic systems with non-conservative products using the path-conservative finite volume methods (see, e.g., Cargo and LeRoux 1994; Greenberg and LeRoux 1996; Greenberg et al. 1997; Gosse 2000, 2001; Parés and Castro 2004; Parés 2006; Castro et al. 2007, 2008; LeVeque 2010). In this form, a special family of paths in phase space can be constructed such that a well-balanced method results. These paths can be obtained from the explicit knowledge of the solutions of the Riemann problems of Eq. (3), which may be difficult and expensive in general, or through a so-called generalized hydrostatic reconstruction technique by Castro et al. (2007, 2008). The latter is closely related to the piecewie steady reconstruction technique (Castro and Parés 2020). Furthermore, the framework is able to deal with singular source terms. However, we shall not pursue further the presentation of this theoretically pleasing and elegant framework in this text and redirect the interested reader to the given references. See also the recent comprehensive review by Castro et al. (2017) on this framework.

Before we proceed to the outline, we also mention that well-balanced methods may be considered as part of the family of so-called *structure-preserving* methods. These methods are designed such that certain properties of the physical model (i.e., the balance law, the partial differential equation, etc.) are fulfilled in some form at the discrete level. Such properties may be in the form of so-called *companion balance* or *conservation laws* that are automatically satisfied at the analytical level. For example, the second law of thermodynamics puts admissibility criteria in the form of entropy conditions on flow discontinuities such as shock waves. The preservation of physical states, e.g., positive mass density, pressure or subluminal velocities. The rotational invariance of the equations of (magneto-)hydrodynamics implies the conservation of angular momentum. Faraday’s law, together with the fact that magnetic monopoles have never been observed in nature, imply the solenoidal character of the magnetic field in Maxwell’s equations and magnetohydrodynamics. Self-gravitating flows conserve total momentum and energy. Although consistent numerical methods may fulfill such structures in the infinite resolution limit, this is often unsatisfactory in practice as the needed resolution may result in unaffordable large computational costs. Also, note that near discontinuities, which solutions of balance laws prominently feature, these errors are inevitably of order one. Hence, structure-preserving methods are often not a luxury choice but a necessity. The design of structure-preserving methods that maintain a discrete form of such structures is a rich and challenging line of research on numerical methods for balance/conservation laws. We refer the interested reader to, e.g., Evans and Hawley (1988), Balsara and Spicer (1999), Tóth (2000), Morton and Roe (2001), Tadmor (2003), Mishra and Tadmor (2011), Jiang et al. (2013), Després and Labourasse (2015), Schaal et al. (2015), Katz et al. (2016), Zanotti and Dumbser (2016), Balsara and Kim (2016), Wu and Tang (2017, 2018), Mullen et al. (2021) and references therein.

The text is organized as follows:

- Section 2 presents a brief introduction to finite volume methods and motivates the well-balanced methods on the basis of an extremely simple model equation, namely the linear advection–reaction equation. This is followed by a general framework for the construction of well-balanced finite volume methods. The procedure is examplified on the Euler equations in spherical symmetry featuring a geometric source term. The section rounds up with a general discussion of well-balanced methods within finite difference frameworks.
- Section 3 focuses on well-balanced methods for the Euler equations. Several flavors of well-balanced methods are presented with differing local steady-state determination strategies. The section completes by a battery of numerical test problems on which the performance of well-balanced methods is commonly assessed.

Before we proceed, let us state that any inadvertent omission or understatement of credit to authors to whom more was due, we humbly offer a sincere apology in advance.

## Well-balanced discretization

### One-dimensional methods

We begin by considering a one-dimensional system of balance laws in the form

4 $$\begin{aligned} \frac{\partial \varvec{u}}{\partial t} + \frac{\partial \varvec{f}}{\partial x} = \varvec{s} . \end{aligned}$$

Here $\varvec{u}$, $\varvec{f}$ and $\varvec{s}$ are vectors of *m* components: $\varvec{u} = \varvec{u}(x,t)$ is the vector of conserved variables, $\varvec{f} = \varvec{f}(\varvec{u})$ the vector of flux functions, and $\varvec{s} = \varvec{s}(\varvec{u})$ the vector of source terms.^2^ In the following, we will tacitly assume that

(i) the system is of hyperbolic nature: the Jacobian of the flux function vector $A(\varvec{u}) = \frac{\partial \varvec{f}}{\partial \varvec{u}}$ has real eigenvalues and an associated set of linearly independent eigenvectors for all $\varvec{u}$ of interest,
(ii) the source term $\varvec{s}(\varvec{u})$ is not singular: bounded source terms do not change the Rankine–Hugoniot jump conditions of the system.

Next, we outline a standard finite volume discretization of the balance law Eq. (4) in order to introduce our notation and set the stage for the following developments. For further details and precise derivation, we refer to the many excellent textbooks available in the literature, see, e.g., LeVeque (1992, 2002), Godlewski and Raviart (1996), Laney (1998), Hirsch (2007), Toro (2009).

### Finite volume discretization

A standard finite volume method discretizes the spatial domain of interest $\varOmega = [0,L]$ into a finite number *N* of control volumes or cells $\varOmega _{i}=[x_{i-1/2},x_{i+1/2}]$ ($i = 1, \ldots ,N$). For the *i*-th cell, $x_{i\pm 1/2}$ denote the left/right cell interfaces and $x_{i} = (x_{i-1/2}+x_{i+1/2})/2$ the cell centers. For ease of presentation, we shall assume a uniform discretization with constant cell size $\varDelta x=x_{i+1/2}-x_{i-1/2}$. However, this assumption can easily be relaxed within a finite volume discretization.

Integrating the balance law Eq. (4) over cell $\varOmega _{i}$ and dividing by the cell size $\varDelta x$ yields

5 $$\begin{aligned} \frac{{\mathrm {d}}\overline{\varvec{u}}_{i}}{{\mathrm {d}}t} + \frac{1}{\varDelta x} \left( \varvec{f}(\varvec{u}(x_{i+1/2},t)) - \varvec{f}(\varvec{u}(x_{i-1/2},t)) \right) = \overline{\varvec{s}}_{i}(t) , \end{aligned}$$

where we introduced

6 $$\begin{aligned} \begin{aligned} \overline{\varvec{u}}_{i}(t)&= \frac{1}{\varDelta x} \int _{\varOmega _{i}} \varvec{u}(x,t) ~ {\mathrm {d}}x , \\ \overline{\varvec{s}}_{i}(t)&= \frac{1}{\varDelta x} \int _{\varOmega _{i}} \varvec{s}(\varvec{u}(x,t)) ~ {\mathrm {d}}x , \end{aligned} \end{aligned}$$

the cell averages of conserved variables and source terms. We also introduce the convention that a quantity with an overbar denotes a cell average, while one without a point value.

Equation (5) represents an exact evolution equation for the cell-averaged conserved variables. The numerical approximation is introduced by replacing the exact fluxes and source terms by so-called numerical fluxes and source terms:

7 $$\begin{aligned} \frac{{\mathrm {d}}\overline{\varvec{U}}_{i}}{{\mathrm {d}}t} = {\mathcal {L}}(\overline{\varvec{U}})_{i} = - \frac{1}{\varDelta x} \left( \varvec{F}_{i+1/2} - \varvec{F}_{i-1/2} \right) + \overline{\varvec{S}}_{i} . \end{aligned}$$

Here, the $\overline{\varvec{U}}_{i}$, $\varvec{F}_{i\pm 1/2}$ and $\overline{\varvec{S}}_{i}$ denote approximations of the cell-averaged conserved variables, the fluxes through the cell interfaces and the cell-averaged source terms at time *t*:

8 $$\begin{aligned} \overline{\varvec{U}}_{i}(t) \approx \overline{\varvec{u}}_{i}(t) , \quad \varvec{F}_{i\pm 1/2}(t) \approx \varvec{f}(\varvec{u}(x_{i\pm 1/2},t)) \quad {\text {and}} \quad \overline{\varvec{S}}_{i}(t) \approx \overline{\varvec{s}}_{i}(t) . \end{aligned}$$

In the following, we use the convention that exact solutions are denoted by lower case letters and approximations by upper case letters.

Equation (7) is a generic semi-discrete^3^ finite volume discretization in one space dimension. Furthermore, in Eq. (7) we denote the so-called spatial discretization operator by ${\mathcal {L}}(\overline{\varvec{U}})_{i}$. Next, we briefly describe the individual components of a finite volume method. For ease of notation, we suppress the temporal dependency.

#### Reconstruction $\mathcal {R}$

The primary unknowns in a finite volume method are the cell averages. To evaluate the numerical fluxes through cell interfaces and compute cell averages of the source terms, within each cell a subcell profile of the conserved variables $\varvec{U}_{i}(x)$ has to be reconstructed from the cell averages $\{\overline{\varvec{U}}_{k}\}$. Because discontinuities may be present in the solution, special care is needed to reconstruct non-oscillatory subcell profiles that avoid spurious Gibbs phenomena.

We denote such a reconstruction procedure $\mathcal {R}$, which recovers an *r*-th order accurate profile $Q_{i}(x)$ of a quantity *q*(*x*) within cell $\varOmega _{i}$ from the cell averages $\{\overline{q}_{k}\}$, by

9 $$\begin{aligned} Q_{i}(x) = \mathcal {R}(x; \{\overline{q}_{k}\}_{k \in S_{i}}) = q(x) + \mathcal {O}(\varDelta x^{r}) \end{aligned}$$

with

10 $$\begin{aligned} \overline{q}_k = \frac{1}{\varDelta x} \int _{\varOmega _{k}} Q_{i}(x) {\mathrm {d}}x \quad {\text {for}} \; k \in S_{i} . \end{aligned}$$

Here $S_{i} = \{\ldots ,i-1,i,i+1,\ldots \}$ is the so-called stencil of the reconstruction for cell $\varOmega _{i}$, which consists of cell $\varOmega _{i}$ and a certain number of neighboring cells. For systems, the reconstruction procedure can be applied component-wise to the cell averages of the conserved variables vector

11 $$\begin{aligned} \varvec{U}_{i}(x) = \mathcal {R}(x; \{\overline{\varvec{U}}_{k}\}_{k\in S_{i}}) . \end{aligned}$$

Numerous such reconstruction procedures have been developed in the literature, and a non-exhaustive list includes the Total Variation Diminishing (TVD) and the Monotonic Upwind Scheme for Conservation Laws (MUSCL) methods (see, e.g., van Leer 1979; Harten et al. 1983; Sweby 1984; Laney 1998; LeVeque 2002; Toro 2009), the Piecewise Parabolic Method (PPM) by Colella and Woodward (1984), the Essentially Non-Oscillatory (ENO) (see, e.g., Harten et al. 1987), Weighted ENO (WENO) (see, e.g., Shu 2009 and references therein) and Central WENO (CWENO) methods (see, e.g., Levy et al. 1999, 2000; Cravero et al. 2018).

For example, a spatially first-order accurate reconstruction consists of a piecewise constant profile

12 $$\begin{aligned} \varvec{U}_{i}(x) = \mathcal {R}(x; \{\overline{\varvec{U}}_{i}\}) = \overline{\varvec{U}}_{i} . \end{aligned}$$

A spatially second-order accurate piecewise linear reconstruction à la TVD/MUSCL is given by

13 $$\begin{aligned} \varvec{U}_{i}(x) = \mathcal {R}(x; \{ \overline{\varvec{U}}_{i-1}, \overline{\varvec{U}}_{i} , \overline{\varvec{U}}_{i+1} \}) = \overline{\varvec{U}}_{i} + D\varvec{U}_{i} ~ (x - x_{i}) , \end{aligned}$$

where $D\varvec{U}_{i}$ are some appropriately limited slopes (to avoid monotonicity violation and ensuing spurious oscillations). A popular example is the so-called generalized $\mathrm {minmod}$ slope limiter family

14 $$\begin{aligned} D\varvec{U}_{i} = \mathrm {minmod} \left( \theta \frac{\overline{\varvec{U}}_{i } - \overline{\varvec{U}}_{i-1}}{ \varDelta x} , \frac{\overline{\varvec{U}}_{i+1} - \overline{\varvec{U}}_{i-1}}{2 \varDelta x} , \theta \frac{\overline{\varvec{U}}_{i+1} - \overline{\varvec{U}}_{i }}{ \varDelta x} \right) , \end{aligned}$$

where $\theta \in [1,2]$ is a parameter and the $\mathrm {minmod}$ function is defined by

15 $$\begin{aligned} \mathrm {minmod}(a_1,a_2,...) = {\left\{ \begin{array}{ll} \min _j \left\{ a_j \right\} &{} {\mathrm {if}} \; a_j > 0 \; \forall \; j , \\ \max _j \left\{ a_j \right\} &{} {\mathrm {if}} \; a_j < 0 \; \forall \; j , \\ 0 &{} {\mathrm {otherwise}} . \end{array}\right. } \end{aligned}$$

**Fig. 1:**
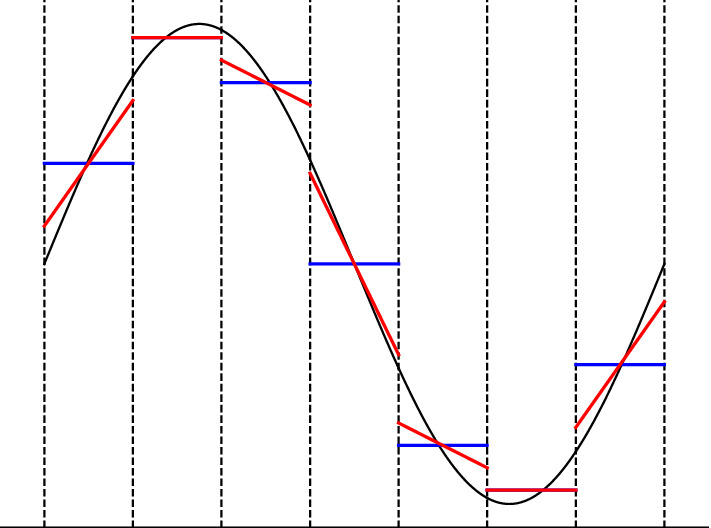
Illustration of the reconstruction procedure for the sine function $u(x) = \sin (x)$ (solid black line). Within each control volume or cell is shown the cell average $\overline{U}_{i}$ (solid blue) and a piecewise linear TVD/MUSCL reconstruction $U_{i}(x)$ (solid red). Notice the limiter’s action near the two extrema, where the slopes are clipped to ensure the monotonicity of the reconstruction. The piecewise constant cell averages correspond to a piecewise constant reconstruction

Equation (14) has to be understood component-wise. For $\theta = 1$ ($\theta = 2$), Eq. (14) reproduces the traditional $\mathrm {minmod}$ (monotonized centered) limiter (see, e.g., Toro 2009 and references therein for further information). See Fig. 1 for an illustration of a piecewise constant/linear reconstruction.

Straightforward component-wise reconstruction for systems of balance laws may sometimes lead to some undesirable oscillations in the results, especially when strong flow discontinuities interact. In that case, it may prove beneficial to perform the reconstruction in *local characteristic variables* (see, e.g., Harten et al. 1987; Qiu and Shu 2002; Toro 2009)

16 $$\begin{aligned} \varvec{U}_{i}(x) = R_{i} ~ \mathcal {R}(x; \{L_{i} \overline{\varvec{U}}_{k}\}_{k\in S_{i}}) , \end{aligned}$$

where $L_{i} = L(\overline{\varvec{U}}_{i})$ and $R_{i} = R(\overline{\varvec{U}}_{i})$ are the matrices of left and right eigenvectors, respectively. The eigenvectors are typically evaluated at the cell average of the *i*-th cell $\varOmega _{i}$ whose reconstruction is performed, hence the name local.

#### Numerical fluxes $\mathcal {F}$

The numerical fluxes are obtained by resolving the discontinuities at cell interfaces naturally arising from the per cell reconstruction (see Fig. 1). This is commonly done à la Godunov by solving (approximately) the Riemann problem at cell interfaces

17 $$\begin{aligned} \varvec{F}_{i+1/2} = \mathcal {F}(\varvec{U}_{i+1/2-} , \varvec{U}_{i+1/2+}) = \mathcal {F}(\varvec{U}_{L} , \varvec{U}_{R}) , \end{aligned}$$

where the point values $\varvec{U}_{i+1/2\pm }$ are the cell interface reconstructed conserved variables

18 $$\begin{aligned} \varvec{U}_{i+1/2-} = \varvec{U}_{L} = \varvec{U}_{i }(x_{i+1/2}) \quad {\text {and}} \quad \varvec{U}_{i+1/2+} = \varvec{U}_{R} = \varvec{U}_{i+1}(x_{i+1/2}) . \end{aligned}$$

Notice that the value on the left (L)/right (R) of the interface $x_{i+1/2}$ is obtained from the reconstruction in cell $\varOmega _{i}$/$\varOmega _{i+1}$. The numerical flux is required to be *consistent* with the physical flux function $\varvec{f}$, i.e., $\mathcal {F}(\varvec{U},\varvec{U}) = \varvec{f}(\varvec{U})$, and *Lipschitz continuous*. The latter is required for accuracy reasons (see, e.g., Harten et al. 1987). Moreover, certain numerical fluxes have the ability to exactly recognize isolated discontinuities such as contacts or shocks in (magneto-) hydrodynamics (see, e.g., Toro 2009 for details).

A simple and popular choice for the numerical flux is the so-called Rusanov flux (Rusanov 1962; Toro 2009)

19 $$\begin{aligned} \mathcal {F}(\varvec{U}_{L} , \varvec{U}_{R}) = \frac{1}{2} (\varvec{F}_{L} + \varvec{F}_{R}) - \frac{S_{\text {max}}}{2} (\varvec{U}_{R} - \varvec{U}_{L}) , \end{aligned}$$

where $\varvec{F}_{L/R} = \varvec{f}(\varvec{U}_{L/R})$ and $S_{\text {max}}$ is an estimate of the largest characteristic speed in the solution of the Riemann problem $S_{\text {max}} = \max _{m} |\lambda _{m}|$ ($\lambda _{m}$ are the eigenvalues of the flux Jacobian). This numerical flux is sometimes also called a local Lax–Friedrichs (LLF) flux.

#### Numerical source terms $\mathcal {S}$

For the integration of the source terms, there are essentially two standard methods. The first one is the so-called *unsplit method*. In this method, the source terms are typically incorporated directly into the spatial discretization operator as tacitly already done in Eq. (7). An accurate approximation of the cell average of the source terms are obtained by numerical integration. Let ${\mathcal {Q}}_{i}$ denote a *q*-th order accurate quadrature rule over the *i*-th cell $\varOmega _{i}$. The cell average of the source terms are then computed by

20 $$\begin{aligned} \overline{\varvec{S}}_{i}(t) = \frac{1}{\varDelta x} {\mathcal {Q}}_{i} \left( \varvec{s}(\varvec{U}_{i}) \right) = \frac{1}{\varDelta x} \sum _{\alpha =1}^{N_{q}} \omega _{\alpha } ~ \varvec{s} \left( \varvec{U}_{i}(x_{i,\alpha }) \right) , \end{aligned}$$

where the $x_{i,\alpha } \in \varOmega _{i}$ and $\omega _{\alpha }$ denote the $N_{q}$ quadrature nodes and weights of ${\mathcal {Q}}_{i}$, respectively. Assuming that the point values of the source terms can be evaluated with spatial order of accuracy *s*, then the resulting discretization is spatially $\min (q,s)$-th order accurate (provided enough smoothness, of course).^4^ A popular example is the second-order accurate midpoint rule

21 $$\begin{aligned} \overline{\varvec{S}}_{i}(t) = \frac{1}{\varDelta x} {\mathcal {Q}}_{i}(\varvec{s}(\varvec{U}_{i})) = \varvec{s} \left( \varvec{U}_{i}(x_{i}) \right) . \end{aligned}$$

Higher-order rules are provided, for example, by the Gauss-Legendre or Gauss-Lobatto quadrature rules (see, e.g., Press et al. 1993).

The second family of methods are the so-called *splitting* or *fractional-step methods*. In these methods, the original problem Eq. (4) is first split (or fractured) into two subproblems of the form:

22a $$\begin{aligned} {\text {Problem A:}}&\quad \frac{\partial \varvec{u}}{\partial t} + \frac{\partial \varvec{f}}{\partial x} = 0 , \end{aligned}$$

22b $$\begin{aligned} {\text {Problem B:}}&\quad \frac{{\mathrm {d}}\varvec{u}}{{\mathrm {d}}t} = \varvec{s}. \end{aligned}$$

In this approach, one alternates adroitly between solving the two subproblems A and B. This is indeed a very practical approach: Problem A is a standard (homogeneous) conservation law and Problem B is a simple Ordinary Differential Equation (ODE). For both subproblems, there exist many excellent numerical methods and software libraries which implement them. By extension, this approach allows for straightforward modularization. Next, we catalogue two popular splitting methods.

Let $S_{A}^{\varDelta t}$ and $S_{B}^{\varDelta t}$ denote the discrete solution operators that advance a discrete solution $\overline{\varvec{U}}^{n}$ by a time step $\varDelta t$ for problems *A* and *B*

23a $$\begin{aligned} \overline{\varvec{U}}^{n+1*}&= S_{A}^{\varDelta t} \overline{\varvec{U}}^{n}, \end{aligned}$$

23b $$\begin{aligned} \overline{\varvec{U}}^{n+1*}&= S_{B}^{\varDelta t} \overline{\varvec{U}}^{n} , \end{aligned}$$

where the superscript labels the time step and the “star” shall stress the fact that these are only partially evolved states.

An obvious splitting method is then given by

24 $$\begin{aligned} \overline{\varvec{U}}^{n+1} = S_{B}^{\varDelta t} S_{A}^{\varDelta t} \overline{\varvec{U}}^{n} , \end{aligned}$$

which is first-order accurate in time (provided that $S_{A}^{\varDelta t}$ and $S_{B}^{\varDelta t}$ are at least of that same temporal order). This splitting is sometimes termed as *Godunov splitting*. A second-order accurate in time method is given by the so-called *Strang splitting*

25 $$\begin{aligned} \overline{\varvec{U}}^{n+1} = S_{B}^{\varDelta t/2} S_{A}^{\varDelta t} S_{B}^{\varDelta t/2} \overline{\varvec{U}}^{n} , \end{aligned}$$

where one sandwiches a full step $\varDelta t$ with solution operator *A* between two half steps $\varDelta t/2$ with solution operator *B*. Of course, full second-order accuracy in time requires that the individual solution operators $S_{A}^{\varDelta t}$ and $S_{B}^{\varDelta t}$ possess an equivalent or higher order of accuracy.

#### Time discretization $\mathcal {T}$

The semi-discrete evolution equations Eq. (7) for the cell averages $\overline{\varvec{U}}_{i}$ represent a system of ordinary differential equations that has to be approximately integrated in time. For that purpose, the temporal domain of interest $T = [t_{i}, t_{f}]$ is discretized into time steps $\varDelta t^{n} = t^{n+1} - t^{n}$, where the superscripts label the respective time step.

The simplest time integration method is of course the temporally first-order accurate explicit Euler method

26 $$\begin{aligned} \overline{\varvec{U}}^{n+1} = \overline{\varvec{U}}^{n} + \varDelta t^n {\mathcal {L}}(\overline{\varvec{U}}^{n}) . \end{aligned}$$

For higher-order time integration, there are essentially two large families of methods: Runge-Kutta and predictor-corrector methods. A popular representative of the Runge-Kutta family is the temporally second-order accurate explicit Heun method

27 $$\begin{aligned} \begin{aligned} \overline{\varvec{U}}^{(1)}&= \overline{\varvec{U}}^{n } + \varDelta t^n {\mathcal {L}}(\overline{\varvec{U}}^{n }), \\ \overline{\varvec{U}}^{n+1}&= \frac{1}{2} \overline{\varvec{U}}^{n } + \frac{1}{2} \left( \overline{\varvec{U}}^{(1)} + \varDelta t^n {\mathcal {L}}(\overline{\varvec{U}}^{(1)}) \right) . \end{aligned} \end{aligned}$$

It is a so-called Strong Stability-Preserving Runge–Kutta (SSP-RK) method, and it is often labeled by SSP-RK2 in the literature (because it is a two-stage SSP-RK method). These methods have certain desirable stability properties when integrating non-linear conservation/balance laws (see, e.g., Gottlieb et al. 2001 and references therein). Another very popular method is the third-order accurate SSP-RK3 method, which is shown below in Eq. (126).

A popular representative of the predictor-corrector methods is the temporally second-order accurate MUSCL-Hancock method (van Leer 1984). Possible high-order extensions of this methodology can be achieved by evolution of the solution in the small with help of the Cauchy–Kowalevski procedure (Harten et al. 1987). Another possibility is provided by the so-called Arbitrary DERivative (ADER) methods (see, e.g., Castro and Toro 2008; Toro 2009 and references therein). A distinctive feature of predictor-corrector methods is that they are one-step methods, which makes them extremely attractive in an adaptive mesh refinement context (see, e.g., Balsara 2017).

For time explicit approaches as above, Eqs. (26) and (27), the time step $\varDelta t^{n}$ is in general required to fulfill a so-called CFL condition of the form (Courant et al. 1928)

28 $$\begin{aligned} \varDelta t^{n} = C_{\mathrm {CFL}} \times \min _{i} \left( \frac{\varDelta x}{\left| S^{n}_{i} \right| } \right) , \end{aligned}$$

where $S^{n}_{i}$ is the speed of the fastest wave in cell $\varOmega _{i}$ at time $t^{n}$ and $C_{\mathrm {CFL}}$ is the CFL number. The latter needs to fall within a certain range for linear stability.

Time implicit approaches are also possible and especially adapted for so-called stiff problems involving vastly different timescales (see, e.g., Kwatra et al. 2009; Viallet et al. 2011, 2013; Kifonidis and Müller 2012; Miczek et al. 2015 and references therein). Stiffness may originate in the different wave propagation characteristics (such as advective versus acoustic waves), strong chemical reactions and many more.

#### Assembling a finite volume scheme

A generic finite volume scheme Eq. (7) for the one-dimensional balance law Eq. (4) is now easily assembled with the previously described components:

1. A spatially *r*-th order accurate reconstruction $\mathcal {R}$ (Eq. (11)).
2. A consistent and Lipschitz continuous numerical flux function $\mathcal {F}$ (Eq. (17)).
3. An unsplit source terms discretization $\mathcal {S}$ (Eq. (20)) based on *s*-th order accurate point value evaluations and a *q*-th order accurate quadrature rule ${\mathcal {Q}}$.
4. A $\tau$-th order accurate time integrator $\mathcal {T}$.

This results in a $\min (q,r,s,\tau )$-th order accurate finite volume scheme (for smooth enough solutions, of course). A similar assemblage can be realized with an appropriate splitting method for the source terms.

However, the approximation of near steady states characterized by the near balance of flux divergence and source term

$$\begin{aligned} \frac{\partial \varvec{u}}{\partial t} = - \frac{\partial \varvec{f}}{\partial x} + \varvec{s} \approx 0 \end{aligned}$$

is quite challenging for such a generic finite volume scheme

$$\begin{aligned} \frac{{\mathrm {d}}\overline{\varvec{U}}_{i}}{{\mathrm {d}}t} = - \frac{1}{\varDelta x} \left( \varvec{F}_{i+1/2} - \varvec{F}_{i-1/2} \right) + \overline{\varvec{S}}_{i} \approx 0 \end{aligned}$$

It turns out that the steady states of interest are not exactly representable by polynomials used in the reconstruction procedure in general. Therefore the piecewise polynomial reconstruction will introduce truncation errors at every time step. Another issue is that the flux divergence and source term discretizations are commonly computed independently. This further makes the above discrete near balance unlikely.

In the next section, we present a simple illustrating example followed by a general technique to *well-balance* such steady states within a finite volume framework.

### Example: linear advection–reaction equation

We now illustrate the issues that can arise when numerically approximating balance laws near steady states. Consider the simple linear advection–reaction equation

29 $$\begin{aligned} \frac{\partial u}{\partial t} + a \frac{\partial u}{\partial x} = - \lambda u \end{aligned}$$

modeling the transport of some radioactive material of concentration *u*(*x*, *t*) with constant advection velocity $a > 0$ and decay rate $\lambda > 0$.

An exact solution is easily derived with the method of characteristics

30 $$\begin{aligned} u(x,t) = e^{-\lambda t} u_{0}(x - a t) , \end{aligned}$$

where $u_{0}(x)$ is the initial concentration $u(x,0) = u_{0}(x)$. The exact solution reflects the anticipated behavior that the initial concentration is advected to the right with velocity *a* and decays along the way with rate $\lambda$.

An interesting feature of the above simple model Eq. (29) is that it possesses non-trivial steady-state solutions

31 $$\begin{aligned} a \frac{\partial u}{\partial x} = - \lambda u , \end{aligned}$$

which are of the form

32 $$\begin{aligned} u(x,t) = C e^{-\lambda /a ~ x} \end{aligned}$$

for some constant *C*. The steady states are characterized by a subtle balance between the advection and decay processes. Their spatial variation is ruled by the ratio between the decay and the advection timescale, commonly known as the Damköhler number $\mathrm {Da} = \frac{\lambda }{a}$.

Let us solve approximately Eq. (29) over the computational domain $\varOmega = [0,2]$ discretized by *N* uniform cells $\varOmega _{i}$ ($i=1,\dots ,N$). For illustration, we choose two first-order accurate finite volume schemes. The first scheme consists of piecewise constant reconstruction Eq. (12), the Rusanov numerical flux Eq. (19), the unsplit source term discretization based on the midpoint rule Eq. (21), and the explicit Euler time integration Eq. (26). Explicitly, this gives the following fully discrete evolution equation for the cell-averaged concentration $\overline{U}_{i}^{n}$ within cell $\varOmega _{i}$:

33 $$\begin{aligned} \overline{U}_{i}^{n+1} = \overline{U}_{i}^{n} - \frac{\varDelta t}{\varDelta x} a \left( \overline{U}_{i}^{n} - \overline{U}_{i-1}^{n} \right) - \varDelta t \lambda \overline{U}_{i}^{n} . \end{aligned}$$

The second scheme employs Godunov splitting Eq. (24) for the source term discretization and reads:

34a $$\begin{aligned} \overline{U}_{i}^{*}&= \overline{U}_{i}^{n} - \frac{\varDelta t}{\varDelta x} a \left( \overline{U}_{i }^{n} - \overline{U}_{i-1}^{n} \right) \end{aligned}$$

34b $$\begin{aligned} \overline{U}_{i}^{n+1}&= \overline{U}_{i}^{*} - \varDelta t \lambda \overline{U}_{i}^{*} . \end{aligned}$$

Note that explicit Euler time integration is used in both subproblems. In principle, the exact solution could be used in Eq. (34b), i.e., $\overline{U}_{i}^{n+1} = e^{- \lambda \varDelta t} \overline{U}_{i}^{*}$. However, this does not affect the following discussion. Without any surprise, an astute reader will recognize here the classical first-order upwind method for the linear advection equation. Both schemes are first-order accurate in space and time and are linearly stable provided that the time step $\varDelta t$ is chosen such that $0 < \frac{\varDelta t}{\varDelta x} a \le 1$ and $0< \varDelta t \lambda < 2$.

**Fig. 2:**
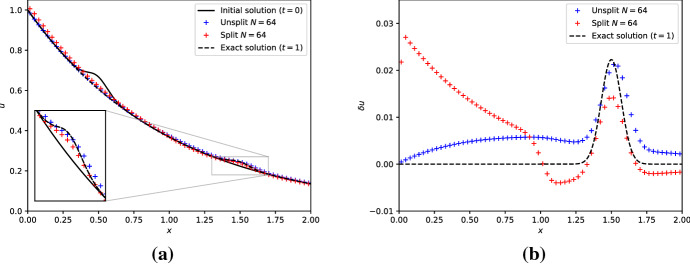
The left panel shows the slightly perturbed steady state initial concentration Eq. (32) (solid black line) and the exact solution at the time final $t_{f} = 1$ (dashed black line). The same panel also shows the results obtained with the unsplit/split first-order schemes with a resolution of $N = 64$ cells. The right panel shows the equilibrium perturbation (solution minus the steady-state background) at the final time

We fix $a = \lambda = 1$ and evolve a slightly perturbed steady state Eq. (32) as shown in Fig. 2a for one time unit. The small perturbation centered around $x = 0.5$ is advected by one unit to the right and its amplitude decays by a factor $e^{-1}$. In the same panel are also shown the approximate results obtained with the unsplit Eq. (33) and split Eq. (34) first-order schemes. Both schemes show qualitatively correct results. More quantitatively, Fig. 2b displays the equilibrium perturbation, that is, the difference between the solution and the background steady state. We observe that the perturbation is indeed advected by the correct distance by both schemes. However, we also observe that both schemes show significant discrepancies with the expected solution away from the perturbation.

**Fig. 3:**
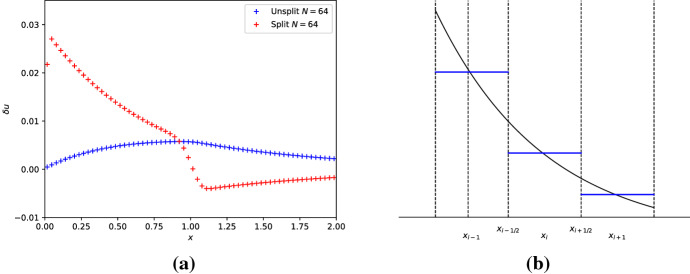
The left panel shows the equilibrium perturbation for the numerically evolved steady state with a resolution of $N=64$ cells at final time $t_{f} = 1$. The blue and red pluses are obtained with the unsplit/split first-order schemes. The right panel shows the steady-state profile (solid black line) together with the cell averages $\overline{U}_{i}^{0}$ (blue solid lines) at initial time for a few cells. The latter also corresponds to the piecewise constant reconstruction on which the unsplit/split first-order schemes are based

To further highlight the issue, we evolve the unperturbed steady state Eq. (32) for one time unit with both schemes. The results are shown in Fig. 3a. By comparison with Fig. 2b, we see clear evidence that the spurious deviations are due to the inability of both schemes to maintain the steady state discretely. To illustrate the origin of the problem, Fig. 3b shows the exact steady state together with the cell averages $\overline{U}_{i}^{0}$ at the initial time for a few cells. The cell averages also correspond to the piecewise constant solution representation within each cell resulting from the first-order reconstruction. It is clear that these piecewise constant subcell profiles are inadequate to represent the steady state within the cells faithfully. More precisely, the piecewise constant approximation of the exponentially varying steady state Eq. (32) inevitably introduces truncation errors of order $\mathcal {O}(\varDelta x)$.

Likewise, higher-order polynomial reconstruction procedures of order *r* introduce truncation errors of order $\mathcal {O}(\varDelta x^{r})$. Therefore, the schemes will introduce local truncation errors of order $\mathcal {O}(\varDelta x^{r})$ near non-polynomial steady states. If the goal is to simulate small perturbations on top of a steady state, the numerical resolution needs to be increased to the point that these local truncation errors do not obscure the phenomena of interest. Similarly, if the goal is to simulate phenomena near a steady state for an extended time (compared to a characteristic timescale on which the steady state would react to equilibrium perturbations), the resolution needs to be increased such that the pile-up of these local truncation errors in each time step does not corrupt the phenomena of interest. This increase in resolution may cause prohibitively high computational costs, especially in multiple dimensions.

This inadequacy of standard piecewise reconstruction procedures was realized early on in the development of numerical methods for balance laws. This motivated for example Liu (1979), Glaz and Liu (1984), and van Leer (1984) to replace the piecewise constant reconstruction within each cell

35 $$\begin{aligned} U_{i}^{n}(x) = \overline{U}_{i}^{n} , \quad x \in \varOmega _{i} , \end{aligned}$$

by a *piecewise steady reconstruction*

36 $$\begin{aligned} U_{i}^{n}(x) = U_{eq,i}^{n}(x) , \quad x \in \varOmega _{i} , \end{aligned}$$

which fulfills the steady state Eq. (31)

37 $$\begin{aligned} a \frac{\partial }{\partial x} U_{eq,i}^{n}(x) = - \lambda U_{eq,i}^{n}(x) \end{aligned}$$

and matches with the *i*-th cell’s average

38 $$\begin{aligned} \frac{1}{\varDelta x} \int _{\varOmega _{i}} U_{eq,i}^{n}(x) ~ {\mathrm {d}}x = \overline{U}_{i}^{n} . \end{aligned}$$

Note that this equilibrium subcell profile $U_{eq,i}^{n}(x)$ depends on the cell under consideration and may be adapted in each time step. Hence the subscripts “*eq*” and “*i*”, and the superscript “*n*”. Since the steady states of the considered linear advection–reaction equation are known explicitly Eq. (32), the desired piecewise steady reconstruction is of the form

39 $$\begin{aligned} U_{eq,i}^{n}(x) = C_{i} ~ e^{- \lambda / a (x - x_{i})}, \quad x \in \varOmega _{i} , \end{aligned}$$

and the constant $C_{i}$ is simply fixed by matching with the *i*-th cell’s average Eq. (38)

40 $$\begin{aligned} C_{i} = \frac{\varDelta x}{2} \frac{\lambda }{a} \frac{\overline{U}_{i}^{n}}{\sinh \left( \frac{\lambda }{a} \frac{\varDelta x}{2}\right) } . \end{aligned}$$

Plugging this reconstruction into the unsplit first-order scheme gives

41 $$\begin{aligned} \overline{U}_{i}^{n+1} = \overline{U}_{i}^{n} - \frac{\varDelta t}{\varDelta x} a \left( U_{eq,i }^{n}(x_{i+1/2}) - U_{eq,i-1}^{n}(x_{i-1/2}) \right) - \varDelta t \lambda \overline{U}_{i}^{n} . \end{aligned}$$

Analogously for the split first-order scheme, one obtains

42a $$\begin{aligned} \overline{U}_{i}^{*}&= \overline{U}_{i}^{n} - \frac{\varDelta t}{\varDelta x} a \left( U_{eq,i }^{n}(x_{i+1/2}) - U_{eq,i-1}^{n}(x_{i-1/2}) \right) \end{aligned}$$

42b $$\begin{aligned} \overline{U}_{i}^{n+1}&= \overline{U}_{i}^{*} - \varDelta t \lambda \overline{U}_{i}^{*} . \end{aligned}$$

**Fig. 4:**
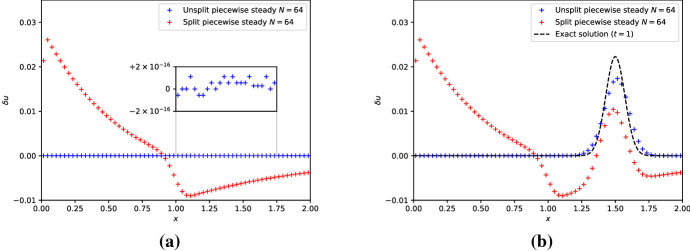
The left panel shows the equilibrium perturbation for the numerically evolved steady state with a resolution of $N=64$ cells at final time $t_{f} = 1$. The blue and red pluses are obtained with the unsplit/split first-order schemes using the piecewise steady reconstruction. The zoom in shows that the unsplit scheme is able to preserve the steady state down to machine precision. The right panel shows the equilibrium perturbation for the slightly perturbed steady-state concentration simulated with the unsplit/split first-order schemes using the piecewise steady reconstruction. The unsplit scheme clearly relaxes back to the steady state away from the concentration perturbation

Let’s evolve the unperturbed steady state with the split and unsplit schemes using the above piecewise steady reconstruction Eq. (36). The resulting equilibrium perturbation at final time $t_{f} = 1$ is shown in Fig. 4a. By comparison with Fig. 3a, we observe that the piecewise steady reconstruction does not improve the situation for the split scheme. Actually, the spurious equilibrium deviations are even slightly worse in this example. In contrast, the unsplit scheme with piecewise steady reconstruction preserves the steady state down to machine precision ($\approx 10^{-16}$ for the double precision floating-point representation used in the computations). Figure 4b displays the results for the slightly perturbed steady state. The split scheme evolves the perturbation faithfully, but is afflicted by the scheme’s local truncation errors at the steady state. On the other hand, the unsplit scheme not only advect the bump very well; it additionally relaxes back to the steady state once the perturbation passed through.

A straightforward computation shows that the unsplit first-order scheme with piecewise steady reconstruction is *exact* for the advection–reaction equation’s steady states Eq. (32). Cargo and LeRoux (1994) subsequently coined the term *well-balanced* for numerical schemes with the property of preserving a discrete form of certain steady states exactly. In the above derivation, we implicitly fixed some choices within the scheme. When fixing the equilibrium subcell profile $U_{eq,i}(x)$, we chose exact integration in Eq. (38) to match with the *i*-th cell average. Instead, a quadrature rule, e.g., the midpoint rule, could be used. Similarly, we chose the exact solution Eq. (39) as the equilibrium subcell profile. As suggested by Roe (1987), Mellema et al. (1991) and Eulderink and Mellema (1995), one could chose an approximate solution Eq. (37) as the equilibrium subcell profile. In the next section, we present a general framework for the construction of well-balanced high-order finite volume schemes. At the root, it is based on a high-order generalization of the piecewise steady reconstruction idea.

We remark that fractional step or splitting methods could also be adapted to improve their performance near steady states. This can be achieved by carefully matching the boundary conditions used in the conservation law evolution Eq. (22a) and the source term integration Eq. (22b). However, we shall not pursue this idea in the sequel, and we refer to LeVeque (1986, 2002) and references therein for a general procedure.

### Well-balanced finite volume schemes

From the linear advection–reaction example, we see that the idea of a piecewise steady solution representation can lead to a finite volume scheme capable of preserving a steady state exactly, i.e., a well-balanced finite volume scheme. In the following, we present a simple recipe for constructing arbitrarily high-order well-balanced finite volume schemes in a systematic manner. The recipe relies on a high-order generalization of the piecewise steady reconstruction and a special discretization of the source terms guaranteeing the discrete preservation of steady states. We stress that the recipe is a humbly distilled version of the methodologies found in the vast literature about well-balanced finite volume schemes given in Sect. 1.

The principle of well-balanced finite volume methods based on piecewise steady reconstruction is to decompose the solution into an equilibrium part and a (not necessarily small^5^) perturbation part

43 $$\begin{aligned} \varvec{u}(x) = \varvec{u}_{eq}(x) + \delta \varvec{u}(x) , \end{aligned}$$

where the equilibrium part $\varvec{u}_{eq}(x)$ fulfills the steady-state balance

44 $$\begin{aligned} \frac{\partial }{\partial x} \varvec{f}\left( \varvec{u}_{eq}(x) \right) = \varvec{s} \left( \varvec{u}_{eq}(x) \right) . \end{aligned}$$

One obvious requirement for the piecewise steady reconstruction is thus the ability to compute such steady states. It turns out that this is difficult in general. However, we will tacitly assume that the differential equation Eq. (44) can be solved (exactly or approximately) for certain steady states

45 $$\begin{aligned} \varvec{U}_{eq}(x) = \varvec{u}_{eq}(x) + \mathcal {O}(\varDelta x^{\epsilon }) . \end{aligned}$$

Here $\epsilon$ denotes the spatial order of accuracy of the computed equilibrium solution. If Eq. (44) can be solved analytically for certain steady states, we may slightly abuse the notation and set $\epsilon = \infty$.

Solving for equilibrium is usually the main challenge when designing a well-balanced scheme. The difficulty depends strongly on the considered balance law and the associated steady states. For the linear advection–reaction equation, there is only one steady state Eq. (39) and it is known analytically. The Euler equations of fluid dynamics feature a myriad of steady states. We will look at one particular class that arises when considering the Euler equations in spherical symmetry in Sect. 2.5. Section 3 will look at the steady states that occur when the considered fluid is subject to gravitational forces. Luckily, one is generally not interested in all possible steady states within one practical simulation. Hence, it is often sufficient to design well-balanced schemes for certain stationary states of practical interest. The solvability of Eq. (44) is then restricted to these cases to which we will refer loosely as the *steady states of interest* in the following.

Next, we describe the modifications to the standard reconstruction and source term integration procedures of Godunov-type finite volume schemes for the homogeneous equations to build a well-balanced scheme for the steady states of interest. This involves the subtle correction of the reconstruction and source term integration that somehow incorporates the steady states of interest. We repeat the obvious that the following developments evidently hinge on the computability of the steady states of interest.

#### Piecewise steady reconstruction $\mathcal{WR}$

As in a standard finite volume scheme, within each cell a subcell profile $\varvec{U}_{i}(x)$ has to be reconstructed from the cell averages $\{\overline{\varvec{U}}_{k}\}$. A piecewise steady reconstruction $\mathcal{WR}$ is then given by the decomposition

46 $$\begin{aligned} \varvec{U}_{i}(x) = {\mathcal{WR}} \left( x; \left\{ \overline{\varvec{U}}_{k} \right\} _{k \in S_{i}} \right) = \varvec{U}_{eq,i}(x) + \delta \varvec{U}_{i}(x) , \end{aligned}$$

where $\varvec{U}_{eq,i}(x)$ and $\delta \varvec{U}_{i}(x)$ denote the local equilibrium and perturbation reconstruction parts in cell $\varOmega _{i}$, respectively. The stencil of the piecewise steady reconstruction is denoted as previously by $S_{i}$. We now describe each part in detail.

Within each cell $\varOmega _{i}$, the local equilibrium reconstruction $\varvec{U}_{eq,i}(x)$ is determined by fitting an equilibrium solution $\varvec{U}_{eq}(x)$ among the steady states of interest to the cell average $\overline{\varvec{U}}_{i}$. Since the cell average $\overline{\varvec{U}}_{i}$ may be arbitrarily far from a steady state of interest, this is done in two substeps. The first substep consists of projecting $\overline{\varvec{U}}_{i}$ onto a cell average $\overline{\varvec{U}}_{eq,i}$ consistent with the steady states of interest. The second substep determines the local equilibrium reconstruction $\varvec{U}_{eq,i}(x)$ in cell $\varOmega _{i}$ by matching an equilibrium profile Eq. (45) to the equilibrium projected cell average $\overline{\varvec{U}}_{eq,i}$

47 $$\begin{aligned} \frac{1}{\varDelta x} {\mathcal {Q}}_{i}\left( \varvec{U}_{eq,i} \right) = \overline{\varvec{U}}_{eq,i} , \end{aligned}$$

where ${\mathcal {Q}}_{i}$ denotes a *q*-th order accurate quadrature rule over cell $\varOmega _{i}$. We allow that the matching Eq. (47) is done exactly (using exact integration) and again slightly abuse the notation by setting $q = \infty$. For instance, the matching was done exactly with Eq. (38) in the linear advection–reaction example of Sect. 2.3. This results in a $\min (\epsilon , q)$-th order accurate local equilibrium reconstruction within each cell. However, the difficulty of this equilibrium projection and matching depends strongly on the balance law and steady states of interest. Some concrete examples are provided in Sects. 2.5 and 3. In addition, it is important to realize that not every given cell average must correspond to an equilibrium among the steady states of interest. Indeed, the solution may be far from a steady state. Therefore, the possibility that the local equilibrium reconstruction does not succeed must be taken into account. In that case, the local equilibrium reconstruction is simply set to zero $\varvec{U}_{eq,i}(x) \equiv 0$.

The local equilibrium perturbation $\delta \varvec{U}_{i}(x)$ within each cell $\varOmega _{i}$ is obtained by extrapolating the cell’s local equilibrium profile $\varvec{U}_{eq,i}(x)$ to neighboring cells, where it is compared with their cell averages. This senses how much the neighboring cells are perturbed with respect to the equilibrium in cell $\varOmega _{i}$. Cell-averages of these equilibrium perturbations can then be fed to any standard *r*-th order accurate piecewise polynomial reconstruction procedure to recover a local equilibrium perturbation profile as

48 $$\begin{aligned} \delta \varvec{U}_{i}(x) = \mathcal {R} \left( x; \left\{ \overline{\varvec{U}}_{k} - \frac{1}{\varDelta x} {\mathcal {Q}}_{k}(\varvec{U}_{eq,i}) \right\} _{k\in S_{i}} \right) . \end{aligned}$$

Like for the standard reconstruction procedure Eq. (16), the equilibrium perturbation reconstruction can also be performed in local characteristic variables.

**Fig. 5:**
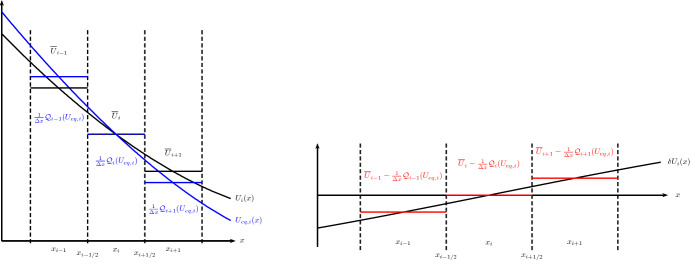
Sketch of the piecewise steady reconstruction of some scalar quantity from the cell averages $\{\overline{U}_{k}\}$. For the illustration, we assume that the steady-state projection of the cell averages is trivial $\overline{U}_{eq,i} = \overline{U}_{i}$ as for the steady states of the linear advection–reaction equation. Left panel: At the beginning of the reconstruction process, we are given the cell averages of the *i*-th cell and its immediate neighbors (solid black piecewise constant lines). The equilibrium reconstruction $U_{eq,i}(x)$ is built such that it matches the cell average $\overline{U}_{i}$ by Eq. (47) and extrapolated to the neighboring cells $k=i\pm 1$ (solid blue line). The cell averages of $U_{eq,i}(x)$ are then computed in the neighboring cells $k=i\pm 1$ (solid blue piecewise constant lines) and the cell-averaged equilibrium perturbations as seen from the *i*-th cell are computed. Right panel: The equilibrium perturbation $\delta U_{i}(x)$ is reconstructed by a standard reconstruction procedure Eq. (48). Finally, by combining the equilibrium $U_{eq,i}(x)$ and perturbation $\delta U_{i}(x)$ reconstruction as Eq. (46) one obtains the equilibrium-preserving piecewise steady reconstruction $U_{i}(x)$ in the left panel (solid black line)

The piecewise steady reconstruction ${\mathcal{WR}}$ (Eq. (46)) is illustrated in Fig. 5 for a scalar quantity. The reconstruction is $\min (\epsilon , q, r)$-th order accurate close or far from the steady states of interest (for smooth enough solutions, of course). It is intuitively clear that the local equilibrium perturbation $\delta \varvec{U}_{i}(x)$ vanishes if cell averages of a steady state of interest are fed to the piecewise steady reconstruction. Hence, a $\min (\epsilon , q)$-th order accurate discrete form of the steady states of interest is exactly reconstructed by the piecewise steady reconstruction. A proof is sketched in Sect. 2.4.3. Also note that if we set $\varvec{U}_{eq,i}(x) \equiv 0$, then ${\mathcal{WR}}$ automatically reduces to the standard piecewise polynomial reconstruction procedure $\mathcal {R}$. This is important in practice when there exists no solution of Eq. (47), i.e., no local equilibrium solution matching with the given cell’s average is found.

A possible variation of the piecewise steady reconstruction found in the literature is given by

49 $$\begin{aligned} \varvec{U}_{i}(x) = \widetilde{{\mathcal{WR}}} \left( x; \left\{ \overline{\varvec{U}}_{k} \right\} _{k \in S_{i}} \right) = \varvec{U}_{eq,i}(x) \left( 1 + \widetilde{\delta \varvec{U}}_{i}(x) \right) , \end{aligned}$$

which separates the solution into local equilibrium and *relative* perturbation parts (e.g., Chandrashekar and Klingenberg 2015; Berberich et al. 2019). The local equilibrium reconstruction $\varvec{U}_{eq,i}(x)$ is obtained with the same two substeps as above. The local relative equilibrium perturbation is computed by

50 $$\begin{aligned} \widetilde{\delta \varvec{U}}_{i}(x) = \mathcal {R} \left( x; \left\{ \frac{ \overline{\varvec{U}}_{k} - {\mathcal {Q}}_{k}(\varvec{U}_{eq,i}) / \varDelta x}{{\mathcal {Q}}_{k}(\varvec{U}_{eq,i}) / \varDelta x} \right\} _{k\in S_{i}} \right) , \end{aligned}$$

where the expression is to be understood component-wise. One drawback of this form is that it does not automatically reduce to a standard reconstruction if (some components of) the local equilibrium $\varvec{U}_{eq,i}(x)$ vanishes. In that case, one simply switches to a standard reconstruction (of these components) with some additional implementation logic. If the reconstruction is not sensitive to the shift with a constant,

51 $$\begin{aligned} \mathcal {R}\left( x; \left\{ \overline{Q}_{k} + C \right\} _{k\in S_{i}} \right) = \mathcal {R}\left( x; \left\{ \overline{Q}_{k} \right\} _{k\in S_{i}} \right) \end{aligned}$$

for any constant *C* and cell averages $\{\overline{Q}_{k}\}$, then Eq. (49) can be rewritten as

52 $$\begin{aligned} \varvec{U}_{i}(x) = \widetilde{{\mathcal{WR}}} \left( x; \left\{ \overline{\varvec{U}}_{k} \right\} _{k \in S_{i}} \right) = \varvec{U}_{eq,i}(x) ~ \widetilde{\delta \varvec{U}}_{i}(x) \end{aligned}$$

and the relative equilibrium perturbation Eq. (50) as

53 $$\begin{aligned} \widetilde{\delta \varvec{U}}_{i}(x) = \mathcal {R} \left( x; \left\{ \frac{\overline{\varvec{U}}_{k}}{{\mathcal {Q}}_{k}(\varvec{U}_{eq,i}) / \varDelta x} \right\} _{k\in S_{i}} \right) . \end{aligned}$$

Most reconstruction methods possess property Eq. (51) because non-oscillating behavior is usually enforced by limiting first and higher derivatives of the reconstruction polynomial and these are not affected by the addition of a constant. Both forms of the relative piecewise steady reconstruction share similar properties to the “absolute” one Eq. (46). An example is given in Sect. 3.3.1.

#### Well-balanced source term discretization $\mathcal{WS}\mathcal{}$

A direct numerical integration of the source term as in Eq. (20) will in general not lead to a well-balanced scheme. Instead, one uses the previously introduced piecewise steady reconstruction that decomposes the solution into an equilibrium and a perturbation part to perform the following seemingly frivolous manipulation

54 $$\begin{aligned} \varvec{s}(\varvec{U}_{i}) = \varvec{s}(\varvec{U}_{eq,i}) + \varvec{s}(\varvec{U}_{i}) - \varvec{s}(\varvec{U}_{eq,i}) , \end{aligned}$$

which simply adds and subtracts the source term evaluated with the local equilibrium reconstruction $\varvec{U}_{eq,i}$ within cell $\varOmega _{i}$. As suggested, e.g., by Huang and Liu (1986), Audusse et al. (2004), and Botta et al. (2004), the equilibrium part fulfills the steady-state balance by construction,

55 $$\begin{aligned} \frac{\partial }{\partial x} \varvec{f}\left( \varvec{U}_{eq,i}(x) \right) = \varvec{s} \left( \varvec{U}_{eq,i}(x) \right) , \end{aligned}$$

and can be trivially integrated by applying the fundamental theorem of calculus. The cell average of the source term Eq. (54) can therefore be approximated by applying exact integration to the equilibrium part and numerical integration to the perturbation part as follows

56 $$\begin{aligned} \overline{\varvec{S}}_{i} = \left. \frac{1}{\varDelta x} \varvec{f}(\varvec{U}_{eq,i}(x)) \right| _{x_{i-1/2}}^{x_{i+1/2}} + \frac{1}{\varDelta x} {\mathcal {Q}}_{i} \left( \varvec{s}(\varvec{U}_{i}(x)) - \varvec{s}(\varvec{U}_{eq,i}(x)) \right) . \end{aligned}$$

Here ${\mathcal {Q}}_{i}$ denotes a *q*-th order accurate quadrature rule over cell $\varOmega _{i}$. Note that ${\mathcal {Q}}_{i}$ may be different from the quadrature rule used in the piecewise steady reconstruction. We will refer to it with the same symbol since the same quadrature rule is typically used.

Equation (56) results in a $\min (\epsilon ,q,r,s)$-th order accurate discretization of the source term. At a steady state of interest (i.e., $\varvec{U}_{i} \equiv \varvec{U}_{eq,i}$), Eq. (56) reduces to

57 $$\begin{aligned} \overline{\varvec{S}}_{i} = \frac{1}{\varDelta x} \left( \varvec{f}(\varvec{U}_{eq,i}(x_{i+1/2})) - \varvec{f}(\varvec{U}_{eq,i}(x_{i-1/2})) \right) . \end{aligned}$$

As we will see below, this is crucial for the well-balanced property of the scheme. Moreover, note that if the local equilibrium reconstruction part $\varvec{U}_{eq,i}(x)$ vanishes, the above source term discretization automatically reduces to the standard one in Eq. (20). This is again important in practice when no local equilibrium matching with the given cell average is found (i.e., no solution to Eq. (47) is found).

An alternative form of the well-balanced source discretization is based on Richardson extrapolation. The idea is to write the source term as

58 $$\begin{aligned} \varvec{s}(\varvec{U}_{i}) = \frac{\varvec{s}(\varvec{U}_{i})}{\varvec{s}(\varvec{U}_{eq,i})} \varvec{s}(\varvec{U}_{eq,i}) = \frac{\varvec{s}(\varvec{U}_{i})}{\varvec{s}(\varvec{U}_{eq,i})} \frac{\partial }{\partial x} \varvec{f}\left( \varvec{U}_{eq,i} \right) , \end{aligned}$$

which has to be understood component-wise. The fact that the equilibrium part $\varvec{U}_{eq,i}$ fulfills the steady-state balance by construction is used in the second equality. However, note that rewriting the source term in this way may not be possible for all the components of a particular system of balance laws, because they are trivially fulfilled at the steady states of interest. Hence, they are not relevant for the construction of a well-balanced scheme and can be discretized in a standard way. For the sake of presentation, we ignore this subtlety in the derivation of the well-balanced source term discretization based on this form. An illustrative example of this alternative well-balanced source term discretization is provided in Sect. 3.3.1.

Consider the following second-order approximation of the cell-averaged source term based on the form above

59 $$\begin{aligned} \begin{aligned} \overline{\varvec{S}}_{i}&= \frac{1}{\varDelta x} T_{i} \left( \frac{\varvec{s}(\varvec{U}_{i})}{\varvec{s}(\varvec{U}_{eq,i})} \frac{\partial }{\partial x} \varvec{f}\left( \varvec{U}_{eq,i} \right) \right) \\&= \frac{1}{2} \left( \frac{\varvec{s}(\varvec{U}_{i}(x_{i-1/2}))}{\varvec{s}(\varvec{U}_{eq,i}(x_{i-1/2}))} + \frac{\varvec{s}(\varvec{U}_{i}(x_{i+1/2}))}{\varvec{s}(\varvec{U}_{eq,i}(x_{i+1/2}))} \right) \left. \frac{1}{\varDelta x} \varvec{f}(\varvec{U}_{eq,i}(x)) \right| _{x_{i-1/2}}^{x_{i+1/2}} , \end{aligned} \end{aligned}$$

where we introduce the symbol $T_{i}$ for this particular quadrature rule (due to its resemblance with the trapezoidal rule). At a steady state of interest $\varvec{U}_{i} \equiv \varvec{U}_{eq,i}$, this clearly reduces to Eq. (57) which is again crucial for well-balancing as we shall see below. Unfortunately, it is still only a second-order source term discretization. To overcome this limitation, Noelle et al. (2006) ingeniously suggest to use Richardson extrapolation. Let us introduce a composite quadrature rule based on Eq. (59). The cell $\varOmega _{i}$ is subdivided in $N_{c}$ uniform subintervals $\varOmega _{i}^{j} = [x_{i,j-1/2} , x_{i,j+1/2}]$ of size $h = \varDelta x / N_{c}$ with $x_{i,j-1/2} = x_{i-1/2} + j h$ ($j=0, \dots , N_{c}$). By applying the quadrature rule $T_{i}$ to each subinterval and summing up, we obtain the following composite quadrature rule

60 $$\begin{aligned} \begin{aligned} \overline{\varvec{S}}_{i}&= \frac{1}{\varDelta x} T_{i}^{N_{c}} \left( \frac{\varvec{s}(\varvec{U}_{i})}{\varvec{s}(\varvec{U}_{eq,i})} \frac{\partial }{\partial x} \varvec{f}\left( \varvec{U}_{eq,i} \right) \right) \\&= \frac{1}{\varDelta x} \sum _{j=1}^{N_{c}} \frac{h}{2} \left( \frac{\varvec{s}(\varvec{U}_{i}(x_{i,j-1/2}))}{\varvec{s}(\varvec{U}_{eq,i}(x_{i,j-1/2}))} + \frac{\varvec{s}(\varvec{U}_{i}(x_{i,j+1/2}))}{\varvec{s}(\varvec{U}_{eq,i}(x_{i,j+1/2}))} \right) \left. \frac{1}{h} \varvec{f}(\varvec{U}_{eq,i}(x)) \right| _{x_{i,j-1/2}}^{x_{i,j+1/2}} . \end{aligned} \end{aligned}$$

This again reduces to Eq. (57) at a steady state of interest by telescoping of the sum, but it is still only second-order accurate. However, Noelle et al. (2006) note that the quadrature rule $T_{i}$ is also symmetric and therefore possesses an asymptotic error expansion of the form

61 $$\begin{aligned} T_{i}^{N_{c}}(f) = \int _{\varOmega _{i}} f(x) {\mathrm {d}}x + c_{1} \left( \frac{\varDelta x}{N_{c}} \right) ^{2} + c_{2} \left( \frac{\varDelta x}{N_{c}} \right) ^{4} + c_{3} \left( \frac{\varDelta x}{N_{c}} \right) ^{6} + \dots \end{aligned}$$

for any (smooth enough) function *f*. Richardson extrapolation then combines the $T_{i}^{N_{c}}$ to cancel out increasingly higher error terms in the expansion. For example, fourth- and a sixth-order accurate quadrature rules are readily obtained:

62 $$\begin{aligned} \begin{aligned} \frac{T_{i}^{2}(f) - T_{i}^{1}(f)}{3}&= \int _{\varOmega _{i}} f(x) {\mathrm {d}}x + \mathcal {O}(\varDelta x)^{4} , \\ \frac{ 64 T_{i}^{4}(f) - 20 T_{i}^{2}(f) + T_{i}^{1}(f)}{45}&= \int _{\varOmega _{i}} f(x) {\mathrm {d}}x + \mathcal {O}(\varDelta x)^{6} . \end{aligned} \end{aligned}$$

Thus, arbitrary high-order well-balanced source term discretizations can be obtained from the alternative form Eq. (59). Although it possesses similar properties as the well-balanced source term discretization Eq. (56), one drawback of the alternative form is that it does not automatically reduce to a standard source term discretization in case the local equilibrium part vanishes in the piecewise steady reconstruction (i.e., no solution to Eq. (47) is found). However, this can easily be handled with some additional implementation logic.

#### Assembling a well-balanced finite volume scheme

A well-balanced finite volume scheme Eq. (7) for the one-dimensional balance law Eq. (4) is now easily assembled with the formerly described components:

1. A $\min (\epsilon ,q,r)$-th order accurate piecewise steady reconstruction ${\mathcal{WR}}$ (Eq. (46), Eq. (49) or Eq. (52)).
2. A consistent and Lipschitz continuous numerical flux function $\mathcal {F}$ (Eq. (17)).
3. An unsplit $\min (\epsilon ,q,r,s)$-th order accurate well-balanced source term discretization $\mathcal{WS}\mathcal{}$ (Eq. (56) or Eqs. (60) and (62)).
4. A $\tau$-th order accurate time integrator $\mathcal {T}$.

This results in a $\min (\epsilon ,q,r,s,\tau )$-th order accurate well-balanced finite volume scheme (for smooth enough solutions). The scheme preserves exactly a $\min (\epsilon ,q)$-th order accurate discrete form of the steady states of interest (up to machine precision). Furthermore, such a well-balanced scheme automatically falls back to a standard high-order finite volume scheme if the local equilibrium reconstruction part vanishes.^6^ This guarantee is important in practice since one is assured that if the piecewise steady reconstruction fails to determine a local equilibrium profile (because it may not exist), the scheme reduces decently to a standard scheme without any loss of accuracy and robustness.

To round off this section, we demonstrate the well-balanced property of such a scheme, that is, its ability to preserve exactly (up to machine precision) a discrete form $\varvec{U}_{eq}(x)$ of the steady states of interest $\varvec{u}_{eq}(x)$ it was designed for.^7^ For simplicity, we assume that both $\varvec{u}_{eq}(x)$ and its approximation $\varvec{U}_{eq}(x)$ are continuous. As we shall see below, this requirement can easily be waived. Let the scheme be given cell averages $\{\overline{\varvec{U}}_{i}\}$ of such a steady state of interest. These cell averages are computed from a given steady state $\varvec{u}_{eq}(x)$ approximated discretely by $\varvec{U}_{eq}(x)$ with

63 $$\begin{aligned} \overline{\varvec{U}}_{i} = \frac{1}{\varDelta x} {\mathcal {Q}}_{i}(\varvec{U}_{eq}(x)) , \end{aligned}$$

where ${\mathcal {Q}}_{i}$ denotes the same *q*-th order quadrature rule as used when matching the local equilibrium profile with the cell averages in the piecewise steady reconstruction Eq. (47). We shall term such initial data as *well-prepared* initial data. Given such well-prepared initial data, we reciprocally assume that the local equilibrium reconstruction Eq. (47) recovers within every cell $\varOmega _{i}$ the restriction of $\varvec{U}_{eq}(x)$ in respective cell,

64 $$\begin{aligned} \varvec{U}_{eq,i}(x) = \left. \varvec{U}_{eq}(x) \right| _{x \in \varOmega _{i}} , \end{aligned}$$

and that its extrapolation over the computational domain $\varOmega$ recovers $\varvec{U}_{eq}(x)$ everywhere, i.e.,

65 $$\begin{aligned} \varvec{U}_{eq,i}(x) = \varvec{U}_{eq}(x)\, {\text { for }}\, x \in \varOmega . \end{aligned}$$

Of course, this assumption needs to be verified for the particular balance law and steady states of interest, and represents the core challenge in the construction of a well-balanced scheme. Taking this assumption for granted, it is obvious that the piecewise steady reconstruction ${\mathcal{WR}}$ (Eq. (46)) recovers the given steady state $\varvec{U}_{eq}(x)$ in every cell as the local equilibrium perturbation vanishes everywhere, i.e., $\delta \varvec{U}_{i}(x) \equiv 0$, and we have

66 $$\begin{aligned} \varvec{U}_{i}(x) = \left. \varvec{U}_{eq}(x) \right| _{x \in \varOmega _{i}} . \end{aligned}$$

The same holds true for the alternative piecewise steady reconstructions Eq. (49) or Eq. (52)) with slightly adapted arguments. For the numerical flux, we therefore have

67 $$\begin{aligned} \varvec{F}_{i+1/2} = \mathcal {F}(\varvec{U}_{i+1/2-} , \varvec{U}_{i+1/2+}) = \varvec{f}(\varvec{U}_{eq}(x_{i+1/2})) \end{aligned}$$

due to the fitting of the piecewise steady reconstruction ${\mathcal{WR}}$ at every cell interface

68 $$\begin{aligned} \varvec{U}_{i+1/2-} = \varvec{U}_{i}(x_{i+1/2}) = \varvec{U}_{eq}(x_{i+1/2}) = \varvec{U}_{i+1}(x_{i+1/2}) = \varvec{U}_{i+1/2+} . \end{aligned}$$

Similarly for the source term discretization $\mathcal{WS}\mathcal{}$ Eq. (56), we have

69 $$\begin{aligned} \begin{aligned} \overline{\varvec{S}}_{i}&= \left. \frac{1}{\varDelta x} \varvec{f}(\varvec{U}_{eq,i}(x)) \right| _{x_{i-1/2}}^{x_{i+1/2}} + \frac{1}{\varDelta x} {\mathcal {Q}}_{i} \left( \varvec{s}(\varvec{U}_{i}(x)) - \varvec{s}(\varvec{U}_{eq,i}(x)) \right) \\&= \frac{1}{\varDelta x} \left( \varvec{f}(\varvec{U}_{eq}(x_{i+1/2})) - \varvec{f}(\varvec{U}_{eq}(x_{i-1/2})) \right) . \end{aligned} \end{aligned}$$

The alternative source term discretization Eqs. (60) and (62) likewise reduces to the above expression. Plugging Eqs. (67) and (69) into Eq. (7), one obtains

$$\begin{aligned} \frac{{\mathrm {d}}\overline{\varvec{U}}_{i}}{{\mathrm {d}}t} = - \frac{1}{\varDelta x} \left( \varvec{F}_{i+1/2} - \varvec{F}_{i-1/2} \right) + \overline{\varvec{S}}_{i} = 0 , \end{aligned}$$

and therefore the scheme is well-balanced as claimed, i.e., it preserves a $\min (\epsilon , q)$-th order accurate discrete form of the steady states of interest exactly (up to machine precision).

We assumed that the steady states of interest and their discrete approximation are continuous in Eq. (68). However, it is straightforward to generalize the well-balanced scheme (and the above demonstration) to steady states with stationary discontinuities located at cell interfaces. For this purpose, some mild additional requirements for the numerical flux function $\mathcal {F}$ and the standard piecewise polynomial reconstruction procedure $\mathcal {R}$ are necessary: (i) the numerical flux $\mathcal {F}$ has to be able to resolve exactly the (stationary) discontinuities allowed by the steady states of interest, and (ii) the reconstruction procedure $\mathcal {R}$ has to reduce to a piecewise constant reconstruction near isolated discontinuities at cell interfaces. Both requirements ensure that at the stationary discontinuities the numerical flux agrees with the exact flux like in Eq. (66).

### Example: the Euler equations in spherical symmetry

As a simple and practical example of the construction of a well-balanced finite volume scheme, we consider the Euler equations in spherical symmetry

70a $$\begin{aligned} \frac{\partial \rho }{\partial t} + \frac{1}{r^{2}} \frac{\partial }{\partial r}\left( r^{2} \rho v \right)&= 0 , \end{aligned}$$

70b $$\begin{aligned} \frac{\partial \rho v}{\partial t} + \frac{1}{r^{2}} \frac{\partial }{\partial r}\left( r^{2} \rho v^{2} \right) + \frac{\partial p}{\partial r}&= 0 , \end{aligned}$$

70c $$\begin{aligned} \frac{\partial E}{\partial t} + \frac{1}{r^{2}} \frac{\partial }{\partial r}\left[ r^{2} \left( E + p \right) v \right]&= 0 , \end{aligned}$$

expressing the conservation of mass, momentum and energy. Here *r* is the radial coordinate, $\rho$ the mass density, *v* the radial velocity, $E = \rho e + \frac{1}{2} \rho v^{2}$ the total fluid energy composed of internal and kinetic energy densities, and *p* the pressure. The latter is related to the density $\rho$ and specific internal energy *e* through an equation of state $p = p(\rho ,e)$.

A computationally convenient form of Eq. (70) is given by

71 $$\begin{aligned} \frac{\partial \varvec{u}}{\partial t} + \frac{\partial (A \varvec{f})}{\partial V} = \varvec{s} , \end{aligned}$$

where the vector of conserved variables, fluxes and source terms are

72 $$\begin{aligned} \varvec{u} = \begin{bmatrix} \rho \\ \rho v \\ E \end{bmatrix} , \; \varvec{f}(\varvec{u}) = \begin{bmatrix} \rho v \\ \rho v^{2} + p \\ (E + p) v \end{bmatrix} , \; \varvec{s}(\varvec{u}) = \begin{bmatrix} 0 \\ p \\ 0 \end{bmatrix} \frac{\partial A}{\partial V} = \begin{bmatrix} 0 \\ \frac{2 p}{r} \\ 0 \end{bmatrix} , \end{aligned}$$

and the area and volume functions are

73 $$\begin{aligned} A(r) = 4 \pi r^{2} \quad {\text {and}} \quad V(r) = \frac{4 \pi }{3} r^{3} . \end{aligned}$$

This form is particularly convenient because the fluxes take exactly the same form as in the one-dimensional planar geometry case. Hence, the same numerical fluxes can directly be used. The drawback of this form is the introduction of a geometric source term that becomes singular near the origin. Furthermore, note that the source term may depend non-linearly on the conserved variables through the equation of state.

A particular steady state of Eq. (70) and Eq. (71) is a resting fluid with uniform density and pressure profile. It is of course highly desirable that a numerical scheme faithfully reproduces this seemingly trivial equilibrium. The steady state of interest $\varvec{u}_{eq}$ is therefore simply

74 $$\begin{aligned} \varvec{u}_{eq} = \begin{bmatrix} \rho _{eq} \\ 0 \\ \rho e_{eq} \end{bmatrix} , \end{aligned}$$

where $\rho _{eq} = {\text {const}}$ is the constant density and $\rho e_{eq} = {\text {const}}$ the constant internal energy density, respectively. It clearly fulfills

75 $$\begin{aligned} \frac{\partial }{\partial V}A \varvec{f}(\varvec{u}_{eq}) = \frac{1}{r^2} \frac{\partial }{\partial r} \begin{bmatrix} 0 \\ r^2 p_{eq} \\ 0 \end{bmatrix} = \begin{bmatrix} 0 \\ \frac{2 p_{eq}}{r} \\ 0 \end{bmatrix} = \varvec{s}(\varvec{u}_{eq}) , \end{aligned}$$

where $p_{eq} = p(\rho _{eq},\rho e_{eq}) = \text {const}$ is the constant equilibrium pressure.

A straightforward discretization of Eq. (71) on a spherical domain $D = [R_{0},R_{1}]$, $0 \le R_{0} < R_{1}$, with a semi-discrete finite volume method gives for the *i*-th cell

76 $$\begin{aligned} \frac{{\mathrm {d}}\overline{\varvec{U}}_{i}}{{\mathrm {d}}t} = - \frac{1}{\varDelta V_{i}} \left( A_{i+1/2} \varvec{F}_{i+1/2} - A_{i-1/2} \varvec{F}_{i-1/2} \right) + \overline{\varvec{S}}_{i} . \end{aligned}$$

Here $\overline{\varvec{U}}_{i}$ denotes the approximate cell average of the conserved variables over a (spherical shell) cell $\varOmega _{i} = [r_{i-1/2},r_{i+1/2}]$ with inner/outer radius $r_{i\pm 1/2}$ of volume $\varDelta V_{i} = V(r_{i+1/2}) - V(r_{i-1/2})$

77 $$\begin{aligned} \overline{\varvec{U}}_{i}(t) \approx \overline{\varvec{u}}_{i}(t) = \frac{1}{\varDelta V_{i}} \int _{\varOmega _{i}} \varvec{u}(r,t) {\mathrm {d}}V . \end{aligned}$$

The fluxes through the inner/outer (spherical shell) cell boundary of area $A_{i\pm 1/2} = A(r_{i\pm 1/2})$ are approximated by a numerical flux function

78 $$\begin{aligned} F_{i\pm 1/2} = \mathcal {F}(\varvec{U}_{i\pm 1/2-},\varvec{U}_{i\pm 1/2+}) , \end{aligned}$$

e.g., the Rusanov flux Eq. (19), and the cell interface extrapolated point values of the conserved variables $\varvec{U}_{i\pm 1/2-}/\varvec{U}_{i\pm 1/2+}$ are obtained from a reconstruction procedure. For simplicity of the example, let’s fix spatial accuracy to second order by choosing a piecewise linear reconstruction centered at the (spherical shell) cell center^8^$r_{i} = (r_{i-1/2} + r_{i+1/2})/2$,

79 $$\begin{aligned} \varvec{U}_{i}(r) = \mathcal {R}\left( r; \left\{ \overline{\varvec{U}}_{k}\right\} _{k \in S_{i}}\right) = \overline{\varvec{U}}_{i} + D\varvec{U}_{i} ~ (r - r_{i}) , \end{aligned}$$

where $S_{i} = \{i-1,i,i+1\}$ is the stencil and the limited slopes can be computed with the generalized $\mathrm {minmod}$ slope Eq. (14). Accordingly, we also choose the second-order accurate midpoint quadrature rule^9^ for approximating integrals of a function *f* over a (spherical shell) cell $\varOmega _{i}$:

80 $$\begin{aligned} {\mathcal {Q}}_{i}(f) = \varDelta V_{i} ~ f(r_{i}) . \end{aligned}$$

This immediately gives the following (naive) discretization of the geometric source term

81 $$\begin{aligned} \overline{\varvec{S}}_{i} = \frac{1}{\varDelta V_{i}} {\mathcal {Q}}_{i} (\varvec{s}(\varvec{U}_{i})) = \begin{bmatrix} 0 \\ \frac{2 p_{i}}{r_{i}} \\ 0 \end{bmatrix} , \end{aligned}$$

where $p_{i}$ is the pressure at the cell center. The latter is simply obtained by evaluating the piecewise linearly reconstructed conserved variables Eq. (79) at cell center

82 $$\begin{aligned} p_{i} = p\left( \rho _{i}(r_{i}),\rho e_{i}(r_{i})\right) = p(\overline{\rho }_{i},\rho e_{i}) \end{aligned}$$

with

83 $$\begin{aligned} \rho e_{i} = \rho e_{i}(r_{i}) = E_{i}(r_{i}) - \frac{\rho v_{i}(r_{i})^2}{2 \rho _{i}(r_{i})} = \overline{E}_{i} - \frac{\overline{\rho v}_{i}^2}{2 \overline{\rho }_{i}} , \end{aligned}$$

where the peculiarity that cell averages correspond to point values at cell center up to second-order accuracy is especially apparent. This concludes the description of a plain-vanilla finite volume scheme for the Euler equations in spherical symmetry.

We now construct a well-balanced finite volume scheme capable of preserving a resting fluid Eq. (74) exactly following the recipe in Sect. 2.4 based on the just described scheme. First, we need to devise a piecewise steady reconstruction procedure for the resting fluid equilibrium, i.e., our steady state of interest we wish to preserve. We begin with the local equilibrium reconstruction part. The first substep in the local equilibrium reconstruction is the projection of the cell averages onto equilibrium cell averages consistent with the resting fluid equilibrium. This substep is necessary because the averages could be arbitrarily far from the steady state of interest (i.e., non-vanishing radial momentum and kinetic energy densities), and it is simply accomplished by

84 $$\begin{aligned} \overline{\varvec{U}}_{eq,i} = \begin{bmatrix} \overline{\rho }_{i} \\ 0 \\ \rho e_{i} \end{bmatrix} . \end{aligned}$$

The equilibrium cell average of the density is simply set to the cell-averaged density and the equilibrium cell average of the momentum density is set to zero as is consistent with the steady state of interest. An expression for the cell average of the internal energy density is provided by Eq. (83). Although this is only spatially second-order accurate in general, it becomes exact when the fluid is at rest, thereby establishing consistency with the steady state of interest Eq. (74). The second substep is then to match a local equilibrium reconstruction $\varvec{U}_{eq,i}(r)$ to the cell’s $\varOmega _{i}$ average equilibrium projected conserved variables $\overline{\varvec{U}}_{eq,i}$ as in Eq. (47). This is indeed trivial given the constant nature of the considered steady state of interest

85 $$\begin{aligned} \varvec{U}_{eq,i}(r) = \overline{\varvec{U}}_{eq,i} = \begin{bmatrix} \overline{\rho }_{i} \\ 0 \\ \rho e_{i} \end{bmatrix} . \end{aligned}$$

The local equilibrium perturbation reconstruction Eq. (48) is then

86 $$\begin{aligned} \delta \varvec{U}_{i}(r) = \mathcal {R}\left( r; \left\{ \overline{\varvec{U}}_{k} - \overline{\varvec{U}}_{eq,i} \right\} _{k \in S_{i}}\right) = \left( \overline{\varvec{U}}_{i} - \overline{\varvec{U}}_{eq,i} \right) + D\delta \varvec{U}_{i} ~ (r - r_{i}) , \end{aligned}$$

where we used that $\varvec{U}_{eq,i}(r)$ is a simple constant, i.e.,

87 $$\begin{aligned} \overline{\varvec{U}}_{eq,i} = \frac{1}{\varDelta V_{k}} \int _{\varOmega _{k}} \varvec{U}_{eq,i}(r) {\mathrm {d}}V = \frac{1}{\varDelta V_{k}} {\mathcal {Q}}_{k}(\varvec{U}_{eq,i})\, {\text { for }}\, k \in S_{i} . \end{aligned}$$

As result, we obtain the following piecewise steady reconstruction

88 $$\begin{aligned} \varvec{U}_{i}(r) = \mathcal {W}\left( r; \left\{ \overline{\varvec{U}}_{k} \right\} _{k \in S_{i}}\right) = \varvec{U}_{eq,i}(r) + \delta \varvec{U}_{i}(r) = \overline{\varvec{U}}_{eq,i}+ \delta \varvec{U}_{i}(r) . \end{aligned}$$

In the last equality, we used again that $\varvec{U}_{eq,i}(r)$ is simply a constant.

However, the above piecewise steady reconstruction can be much simplified. The limited slopes $D\delta \varvec{U}_{i}$ can be reduced with the following observation

89 $$\begin{aligned} \frac{\overline{\varvec{U}}_{i+1} - \overline{\varvec{U}}_{i}}{\varDelta r} = \frac{ (\overline{\varvec{U}}_{i+1} - \overline{\varvec{U}}_{eq,i}) - (\overline{\varvec{U}}_{i } - \overline{\varvec{U}}_{eq,i})}{\varDelta r} , \end{aligned}$$

which means that the equilibrium $\overline{\varvec{U}}_{eq,i}$ drops out in the computation of the slopes Eq. (14). Hence, we have that the limited slopes of the local equilibrium perturbation reconstruction in Eq. (86) reduce to the slopes used in the standard piecewise linear reconstruction Eq. (79): $D\delta \varvec{U}_{i} = D\varvec{U}_{i}$. Now by combining this result with Eq. (86) and plugging it into Eq. (88), we obtain that the piecewise steady reconstruction simplifies to the standard piecewise linear reconstruction:

90 $$\begin{aligned} \varvec{U}_{i}(r) = \mathcal {W}\left( r; \left\{ \overline{\varvec{U}}_{k} \right\} _{k \in S_{i}}\right) = \mathcal {R}\left( r; \left\{ \overline{\varvec{U}}_{k}\right\} _{k \in S_{i}}\right) = \overline{\varvec{U}}_{i} + D\varvec{U}_{i} ~ (r - r_{i}) . \end{aligned}$$

Of course, this is not surprising as we simply subtract a constant from the data to be (piecewise linearly) reconstructed. It is nevertheless a welcome simplification when implementing the present scheme.

Finally, we construct the appropriate well-balanced source term discretization with Eq. (56):

91 $$\begin{aligned} \overline{\varvec{S}}_{i}&= \left. \frac{1}{\varDelta V_{i}} A(r) ~ \varvec{f}(\varvec{U}_{eq,i}(r)) \right| _{r_{i-1/2}}^{r_{i+1/2}} + \frac{1}{\varDelta V_{i}} {\mathcal {Q}}_{i}\left( \varvec{s}(\varvec{U}_{i}(r)) - \varvec{s}(\varvec{U}_{eq,i}(r)) \right) \nonumber \\&= \frac{A_{i+1/2} - A_{i-1/2}}{\varDelta V_{i}} \begin{bmatrix} 0 \\ p(\overline{\rho }_{i}, \rho e_{i}) \\ 0 \end{bmatrix} + \varvec{s}(\varvec{U}_{i}(r_{i})) - \varvec{s}(\varvec{U}_{eq,i}(r_{i})) \nonumber \\&= \frac{A_{i+1/2} - A_{i-1/2}}{\varDelta V_{i}} \begin{bmatrix} 0 \\ p_{i} \\ 0 \end{bmatrix} . \end{aligned}$$

We substituted the midpoint rule Eq. (80) in the second equality, and we used the fact that the pressure computed from the piecewise steady $\varvec{U}_{i}(r)$ and the local equilibrium reconstruction $\varvec{U}_{eq,i}(r)$ coincide at the cell center $r_{i}$ in the third equality (see Eqs. (82) and (83)).

**Fig. 6:**
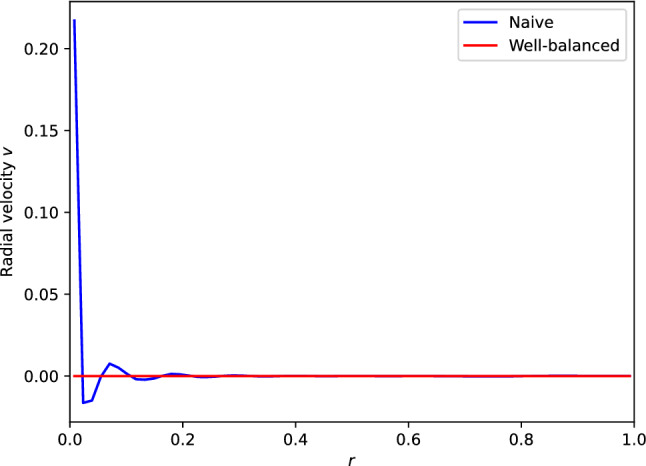
The figure shows the radial velocity after one time unit of a constant state with unit density and pressure ($\rho = p = 1$). The blue/red line corresponds to the results obtained by the standard naive/well-balanced second-order schemes from Sect. 2.5 with a resolution of $N=64$. In both simulations, solid wall boundary conditions were enforced at the upper boundary. From the plot it is obvious that the standard treatment of the geometric source term results in spurious velocity fluctuations near the origin. In contrast, the well-balanced scheme shows a still standing radial profile, as is expected

It is now straightforward to show that the just derived source term discretization Eq. (91) is indeed able to preserve a resting fluid with uniform density and pressure exactly. Hence, we have designed a well-balanced scheme for this particular steady state. This is confirmed by the numerical results displayed in Fig. 6.

We remark that the above spatially second-order accurate source term discretization Eq. (91) is well-known among the practitioners in the field (see, e.g., Mönchmeyer and Müller 1989; Li 2003; Skinner and Ostriker 2010; Wang and Johnsen 2013). The above expression for the geometric source term can also be motivated from the derivation of the momentum equation Eq. (72) which expresses the pressure gradient in Eq. (70b) with the following simple application of the product rule

92 $$\begin{aligned} \frac{\partial p}{\partial r} = \frac{\partial }{\partial V}(A p) - \frac{\partial A}{\partial V} p . \end{aligned}$$

Expressing $\frac{\partial A}{\partial V}$ in a discrete finite volume sense then immediately gives Eq. (91). However, the here described discretization is in principle extensible to arbitrary spatial orders of accuracy. It would be interesting to combine the above with high-order reconstruction procedures for orthogonal curvilinear coordinates devised by Mignone (2014) and Shadab et al. (2019) together with specifically designed weighted^10^ Gauss quadrature rules.

### Extension to several space dimensions

We now extend the one-dimensional recipe in Sect. 2.4 to build arbitrarily high-order well-balanced finite volume schemes for multidimensional systems of balance laws

93 $$\begin{aligned} \frac{\partial \varvec{u}}{\partial t} + \nabla \cdot {\textbf {\textsf {f}}} = \varvec{s} , \end{aligned}$$

where $\varvec{u}=\varvec{u}(\varvec{x},t)$ is the vector of conserved variables, ${\textbf {\textsf {f}}}={\textbf {\textsf {f}}}(\varvec{u})$ the flux tensor and $\varvec{s}=\varvec{s}(\varvec{u})$ the vector of source terms. As in the one-dimensional case, we tacitly assume that (i) the system is of hyperbolic nature (the Jacobian of the flux tensor $\varvec{n} \cdot \frac{\partial {\textbf {\textsf {f}}}}{\partial \varvec{u}}$ is diagonalizable with real eigenvalues for any direction $\varvec{n}$) and that (ii) the source term is not singular. For ease of presentation, we focus on the two-dimensional case in Cartesian coordinates

94 $$\begin{aligned} \frac{\partial \varvec{u}}{\partial t} + \frac{\partial \varvec{f}}{\partial x} + \frac{\partial \varvec{g}}{\partial y} = \varvec{s} , \end{aligned}$$

where $\varvec{f}=\varvec{f}(\varvec{u})$ and $\varvec{g}=\varvec{g}(\varvec{u})$ are the vectors of fluxes in *x*- and *y*-direction, i.e., the components of the flux tensor ${\textbf {\textsf {f}}}=[{\textbf {\textsf {f}}},\varvec{g}]^{T}$ in Cartesian coordinates. However, the extension to three dimensions and other coordinate systems is straightforward.

In the following subsection, we begin by concisely describing a standard finite volume discretization of the balance law Eq. (93) to introduce our notation. More comprehensive descriptions can be found in the excellent textbooks listed at the end of Sect. 2.1. The extension of the one-dimensional recipe to design well-balanced schemes in several space dimensions is presented in the subsequent subsections.

#### Finite volume discretization

We consider a rectangular spatial domain $\varOmega = [x_{\min },x_{\max }] \times [y_{\min },y_{\max }]$ discretized uniformly (for ease of presentation) by $N_{x}$ and $N_{y}$ cells or finite volumes in *x*- and *y*-direction, respectively. The cells are labeled by $\varOmega _{i,j} = \varOmega _{i} \times \varOmega _{j} = [x_{i-1/2},x_{i+1/2}] \times [y_{j-1/2},y_{j+1/2}]$, the constant cell sizes by $\varDelta x = x_{i+1/2} - x_{i-1/2}$ and $\varDelta y = y_{j+1/2} - y_{j-1/2}$, and the cell volumes by $\left| \varOmega _{i,j} \right| = \varDelta x \varDelta y$. We also introduce a non-directional cell size $h = \max (\varDelta x, \varDelta y)$ for convenience. The $x_{i} = (x_{i-1/2} + x_{i+1/2})/2$ and $y_{j} = (y_{j-1/2} + y_{j+1/2})/2$ denote the cell centers.

A semi-discrete finite volume scheme for the numerical approximation of Eq. (94) then takes the following form

95 $$\begin{aligned} \begin{aligned} \frac{{\mathrm {d}}}{{\mathrm {d}}t} \overline{\varvec{U}}_{i,j}&= {\mathcal {L}}(\overline{\varvec{U}})_{i,j} \\&= - \frac{1}{\varDelta x} \left( \varvec{F}_{i+1/2,j} - \varvec{F}_{i-1/2,j} \right) - \frac{1}{\varDelta y} \left( \varvec{G}_{i,j+1/2} - \varvec{G}_{i,j-1/2} \right) + \overline{\varvec{S}}_{i,j} , \end{aligned} \end{aligned}$$

where the $\overline{\varvec{U}}_{i,j}$ denote the approximate cell averages of the conserved variables,

96 $$\begin{aligned} \overline{\varvec{U}}_{i,j}(t) \approx \overline{\varvec{u}}_{i,j}(t) = \frac{1}{\left| \varOmega _{i,j} \right| } \int _{\varOmega _{i,j}} \varvec{u}(x,y,t) ~ {\mathrm {d}}x ~ {\mathrm {d}}y , \end{aligned}$$

the $\varvec{F}_{i\pm 1/2,j}$ and $\varvec{G}_{i,j\pm 1/2}$ are approximate facial averages of the fluxes through the cell boundary in respective direction,

97 $$\begin{aligned} \begin{aligned} \varvec{F}_{i\pm 1/2,j}(t)&\approx \frac{1}{\varDelta y} \int _{y_{j-1/2}}^{y_{j+1/2}} \varvec{f}(\varvec{u}(x_{i\pm 1/2},y,t)) ~ {\mathrm {d}}y \\ \varvec{G}_{i,j\pm 1/2}(t)&\approx \frac{1}{\varDelta x} \int _{x_{i-1/2}}^{x_{i+1/2}} \varvec{g}(\varvec{u}(x,y_{j\pm 1/2},t)) ~ {\mathrm {d}}x , \end{aligned} \end{aligned}$$

and the $\overline{\varvec{S}}_{i,j}$ are approximate cell averages of the source term

98 $$\begin{aligned} \overline{\varvec{S}}_{i,j}(t) \approx \overline{\varvec{s}}_{i,j}(t) = \frac{1}{\left| \varOmega _{i,j} \right| } \int _{\varOmega _{i,j}} \varvec{s}(\varvec{u}(x,y,t)) ~ {\mathrm {d}}x ~ {\mathrm {d}}y . \end{aligned}$$

The next paragraphs compactly describe the components of a generic finite volume scheme in several space dimensions. For the sake of presentation, we suppress the temporal dependence of the quantities.

*Reconstruction* The first task is to reconstruct an accurate subcell profile from the cell-averaged conserved variables. We denote such an *r*-th order accurate piecewise polynomial reconstruction procedure by

99 $$\begin{aligned} \varvec{U}_{i,j}(x,y) = \mathcal {R} \left( x, y ; \left\{ \overline{\varvec{U}}_{k,l} \right\} _{(k,l) \in S_{i,j}} \right) , \end{aligned}$$

where $S_{i,j}$ denotes the stencil of the reconstruction for cell $\varOmega _{i,j}$. Many such reconstruction procedures have been developed in the literature and we refer to the references previously mentioned in Sect. 2.2.1. For example, the stencil for a spatially first-order accurate piecewise constant consists only of the cell itself $S_{i,j} = \left\{ \overline{\varvec{U}}_{i,j} \right\}$. For a spatially second-order accurate piecewise linear reconstruction, the stencil includes the four adjacent cells $S_{i,j} = \left\{ \overline{\varvec{U}}_{i ,j } ,\overline{\varvec{U}}_{i-1,j } ,\overline{\varvec{U}}_{i+1,j } ,\overline{\varvec{U}}_{i ,j-1} ,\overline{\varvec{U}}_{i ,j+1} \right\}$.

*Numerical fluxes* The numerical fluxes through the cell faces are obtained by numerical integration of one-dimensional numerical fluxes. The facially averaged numerical fluxes in *x*-direction are given by

100 $$\begin{aligned} \begin{aligned} \varvec{F}_{i+1/2,j}&= \frac{1}{\varDelta y} {\mathcal {Q}}_{i+1/2,j} \left( \mathcal {F}\left( \varvec{U}_{i+1/2-,j},\varvec{U}_{i+1/2+,j} \right) \right) \\&= \frac{1}{\varDelta y} \sum _{\beta =1}^{N_{q}} \omega _{\beta } ~ \mathcal {F} \left( \varvec{U}_{i ,j}\left( x_{i+1/2},y_{j,\beta } \right) , \varvec{U}_{i+1,j}\left( x_{i+1/2},y_{j,\beta } \right) \right) , \end{aligned} \end{aligned}$$

where ${\mathcal {Q}}_{i+1/2,j}$ denotes a *q*-th order accurate quadrature rule with $N_{q}$ nodes $y_{j,\beta } \in \varOmega _{j}$ and weights $\omega _{\beta }$, and $\mathcal {F}$ is a one-dimensional numerical flux formula in *x*-direction (see Sect. 2.2.2). Likewise, the facially averaged numerical fluxes in *y*-direction are given by

101 $$\begin{aligned} \begin{aligned} \varvec{G}_{i,j+1/2}&= \frac{1}{\varDelta x} {\mathcal {Q}}_{i,j+1/2} \left( \mathcal {G}\left( \varvec{U}_{i,j+1/2-},\varvec{U}_{i,j+1/2+} \right) \right) \\&= \frac{1}{\varDelta x} \sum _{\alpha =1}^{N_{q}} \omega _{\alpha } ~ \mathcal {G} \left( \varvec{U}_{i,j }\left( x_{i,\alpha },y_{j+1/2} \right) , \varvec{U}_{i,j+1}\left( x_{i,\alpha },y_{j+1/2} \right) \right) , \end{aligned} \end{aligned}$$

where ${\mathcal {Q}}_{i,j+1/2}$ denotes a *q*-th order accurate quadrature rule with $N_{q}$ nodes $x_{i,\alpha } \in \varOmega _{i}$ and weights $\omega _{\alpha }$, and $\mathcal {G}$ is a one-dimensional numerical flux formula in *y*-direction. In general, the numerical flux formulas in respective direction are often obtained by appropriate rotation of a one-dimensional flux formula in *x*-direction (see, e.g., Toro 2009 for details).

*Numerical source terms* We shall consider only unsplit methods for the numerical integration of the source terms. The cell-averaged numerical source terms are then given by

102 $$\begin{aligned} \begin{aligned} \overline{\varvec{S}}_{i,j}&= \frac{1}{\left| \varOmega _{i,j} \right| } {\mathcal {Q}}_{i,j} \left( \varvec{s}(\varvec{U}_{i,j}) \right) \\&= \frac{1}{\left| \varOmega _{i,j} \right| } \sum _{\alpha =1}^{N_{q}} \sum _{\beta =1 }^{N_{q}} \varvec{s}(\varvec{U}_{i,j}(x_{i,\alpha },y_{j,\beta })) , \end{aligned} \end{aligned}$$

where ${\mathcal {Q}}_{i,j}$ denotes a *q*-th order accurate quadrature rule with $N_{q} \times N_{q}$ nodes $x_{i,\alpha } \in \varOmega _{i}$, $y_{j,\beta } \in \varOmega _{j}$ and weights $\omega _{\alpha }$, $\omega _{\beta }$ in *x*- and *y*-direction, respectively. If we assume that the point values of the source term can be evaluated with spatial order of accuracy *s*, then this gives a spatially $\min (q,s)$-th order discretization of the source term (provided enough smoothness).

In practice, the same quadrature rules are often used in the numerical flux integration along the *x*- and *y*-direction. A tensor product quadrature rule is typically used for the numerical source term integration. For first- and second-order accuracy, the midpoint rule is the quadrature rule of choice. Beyond second-order accuracy, $N_{q}$-point Gauss-Legendre or Gauss-Lobatto quadrature rules are usually used ($N_{q} > 1$).

*Time discretization* The semi-discrete evolution Eq. (95) can be integrated with $\tau$-th order in time as in the one-dimensional case (see Sect. 2.2.4).

This concludes the brief description of a standard $\min (q,r,s,\tau )$-order accurate finite volume scheme in several space dimensions (for smooth enough solutions). We refer again to the excellent textbooks in the literature for precise derivations and generalizations (curvilinear coordinates, unstructured meshes, etc.).

#### Steady states

Balance laws in several space dimensions too admit non-trivial steady states. As in the one-dimensional case, the numerical approximation of solutions near a steady state characterized by a delicate balance is generally challenging for standard finite volume schemes. The underlying reason is again twofold. First, standard reconstruction procedures are not well suited to represent steady-state solutions and, second, the source term discretization is performed independently from the discrete flux divergence. Both conspire that steady states are only preserved up to truncation errors. Hence, the numerical resolution needs to be high enough over the entire simulation duration such that the physical phenomena of interest are not affected by these truncation errors. The required resolution in several dimensions may then quickly lead to prohibitively high computational costs.

Although truncation errors are at the very essence of numerical approximation, it is again highly desirable to design schemes that preserve exactly a discrete form of the steady states; even more so than in one dimension. The one-dimensional recipe based on piecewise steady reconstruction naturally extends to designing well-balanced schemes in multiple dimensions. The principle is again to decompose the solution into equilibrium and (not necessarily small) perturbation parts

103 $$\begin{aligned} \varvec{u}(x,y) = \varvec{u}_{eq}(x,y) + \varvec{\delta u}(x,y) , \end{aligned}$$

where the equilibrium part $\varvec{u}_{eq}(x,y)$ fulfills the steady state balance

104 $$\begin{aligned} \nabla \cdot {\textbf {\textsf {f}}}(\varvec{u}_{eq}) = \frac{\partial }{\partial x}\varvec{f}(\varvec{u}_{eq}) + \frac{\partial }{\partial y}\varvec{g}(\varvec{u}_{eq}) = \varvec{s}(\varvec{u}_{eq}) . \end{aligned}$$

As before, the piecewise steady reconstruction requires the computability of such multi-dimensional steady states. In general, this is an even more difficult undertaking than in one dimension. However, we again tacitly assume that the differential equation Eq. (104) can be solved for certain *steady states of interest*,

105 $$\begin{aligned} \varvec{U}_{eq}(x,y) = \varvec{u}_{eq}(x,y) + \mathcal {O}(h^{\epsilon }) , \end{aligned}$$

either exactly ($\epsilon = \infty$) or approximately ($\epsilon < \infty$). We reiterate that this is the main challenge when designing a well-balanced finite volume method for a particular system of balance laws. Some concrete examples are given in Sect. 3 for the Euler equations. Taking the above assumption for granted, the construction of a well-balanced finite volume scheme follows the same recipe as in one dimension. The following subsections describe the ingredients in detail.

Before we proceed, let us stress once more that the following developments crucially hinge on the (exact or approximate) solvability for the multi-dimensional steady states of interest. We will see some examples for the Euler equations, where this can be achieved for barotropic fluids. In general, however, this is far from obvious. Let us mention here the truly two-dimensional well-balanced schemes developed by Bianchini and Gosse (2018), Gosse and Vauchelet (2020), Gosse (2021) for several balance laws. The latter construct fully two-dimensional steady states profiles by solving elliptic boundary-value problems and may serve as a guidance for the type of equations of interest in computational astrophysics.

#### Piecewise steady reconstruction ${\mathcal{WR}}$

The piecewise steady reconstruction ${\mathcal{WR}}$ of a subcell profile $\varvec{U}_{i,j}(x)$ within each cell $\varOmega _{i,j}$ from the associated cell averages $\overline{\varvec{U}}_{i,j}$ is given by the decomposition

106 $$\begin{aligned} \varvec{U}_{i,j}(x,y) = {\mathcal{WR}} \left( x, y; \left\{ \overline{\varvec{U}}_{k,l} \right\} _{(k,l) \in S_{i,j}} \right) = \varvec{U}_{eq,i,j}(x,y) + \delta \varvec{U}_{i,j}(x,y) , \end{aligned}$$

where $\varvec{U}_{eq,i,j}(x,y)$ and $\delta \varvec{U}_{i,j}(x,y)$ denote the local equilibrium and perturbation part in cell $\varOmega _{i,j}$, respectively. Next, we present the construction of each part.

Within each cell $\varOmega _{i,j}$, the local equilibrium reconstruction $\varvec{U}_{eq,i,j}(x,y)$ is found by fitting an equilibrium solution $\varvec{U}_{eq}(x,y)$ among the steady states of interest to the cell average $\overline{\varvec{U}}_{i,j}$. Because the given cell average $\overline{\varvec{U}}_{i,j}$ may be arbitrarily far from an equilibrium, we first need to project $\overline{\varvec{U}}_{i,j}$ onto a cell average $\overline{\varvec{U}}_{eq,i,j}$ that is consistent with the steady states of interest. The local equilibrium reconstruction $\varvec{U}_{eq,i,j}(x,y)$ in cell $\varOmega _{i,j}$ is then determined by matching an equilibrium profile Eq. (105) with the cell average of the equilibrium projected conserved variables $\overline{\varvec{U}}_{eq,i,j}$

107 $$\begin{aligned} \frac{1}{\left| \varOmega _{i,j} \right| } {\mathcal {Q}}_{i,j} \left( \varvec{U}_{eq,i,j} \right) = \overline{\varvec{U}}_{eq,i,j} , \end{aligned}$$

where ${\mathcal {Q}}_{i,j}$ denotes a *q*-th order accurate quadrature rule over cell $\varOmega _{i,j}$. Within every cell, we now have a $\min (\epsilon ,q)$-th order accurate local equilibrium profile (if exact integration is chosen, then $q=\infty$). As in the one-dimensional case, the difficulty of this step depends strongly on the considered balance law and associated steady states of interest. Moreover, the possibility that no equilibrium profile is found needs to be considered too. In that case, it is again simply set to zero $\varvec{U}_{eq,i,j} = 0$.

The local equilibrium perturbation $\delta \varvec{U}_{i,j}(x,y)$ is obtained by extrapolating the local equilibrium profile $\varvec{U}_{eq,i,j}$ from cell $\varOmega _{i,j}$ to the neighboring cells, computing the cell average of this extrapolated equilibrium profile, and applying a standard piecewise polynomial reconstruction to the cell-averaged equilibrium perturbations:

108 $$\begin{aligned} \varvec{\delta U}_{i,j}(x,y) = \mathcal {R} \left( x, y ; \left\{ \overline{\varvec{U}}_{k,l} - \frac{1}{\left| \varOmega _{k,l} \right| } {\mathcal {Q}}_{k,l}(\varvec{U}_{eq,i,j}) \right\} _{(k,l) \in S_{i,j}} \right) . \end{aligned}$$

Intuitively, this senses how far away the states in the neighboring cells are from the equilibrium $\varvec{U}_{eq,i,j}$ in cell $\varOmega _{i,j}$. It is clear that the piecewise steady reconstruction preserves the equilibrium by construction, since the equilibrium perturbation vanishes $\varvec{\delta U}_{i,j} \equiv 0$, and it is $\min (\epsilon ,q,r)$-th order accurate at and away from equilibrium (for sufficiently smooth solutions). Likewise, it is obvious that the piecewise steady reconstruction reduces to a standard reconstruction Eq. (99) if $\varvec{U}_{eq,i,j} \equiv 0$ (e.g., if no local equilibrium is found in Eq. (107)).

The alternative formulations of the piecewise steady reconstruction Eqs. (49) and (51) can also be extended to several space dimensions in a straightforward manner. However, we shall leave this to the interested reader.

#### Well-balanced source term discretization $\mathcal{WS}\mathcal{}$

As in the one-dimensional setting, a direct discretization of the source terms as in Eq. (102) will in general not lead to a well-balanced scheme. In lieu thereof, we rewrite the source terms as

109 $$\begin{aligned} \begin{aligned} \varvec{s}(\varvec{U}_{i,j})&= \varvec{s}(\varvec{U}_{eq,i,j}) + \varvec{s}(\varvec{U}_{i,j}) - \varvec{s}(\varvec{U}_{eq,i,j}) \\&= - \frac{\partial }{\partial x}\varvec{f}(\varvec{U}_{eq,i,j}) - \frac{\partial }{\partial y}\varvec{g}(\varvec{U}_{eq,i,j}) + \varvec{s}(\varvec{U}_{i,j}) - \varvec{s}(\varvec{U}_{eq,i,j}) \end{aligned} \end{aligned}$$

by trivially adding and subtracting the local equilibrium component and replacing the latter by the equilibrium flux divergence. The well-balanced source term discretization is then obtained by numerical quadrature as follows

110 $$\begin{aligned} \begin{aligned} \overline{\varvec{S}}_{i,j} =&- \frac{1}{\varDelta x} \left( {\mathcal {Q}}_{i+1/2,j}(\varvec{f}(\varvec{U}_{eq,i,j})) - {\mathcal {Q}}_{i-1/2,j}(\varvec{f}(\varvec{U}_{eq,i,j})) \right) \\&- \frac{1}{\varDelta y} \left( {\mathcal {Q}}_{i,j+1/2}(\varvec{g}(\varvec{U}_{eq,i,j})) - {\mathcal {Q}}_{i,j-1/2}(\varvec{g}(\varvec{U}_{eq,i,j})) \right) \\&+ {\mathcal {Q}}_{i,j}( \varvec{s}(\varvec{U}_{i,j}) - \varvec{s}(\varvec{U}_{eq,i,j})) , \end{aligned} \end{aligned}$$

where the divergence theorem is applied to the equilibrium fluxes by numerical integration over the cell boundary. This is clearly a $\min (\epsilon ,q,r,s)$-th order accurate discretization of the source term. Moreover, note that the above well-balanced source term discretization seamlessly reduces to a standard discretization if no local equilibrium is found in the piecewise steady reconstruction (i.e., $\varvec{U}_{eq,i,j} \equiv 0$).

The alternative well-balanced source term discretization Eq. (60) and its high-order extension via Richardson extrapolation Eq. (62) can also be generalized to multiple space dimensions. We again leave this to the interested reader.

#### Assembling a well-balanced finite volume scheme

A well-balanced finite volume scheme Eq. (95) for the two-dimensional conservation law Eq. (94) is now readily assembled with the previously described ingredients:

1. A $\min (\epsilon ,q,r)$-th order accurate piecewise steady reconstruction ${\mathcal{WR}}$ (Eq. (106)).
2. Consistent and Lipschitz continuous numerical flux functions $\mathcal {F}$ and $\mathcal {G}$ in *x*- and *y*-direction, respectively.
3. An unsplit $\min (\epsilon ,q,r,s)$-th order accurate well-balanced source term discretization $\mathcal{WS}\mathcal{}$ (Eq. (110)).
4. A $\tau$-th order accurate time integrator $\mathcal {T}$.

The resulting scheme is a $\min {(\epsilon ,q,r,s,\tau )}$-th order accurate well-balanced finite volume scheme for balance laws in two dimensions (for smooth enough solutions). The scheme preserves exactly a $\min {(\epsilon ,q)}$-th order accurate discrete form of the steady states of interest (up to machine precision). The proof follows the same steps as in the one-dimensional case and is not repeated here.

Although we have focussed on the structured two-dimensional Cartesian case, well-balanced schemes can be designed for three-dimensional Cartesian and curvilinear meshes following the same recipe. The same applies to unstructured meshes (see, e.g., Grosheintz-Laval 2021).

### Well-balanced finite difference schemes

In this subsection, we focus on the construction of well-balanced finite difference schemes. Before we begin, we concisely summarize a generic finite difference scheme for one-dimensional balance laws. We refer to the excellent available textbooks and review articles for more comprehensive presentations, e.g., Laney (1998), Shu (1998, 2009). Equipped with the basic principles and accompanying notation, we then present a (non-exhaustive) selection of frameworks for well-balancing finite difference schemes. Further frameworks are developed, for instance, by Bermudez and Vazquez (1994), Gascón and Corberán (2001), Vukovic and Sopta (2002), Črnjarić-Žic et al. (2006), Caselles et al. (2009), Li et al. (2020), Li and Gao (2021) and we refer the interested reader to these references.

#### Conservative finite difference schemes

A semi-discrete conservative finite difference method approximates the differential form of the balance law Eq. (4) directly by

111 $$\begin{aligned} \frac{{\mathrm {d}}\varvec{U}_{i}}{{\mathrm {d}}t} = {\mathcal {L}}(\varvec{U})_{i} = - \frac{1}{\varDelta x} \left( \varvec{F}_{i+1/2} - \varvec{F}_{i-1/2} \right) + \varvec{S}_{i} , \end{aligned}$$

where the primal unknowns are the point values $\varvec{U}_{i}$ of the conserved variables at cell centers $x_{i}$. They approximate the point value of the exact solution $\varvec{u}(x, t)$ at cell centers:

112 $$\begin{aligned} \varvec{U}_{i}(t) \approx \varvec{u}(x_{i}, t) . \end{aligned}$$

The $\varvec{F}_{i\pm 1/2}$ and $\varvec{S}_{i}$ denote the numerical fluxes through the cell interfaces and the numerical source term at cell centers, respectively. We briefly describe these components in the following paragraphs. Since we consider semi-discrete schemes, we will drop the time dependence for ease of presentation.

*Flux reconstruction* The numerical fluxes at cell interfaces $\varvec{F}_{i\pm 1/2}$ are assumed to be consistent with the physical flux function $\varvec{f}$ and Lipschitz continuous. The flux difference in Eq. (111) is constructed to be an (*r*-th order) accurate approximation of the flux divergence in Eq. (4) at cell centers:

113 $$\begin{aligned} \frac{1}{\varDelta x} \left( \varvec{F}_{i+1/2} - \varvec{F}_{i-1/2} \right) = \left. \frac{\partial \varvec{f}}{\partial x} \right| _{x = x_{i}} + \mathcal {O}(\varDelta x^{r}) . \end{aligned}$$

The numerical fluxes $\varvec{F}_{i\pm 1/2}$ approximate the cell interface values $\varvec{h}(x_{i\pm 1/2})$ of a certain function $\varvec{h}(x)$ implicitly defined by

114 $$\begin{aligned} \varvec{f}(\varvec{u}(x)) = \frac{1}{\varDelta x} \int _{x - \frac{\varDelta x}{2}}^{x + \frac{\varDelta x}{2}} \varvec{h}(\xi ) {\mathrm {d}}\xi . \end{aligned}$$

The above definition implies that the cell averages of this function $\varvec{h}(x)$ are given by

115 $$\begin{aligned} \overline{\varvec{h}}_{i} = \frac{1}{\varDelta x} \int _{x_{i} - \frac{\varDelta x}{2}}^{x_{i} + \frac{\varDelta x}{2}} \varvec{h}(\xi ) {\mathrm {d}}\xi = \varvec{f}(\varvec{u}(x_{i})) . \end{aligned}$$

So the approximation of $\varvec{h}(x)$ boils down to a reconstruction problem. The same reconstruction procedures $\mathcal {R}$ as in finite volume methods can be used. Hence, an *r*-th order accurate approximation of $\varvec{h}(x_{i\pm 1/2})$ is readily computed:

116 $$\begin{aligned} \varvec{F}_{i\pm 1/2} = \mathcal {R} \left( x_{i\pm 1/2}; \{\varvec{f}(\varvec{U}_{k})\}_{k \in S_{i}} \right) = \varvec{h}(x_{i\pm 1/2}) + \mathcal {O}(\varDelta x^{r}) . \end{aligned}$$

One can show that the discrete flux divergence Eq. (113) is then an *r*-th order accurate approximation of the flux divergence at cell centers. Note that this may seem surprising as Eq. (113) looks like a simple second-order centered finite difference approximation (ensuring the crucial conservative character of the scheme). Unfortunately, beyond second-order accuracy ($r > 2$), this accuracy claim is only valid for uniform or smooth grids (see, e.g., Merriman 2003; Shu 2009).

For the subsequent developments, we have to slightly refine the so far introduced notation for the reconstruction procedures in Sect. 2.2.1. The reconstruction of some quantity *Q* within cell $$\varOmega _{i}$$ from cell averages $$\{\overline{Q}_{k}\}$$ over the cell’s stencil $$ S_{i}$$ can be written as

117 $$\begin{aligned} \mathcal {R} \left( x; \{\overline{Q}_{k}\}_{k \in S_{i}} \right) = \sum _{l \in S_{i}} c_{i,l} \left( x; \{\overline{Q}_{k}\}_{k \in S_{i}} \right) ~ \overline{Q}_{l} , \end{aligned}$$

where the $c_{i,l}$ denote the reconstruction coefficients. In general, these depend on the evaluation location $x \in \varOmega _{i}$ and on certain so-called smoothness measures of the reconstruction data $\{\overline{Q}_{k}\}_{k \in S_{i}}$ to guarantee non-oscillatory behavior. For smooth data, these coefficients tend towards optimal constants that maximize the accuracy of the reconstruction. Instead of using the data of the quantity to reconstruct, the smoothness measures can be based on the cell averages of some other quantity^11^$\{\overline{W}_{k}\}$

118 $$\begin{aligned} \mathcal {R} \left( x; \{(\overline{Q}_{k}, \overline{W}_{k})\}_{k \in S_{i}} \right) = \sum _{l \in S_{i}} c_{i,l} \left( x; \{\overline{W}_{k}\}_{k \in S_{i}} \right) ~ \overline{Q}_{l} , \end{aligned}$$

where the second argument of the reconstruction procedure is now a set of couples $\{(\overline{Q}_{k},\overline{W}_{k})\}_{k \in S_{i}}$. The first element is the cell average of the quantity to reconstruct and the second element is the cell average of the quantity on which the smoothness measures are based.

For finite difference schemes, a straightforward component-wise flux reconstruction for (systems of) balance laws may also lead to spurious oscillations, especially at high orders of accuracy and when strong flow discontinuities interact. Then a reconstruction in local characteristic variables as described at the end of Sect. 2.2.1 can be advantageous (see, e.g., Jiang and Shu 1996; Balsara and Shu 2000; Qiu and Shu 2002).

*Flux splitting* For numerical stability, upwinding is introduced by splitting the flux into right($+$) and left(−) propagating contributions

119 $$\begin{aligned} \varvec{f}(\varvec{u}) = \varvec{f}^{+}(\varvec{u}) + \varvec{f}^{-}(\varvec{u}) . \end{aligned}$$

The characteristic values of $\frac{\partial \varvec{f}^{+}}{\partial \varvec{u}}$ are all non-negative and the characteristic values of $\frac{\partial \varvec{f}^{-}}{\partial \varvec{u}}$ are all non-positive, i.e.,

120 $$\begin{aligned} \frac{\partial \varvec{f}^{+}}{\partial \varvec{u}} \ge 0 \quad {\text {and}} \quad \frac{\partial \varvec{f}^{-}}{\partial \varvec{u}} \le 0 . \end{aligned}$$

A popular flux splitting is the (local) Lax-Friedrichs flux splitting

121 $$\begin{aligned} \varvec{f}^{\pm }(\varvec{u}) = \frac{1}{2} \left( \varvec{f}(\varvec{u}) \pm \alpha \varvec{u} \right) \end{aligned}$$

with

122 $$\begin{aligned} \alpha = \max _{\varvec{u}} \left| \frac{\partial \varvec{f}}{\partial \varvec{u}}\right| . \end{aligned}$$

The fluxes can then be discretized according to the wave direction as

123a $$\begin{aligned} \varvec{F}^{+}_{i+1/2-}&= \mathcal {R} \left( x_{i+1/2}; \{\varvec{f}^{+}(\varvec{U}_{k})\}_{k \in S_{i}} \right) , \end{aligned}$$

123b $$\begin{aligned} \varvec{F}^{-}_{i+1/2+}&= \mathcal {R} \left( x_{i+1/2}; \{\varvec{f}^{-}(\varvec{U}_{k})\}_{k \in S_{i+1}} \right) . \end{aligned}$$

The right going flux contribution $\varvec{F}^{+}_{i+1/2-}$ is biased one cell to the left from the cell interface $x_{i+1/2}$ by using the reconstruction stencil $S_{i}$. Accordingly, the left going flux contribution $\varvec{F}^{-}_{i+1/2+}$ is biased one cell to the right from the cell interface $x_{i+1/2}$ by using the reconstruction stencil $S_{i+1}$. The complete upwinded numerical flux is then simply the sum of both contributions

124 $$\begin{aligned} \varvec{F}_{i+1/2} = \varvec{F}^{+}_{i+1/2-} + \varvec{F}^{-}_{i+1/2+} . \end{aligned}$$

The maximum in Eq. (122) can either be taken globally over the whole grid $\{\varvec{U}_{i}\}$ or locally, for example, over the flux reconstruction stencils $S_{i} \cup S_{i+1}$. Note that $\alpha$ can be interpreted as a parameter introducing artificial viscosity into the scheme, ensuring its numerical stability (see, e.g., Laney 1998).

*Numerical source term* The numerical source term $\varvec{S}_{i}$ is a point-value evaluated at cell centers such that it is an (*s*-th order) accurate approximation:

125 $$\begin{aligned} \varvec{S}_{i} = \varvec{s}(\varvec{U}_{i}) = \varvec{s}(\varvec{u}(x_{i})) + \mathcal {O}(\varDelta x^{s}) . \end{aligned}$$

*Time integration* The semi-discrete evolution equations Eq. (111) for the point values $\varvec{U}_{i}$ can be approximately integrated in time by $\tau$-th order accurate ODE solvers. Also for high-order finite difference schemes, the SSP Runge-Kutta methods are often used in practice. One popular example is the temporally third-order accurate SSP Runge-Kutta (SSP-RK3) method

126 $$\begin{aligned} \begin{aligned} \varvec{U}^{(1)}&= \varvec{U}^{n} + \varDelta t {\mathcal {L}}(\varvec{U}^{n}) , \\ \varvec{U}^{(2)}&= \frac{3}{4} \varvec{U}^{n} + \frac{1}{4} \left( \varvec{U}^{(1)} + \varDelta t {\mathcal {L}}(\varvec{U}^{(1)}) \right) , \\ \varvec{U}^{n+1}&= \frac{1}{3} \varvec{U}^{n} + \frac{2}{3} \left( \varvec{U}^{(2)} + \varDelta t {\mathcal {L}}(\varvec{U}^{(2)}) \right) , \end{aligned} \end{aligned}$$

where ${\mathcal {L}}$ denotes the spatial discretization operator in Eq. (111).

This completes the short description of a standard $\min (r,s,\tau )$-th order accurate finite difference scheme. The extension to several space dimensions is achieved by discretizing the flux divergence direction by direction. A significant computational advantage of finite difference schemes over finite volume schemes is that the multi-dimensional reconstruction procedures and quadrature rules are avoided entirely. The price of this advantage is the restriction to uniform or smooth curvilinear grids. We refer to the references given earlier for further details and developments. Next, we have a closer look at what happens near steady states.

#### Steady states

Standard finite difference schemes alike have troubles in preserving steady-state solutions in general. The fundamental cause of these troubles is analogous to finite volume methods: the source term discretization is independent of the discrete flux divergence. The consequence, in turn, is that steady states are generally preserved only up to truncation errors, requiring the numerical resolution to be high enough that they do not obscure the phenomena of interest.

The objective of well-balanced finite difference schemes is to preserve a discrete form of certain steady-state solutions. To do this, it is tacitly assumed, as for finite volume schemes, that the differential equation Eq. (44) can be solved (exactly or approximately) for the steady states of interest Eq. (45). The following subsections introduce a selection of frameworks for building well-balanced finite difference schemes for one-dimensional balance laws. Multi-dimensional balance laws can be treated by straightforwardly extending the one-dimensional building blocks.

#### Well-balanced finite difference schemes based on source term decomposition

A framework for the construction of high-order well-balanced finite difference schemes for a class of balance laws was developed by Xing and Shu (2005, 2006a, 2013), Li and Xing (2018b). The framework can also be applied to finite volume and discontinuous Galerkin methods (see, e.g., Xing and Shu 2006b; Noelle et al. 2009).

The framework assumes that each source term component can be (analytically) decomposed as follows

127 $$\begin{aligned} s_{m}(\varvec{u}, x) = a_{m} \left( \varvec{\nu }(\varvec{u},x), x \right) \frac{\partial }{\partial x} b_{m}(x) , \end{aligned}$$

where $s_{m}$ denotes the *m*-th component of the source term $\varvec{s}(\varvec{u},x)$.^12^ The functions $b_{m} = b_{m}(x)$ are supposed to depend only on space and the $a_{m} = a_{m} \left( \varvec{\nu }, x \right)$ depend additionally on so-called *equilibrium variables* denoted by $\varvec{\nu }$. The latter are function of the conserved variables and space, i.e., $\varvec{\nu } = \varvec{\nu }(\varvec{u}, x)$, and they are required to become constant at the steady states of interest $\varvec{U}_{eq}$

128 $$\begin{aligned} \varvec{\nu }(\varvec{U}_{eq}, x) = {\text {const.}}, \end{aligned}$$

explaining the name equilibrium variables. Similarly, the functions $a_{m}$ are assumed to become spatially constant for $\varvec{U}_{eq}$:

129 $$\begin{aligned} a_{m}(\varvec{\nu }(\varvec{U}_{eq}, x), x) = {\text {const.}}\end{aligned}$$

As a result, such a source term decomposition clearly has the following property at a steady state of interest $\varvec{U}_{eq}$:

$$\begin{aligned} \begin{aligned} \frac{\partial }{\partial x} f_{m}(\varvec{U}_{eq})&= s_{m}(\varvec{U}_{eq}, x) \\&\overset{\mathrm {Eq. }(127)}{=} a_{m} \left( \varvec{\nu }(\varvec{U}_{eq}, x), x \right) \frac{\partial }{\partial x} b_{m}(x) \\&\overset{\mathrm {Eq. }(129)}{=} \frac{\partial }{\partial x} \left( a_{m} \left( \varvec{\nu }(\varvec{U}_{eq}, x), x \right) b_{m}(x) \right) , \end{aligned} \end{aligned}$$

which obviously implies that

130 $$\begin{aligned} f_{m}(\varvec{U}_{eq}) - a_{m} \left( \varvec{\nu }(\varvec{U}_{eq}, x), x \right) b_{m}(x) = {\text {const.}}\end{aligned}$$

Although these assumptions may seem rather restrictive, a broad class of balance laws and associated steady states possess such a source term decomposition. We will provide some illustrating examples below together with a general recipe.

The key idea is now to mimic the above property at the discrete level to ensure the exact balance at steady states. We begin by discussing the case without flux splitting. Then, this is achieved by approximating the derivative of the function $b_{m}$ with the same finite difference operator as used for the approximation of the flux divergence Eq. (113)

131 $$\begin{aligned} \left. \frac{\partial }{\partial x}b_{m}(x) \right| _{x = x_{i}} = \frac{1}{\varDelta x} \left( b_{m,i+1/2} - b_{m,i-1/2} \right) + \mathcal {O}(\varDelta x^{r}) , \end{aligned}$$

with

132 $$\begin{aligned} b_{m,i\pm 1/2} = \mathcal {R} \left( x_{i\pm 1/2}; \{(b_{m}(x_{k}),f_{m}(\varvec{U}_{k}))\}_{k \in S_{i}} \right) . \end{aligned}$$

Note that the above reconstruction uses the *m*-th component of the flux $f_{m}$ as smoothness measures. This ensures the consistency of the difference operators applied to the flux and source term by construction.^13^ An *r*-th order accurate discretization of the *m*-th source term component is thus given by

133 $$\begin{aligned} S_{m,i} = a_{m}(\varvec{\nu }(\varvec{U}_{i},x_{i}), x_{i}) ~ \frac{b_{m,i+1/2} - b_{m,i-1/2}}{\varDelta x} \end{aligned}$$

and the complete source term vector is simply $\varvec{S}_{i} = [\cdots , S_{m,i}, \cdots ]^{T}$.

Before we discuss the case with flux splitting, we verify that the scheme Eq. (111) and the above source term discretization Eq. (133) is well-balanced. Suppose that the conserved variables point values $\varvec{U}_{i}$ are initialized with an equilibrium $\varvec{U}_{eq}(x)$ fulfilling all the necessary assumptions, that is, $\varvec{U}_{i} = \varvec{U}_{eq}(x_{i})$. Then Eq. (129) gives immediately at all cell centers $x_{i}$

$$\begin{aligned} a_{m}(\varvec{\nu }(\varvec{U}_{i},x_{i}), x_{i}) = a_{m} = {\text {const.}}\end{aligned}$$

and one verifies that Eq. (130) holds discretely as follows

$$\begin{aligned}&F_{m,i\pm 1/2} - a_{m} ~ b_{m,i\pm 1/2} \\&\quad = \mathcal {R} \left( x_{i\pm 1/2}; \{f_{m}(\varvec{U}_{k})\}_{k \in S_{i}} \right) - a_{m} \mathcal {R} \left( x_{i\pm 1/2}; \{(b_{m}(x_{k}),f_{m}(\varvec{U}_{k}))\}_{k \in S_{i}} \right) \\&\quad = \sum _{l \in S_{i}} c_{i,l} \left( x_{i\pm 1/2}; \{f_{m}(\varvec{U}_{k})\}_{k \in S_{i}} \right) \underbrace{ \left( f_{m}(\varvec{U}_{l}) - a_{m} ~ b_{m}(x_{l}) \right) }_{= C = {\text {const.}}} \\&\quad = C . \end{aligned}$$

In the last equality, the consistency of the reconstruction was used (constant functions are reconstructed exactly). Now the semi-discrete update equation Eq. (111) gives for the *m*-th solution component $U_{m,i}$

$$\begin{aligned} \frac{{\mathrm {d}}U_{m,i}}{{\mathrm {d}}t}&= - \frac{1}{\varDelta x} \left( F_{m,i+1/2} - F_{m,i-1/2} \right) + S_{m,i} \\&= - \frac{1}{\varDelta x} \left( F_{m,i+1/2} - F_{m,i-1/2} \right) + a_{m} \frac{b_{m,i+1/2} - b_{m,i-1/2}}{\varDelta x} \\&= - \frac{1}{\varDelta x} \left( F_{m,i+1/2} - a_{m} b_{m,i+1/2} - F_{m,i-1/2} + a_{m} b_{m,i-1/2} \right) \\&= - \frac{1}{\varDelta x} \left( C - C \right) = 0 . \end{aligned}$$

Since this is valid for all the solution components, we conclude that the scheme is well-balanced as claimed.

Next, we discuss the case with flux splitting Eq. (119) and Eq. (123). Then, the source term is split into a right ($+$) and a left (−) contribution as follows

134 $$\begin{aligned} b_{m,i+1/2} = \frac{1}{2} \left( b^{+}_{m,i+1/2-} + b^{-}_{m,i+1/2+} \right) \end{aligned}$$

with

135 $$\begin{aligned} \begin{aligned} b^{+}_{m,i+1/2-}&= \mathcal {R} \left( x_{i+1/2}; \{(b_{m}(x_{k}),f_{m}^{+}(\varvec{U}_{k}))\}_{k \in S_{i}} \right) , \\ b^{-}_{m,i+1/2+}&= \mathcal {R} \left( x_{i+1/2}; \{(b_{m}(x_{k}),f_{m}^{-}(\varvec{U}_{k}))\}_{k \in S_{i+1}} \right) . \end{aligned} \end{aligned}$$

Note that the above reconstruction uses the *m*-th component of the left($+$)/right(−) propagating flux contributions $f_{m}^{\pm }$ as smoothness measures. This ensures the consistency of the difference operators applied to the flux and source term by construction.

To guarantee an exact balance, the flux splitting Eq. (121) also needs to be slightly modified. One possibility suggested by Xing and Shu (2006a) is the following:

136 $$\begin{aligned} f_{m}^{\pm }(\varvec{u}) = \frac{1}{2} \left( f_{m}(\varvec{u}) \pm {\widetilde{\alpha }}_{m} a_{m}(\varvec{\nu }(\varvec{u},x), x) \right) . \end{aligned}$$

Due to Eq. (129), this ensures that at steady state the artificial viscosity tends towards zero. Importantly, this does not interfere with the scheme’s design accuracy (see Xing and Shu 2006a). However, the constant ${\widetilde{\alpha }}_{m}$ should be suitably adjusted to maintain enough artificial viscosity for numerical stability. This depends on the concrete application and may require some fine-tuning. The well-balancing property with flux splitting can now be shown easily as before.

This ends the description of the well-balanced finite difference schemes based on source term decomposition. To conclude on this family of schemes, let us give two illustrating examples of balance laws with their corresponding equilibrium variables and source term decomposition.

*Example: Linear advection–reaction equation* Consider (again) the linear advection–reaction equation

137 $$\begin{aligned} \frac{\partial u}{\partial t} + a \frac{\partial u}{\partial x} = - \lambda u \end{aligned}$$

which still has the steady-state solutions

138 $$\begin{aligned} U_{eq}(x) = C e^{- \lambda / a x} . \end{aligned}$$

A particular choice for the equilibrium variable of Eq. (137) is

139 $$\begin{aligned} \nu (u, x) = \log (u) + \frac{\lambda }{a} x , \end{aligned}$$

which becomes clearly a constant for the steady states $U_{eq}(x)$. A source term decomposition of the form Eq. (127) is then given by

140 $$\begin{aligned} s_{1}(u,x) = a_{1}(\nu ,x) \frac{\partial }{\partial x}b_{1}(x) \end{aligned}$$

with

141 $$\begin{aligned} a_{1}(\nu ,x) = e^{\nu } \quad {\text {and}} \quad b_{1}(x) = a e^{-\frac{\lambda }{a} x}. \end{aligned}$$

*Example: Euler equations* As a slightly more complex example, consider the one-dimensional Euler equations describing the motion of fluids subject to a gravitational field with potential $\phi$. They are given by the conservation of mass, momentum and fluid energy

142 $$\begin{aligned} \frac{\partial \varvec{u}}{\partial t} + \frac{\partial \varvec{f}}{\partial x} = \varvec{s} , \end{aligned}$$

where the vector of conserved variables, fluxes and source terms are

143 $$\begin{aligned} \varvec{u} = \begin{bmatrix} \rho \\ \rho v_{x} \\ E \end{bmatrix} , \; \varvec{f}(\varvec{u}) = \begin{bmatrix} \rho v_{x} \\ \rho v_{x}^{2} + p \\ (E + p) v_{x} \end{bmatrix} , \; \varvec{s}(\varvec{u}) = - \begin{bmatrix} 0 \\ \rho \\ \rho v_{x} \end{bmatrix} \frac{\partial \phi }{\partial x} . \end{aligned}$$

Here, $\rho$ is the mass density, $v_{x}$ the velocity and $E = \rho e + \frac{1}{2} \rho v_{x}^2$ the total fluid energy density. The pressure *p* is related to the density $\rho$ and specific internal energy *e* by an equation of state $p = p(\rho , e)$. For steady adiabatic flow, the equilibrium variables are given by (see, e.g., Landau and Lifshitz 1987)

144 $$\begin{aligned} \varvec{\nu }(\varvec{u},x) = \begin{bmatrix} s \\ \rho v_{x} \\ \frac{v_{x}^{2}}{2} + h + \phi \end{bmatrix} , \end{aligned}$$

where *h* and *s* denote the fluid’s specific enthalpy and entropy, respectively. Let $\varvec{U}_{eq}(x)$ denote the conserved variables corresponding to some given equilibrium variables $\varvec{\nu }_{eq}$, i.e., $\varvec{U}_{eq}(x) = \varvec{u}(\varvec{\nu }_{eq},x)$ is the inverse transformation of the equilibrium variables to conserved variables (see Grosheintz-Laval and Käppeli 2020 for a way to compute it). Also, let $p_{eq}(x)$ denote the corresponding equilibrium pressure. A source term decomposition of the form Eq. (127) is then given by the following. The first component is trivial

145 $$\begin{aligned} s_{1}(\varvec{u},x) = a_{1}(\varvec{\nu }(\varvec{u},x),x) \frac{\partial }{\partial x} b_{1}(x) \end{aligned}$$

with

146 $$\begin{aligned} a_{1}(\varvec{\nu },x) = 0 \quad {\text {and}} \quad b_{1}(x) = 0 . \end{aligned}$$

Li and Xing (2018b) suggest for the second component

147 $$\begin{aligned} s_{2}(\varvec{u},x) = a_{2}(\varvec{\nu }(\varvec{u},x),x) \frac{\partial }{\partial x} b_{2}(x) \end{aligned}$$

with

148 $$\begin{aligned} a_{2}(\varvec{\nu },x) = \frac{\rho (x)}{\rho _{eq}(x)} \quad {\text {and}} \quad b_{2}(x) = p_{eq}(x) . \end{aligned}$$

The third component is already in the desired form

149 $$\begin{aligned} s_{3}(\varvec{u},x) = a_{3}(\varvec{\nu }(\varvec{u},x),x) \frac{\partial }{\partial x} b_{3}(x) \end{aligned}$$

with

150 $$\begin{aligned} a_{3}(\varvec{\nu },x) = \rho v_{x}(x) \quad {\text {and}} \quad b_{3}(x) = \phi (x) . \end{aligned}$$

The previous two examples suggest the following general recipe for the functions $a_{m}$ and $b_{m}$

151 $$\begin{aligned} a_{m}(\varvec{\nu }(\varvec{u},x),x) = \frac{s_{m}(\varvec{u}, x)}{s_{m}(\varvec{u}(\varvec{\nu }_{eq},x), x)} \quad {\text {and}} \quad b_{m}(x) = f_{m}(\varvec{u}(\varvec{\nu }_{eq},x)) , \end{aligned}$$

where $\varvec{\nu }_{eq}$ are some fixed equilibrium variables. However, we shall not further discuss the above generic decomposition and refer the interested reader to the original research articles cited at the beginning of this section for the details.

#### Well-balanced finite difference schemes based on piecewise steady flux reconstruction

Parés and Parés-Pulido (2021) recently proposed a framework for designing high-order well-balanced finite difference schemes for balance laws. It is elegantly based on a piecewise steady flux reconstruction and, as such, is closely related to the finite volume recipe based on piecewise steady reconstruction presented in Sect. 2.4. Unfortunately, the formulation is generally not fully conservative in that if a system of balance laws possesses a conservative subsystem,^14^ the method may not reduce to a conservative finite difference method for that subsystem of conservation laws. However, the conservation errors are of the order of the truncation errors and vanish in the infinite resolution limit.

Since high-order finite difference schemes are based on flux reconstructions, the key idea is to decompose the flux into equilibrium and (not necessarily small) perturbation parts

152 $$\begin{aligned} \begin{aligned} \varvec{f}(\varvec{u}(x,t))&= \varvec{f}(\varvec{u}_{eq}(x)) + \left( \varvec{f}(\varvec{u}(x,t)) - \varvec{f}(\varvec{u}_{eq}(x)) \right) \\&= \varvec{f}(\varvec{u}_{eq}(x)) + \varvec{\delta f}(x,t) , \end{aligned} \end{aligned}$$

where the equilibrium part $\varvec{f}(\varvec{u}_{eq}(x))$ fulfills by definition the steady-state balance Eq. (44). Like in the piecewise steady reconstruction used in finite volume schemes, this requires the ability to compute such steady states of interest Eq. (45). Parés and Parés-Pulido (2021) then subtract the steady state Eq. (44) from the balance law Eq. (4),

153 $$\begin{aligned} \frac{\partial \varvec{u}}{\partial t}&= - \frac{\partial }{\partial x} \left( \varvec{f}(\varvec{u}(x,t)) - \varvec{f}(\varvec{u}_{eq}(x)) \right) + \varvec{s}(\varvec{u}(x,t)) - \varvec{s}(\varvec{u}_{eq}(x)) \nonumber \\&= - \frac{\partial }{\partial x} \varvec{\delta f}(x,t) + \varvec{\delta s}(x,t) , \end{aligned}$$

and suggest the following semi-discrete finite difference approximation

154 $$\begin{aligned} \frac{{\mathrm {d}}\varvec{U}_{i}}{{\mathrm {d}}t} = - \frac{1}{\varDelta x} \left( \varvec{\delta F}_{i,i+1/2} - \varvec{\delta F}_{i,i-1/2} \right) + \varvec{\delta S}_{i} . \end{aligned}$$

Next, we describe the Parés and Parés-Pulido (2021) well-balanced finite difference scheme in detail. This will especially clarify the double index notation for the numerical equilibrium perturbation fluxes $\varvec{\delta F}_{i,i\pm 1/2}$.

One begins with the determination of a local equilibrium profile $\varvec{U}_{eq,i}(x)$ in each cell $\varOmega _{i}$ by fitting an equilibrium solution $\varvec{U}_{eq}(x)$ among the steady states of interest to the point value $\varvec{U}_{i}$. Because $\varvec{U}_{i}$ may be far from a steady state, it is first projected onto a point value $\varvec{U}_{eq,i}$ that is consistent with the steady states of interest. Therewith, the local equilibrium profile $\varvec{U}_{eq,i}(x)$ in cell $\varOmega _{i}$ is determined by matching an equilibrium profile $\varvec{U}_{eq}(x)$ to the equilibrium projected point value $\varvec{U}_{eq,i}$

155 $$\begin{aligned} \frac{\partial }{\partial x} \varvec{f}(\varvec{U}_{eq,i}(x)) = \varvec{s}(\varvec{U}_{eq,i}(x)) \end{aligned}$$

such that the profile is anchored at the cell center point value $\varvec{U}_{eq,i}$

156 $$\begin{aligned} \varvec{U}_{eq,i}(x_{i}) = \varvec{U}_{eq,i} . \end{aligned}$$

Note that this is the main difference with the piecewise steady reconstruction, where the local equilibrium profile is matched with the cell average.

The equilibrium perturbation flux divergence $\varvec{\delta f}$ is discretized to *r*-th order as

157 $$\begin{aligned} \frac{1}{\varDelta x} \left( \varvec{\delta F}_{i,i+1/2} - \varvec{\delta F}_{i,i-1/2} \right) = \left. \frac{\partial }{\partial x} \varvec{\delta f}_{i}(x) \right| _{x = x_{i}} + \mathcal {O}(\varDelta x^{r}) , \end{aligned}$$

where the numerical equilibrium perturbation fluxes $\varvec{\delta F}_{i,i\pm 1/2}$ approximate the cell interface values $\varvec{\delta h}_{i}(x_{i\pm 1/2})$ of a certain function $\varvec{\delta h}_{i}(x)$ implicitly defined by

158 $$\begin{aligned} \varvec{\delta f}_{i}(x) = \frac{1}{\varDelta x} \int _{x - \frac{\varDelta x}{2}}^{x + \frac{\varDelta x}{2}} \varvec{\delta h}_{i}(\xi ) {\mathrm {d}}\xi . \end{aligned}$$

Following the same principles as in a standard finite difference scheme, the above definition implies that the cell averages of this function $\varvec{\delta h}_{i}(x)$ are given by

159 $$\begin{aligned} \overline{\varvec{\delta h}}_{i,k} = \frac{1}{\varDelta x} \int _{x_{k} - \frac{\varDelta x}{2}}^{x_{k} + \frac{\varDelta x}{2}} \varvec{\delta h}_{i}(\xi ) {\mathrm {d}}\xi = \varvec{f}(\varvec{u}(x_{k})) - \varvec{f}(\varvec{U}_{eq,i}(x_{k})) + \mathcal {O}(\varDelta x^{\epsilon }) . \end{aligned}$$

Now the double index notation becomes clear. The first index “*i*” indicates the cell where the equilibrium reconstruction $\varvec{U}_{eq,i}(x)$ is anchored. The second index “*k*” indicates the cell center $x_{k}$ where the equilibrium perturbation flux is evaluated. Also notice that the equilibrium reconstruction $\varvec{U}_{eq,i}(x)$ might only be an $\epsilon$-th order approximation of a true equilibrium $\varvec{u}_{eq}(x)$, which means that also the cell averages $\overline{\varvec{\delta h}}_{i,k}$ are only accurate to this precision. An *r*-th order reconstruction procedure is then applied to the cell averages $\overline{\varvec{\delta h}}_{i,k}$ to obtain the numerical equilibrium perturbation fluxes at cell interfaces

160 $$\begin{aligned} \varvec{\delta F}_{i,i\pm 1/2}&= \mathcal {R} \left( x_{i\pm 1/2}; \{ \varvec{f}(\varvec{U}_{k}) - \varvec{f}(\varvec{U}_{eq,i}(x_{k}))\}_{k \in S_{i}} \right) \nonumber \\&= \varvec{\delta h}_{i}(x_{i\pm 1/2}) + \mathcal {O}(\varDelta x^{\min (r,\epsilon )}) , \end{aligned}$$

For numerical stability, these numerical fluxes need to be upwinded and this will be explained below. Notice that two reconstructions, $\varvec{\delta F}_{i,i+1/2}$ and $\varvec{\delta F}_{i+1,i+1/2}$, have to be computed at every cell interface. In contrast, only one is needed in a standard finite difference scheme.

The numerical perturbation source term $\varvec{\delta S}_{i}$ is discretized as

161 $$\begin{aligned} \varvec{\delta S}_{i} = \varvec{s}(\varvec{U}_{i}) - \varvec{s}(\varvec{U}_{eq,i}(x_{i})) . \end{aligned}$$

Note that $\varvec{\delta S}_{i}$ does not vanish in general because the solution could be far away from a steady state.

Before we discuss the upwinding of the numerical equilibrium perturbation fluxes, let us verify that the scheme Eq. (154) is indeed well-balanced. Suppose that we initialize the conserved variables point values $\varvec{U}_{i}$ with a steady state of interest $\varvec{U}_{eq}(x)$

162 $$\begin{aligned} \varvec{U}_{i} = \varvec{U}_{eq}(x_{i}) , \end{aligned}$$

that is, we initialize with well-prepared initial data. The equilibrium projection $\varvec{U}_{eq,i} = \varvec{U}_{i}$ is trivial. Assuming that for all cells $\varOmega _{i}$ the local equilibrium reconstruction $\varvec{U}_{eq,i}(x)$ matches with $\varvec{U}_{eq}(x)$ at cell centers (i.e., $\varvec{U}_{eq,i}(x_{i}) = \varvec{U}_{eq}(x_{i})$), then the equilibrium perturbation flux vanishes at all cell centers (i.e., $\varvec{f}(\varvec{U}_{k}) - \varvec{f}(\varvec{U}_{eq,i}(x_{k})) = 0$). Any consistent reconstruction procedure will then produce vanishing numerical equilibrium perturbation fluxes at cell interfaces $\varvec{\delta F}_{i,i\pm 1/2}$ for all cells $\varOmega _{i}$. Along the same lines, the numerical source term perturbation Eq. (161) vanishes identically. Therefore, the semi-discrete finite difference scheme Eq. (154) gives

163 $$\begin{aligned} \frac{{\mathrm {d}}\varvec{U}_{i}}{{\mathrm {d}}t} = 0 \end{aligned}$$

and the scheme is well-balanced as advertised.

For numerical stability, the numerical equilibrium perturbation fluxes have to be properly upwinded. As in a standard finite difference scheme, this is achieved by flux splitting:

164a $$\begin{aligned} \varvec{\delta F}^{+}_{i,i+1/2-}&= \mathcal {R} \left( x_{i+1/2}; \{ \varvec{f}^{+}(\varvec{U}_{k}) - \varvec{f}^{+}(\varvec{U}_{eq,i}(x_{k})) \}_{k \in S_{i}} \right) , \end{aligned}$$

164b $$\begin{aligned} \varvec{\delta F}^{-}_{i,i+1/2+}&= \mathcal {R} \left( x_{i+1/2}; \{ \varvec{f}^{-}(\varvec{U}_{k}) - \varvec{f}^{-}(\varvec{U}_{eq,i}(x_{k})) \}_{k \in S_{i+1}} \right) . \end{aligned}$$

The complete upwind numerical equilibrium perturbation fluxes are then

165 $$\begin{aligned} \varvec{\delta F}_{i,i+1/2} = \varvec{\delta F}^{+}_{i,i+1/2-} + \varvec{\delta F}^{-}_{i,i+1/2+} . \end{aligned}$$

For example, the (local) Lax-Friedrichs flux splitting Eq. (121) could be used. It is straightforward to show that flux splitting does not interfere with the well-balancedness of the scheme.

This concludes the presentation of the high-order well-balanced finite difference schemes of Parés and Parés-Pulido (2021). We want to stress the fact that the numerical equilibrium perturbation fluxes at the cell interfaces are not equal in general:

$$\begin{aligned} \varvec{\delta F}_{i,i+1/2} \ne \varvec{\delta F}_{i+1,i+1/2} \end{aligned}$$

Of course, this is not unexpected for balance laws and their non-conservative character due to their source terms. However, the latter may also be the case for the conservative equations in a system of balance laws. Fortunately, one can show that the conservation errors made by the schemes are of the order of the truncation error and, therefore, tend to vanish with increasing resolution. Moreover, this non-conservation can be avoided for certain balance laws using some fine-tuning of the artificial viscosity terms in the numerical fluxes. We refer to Parés and Parés-Pulido (2021) for the intricate details.

## Well-balanced methods for the Euler equations

In this section, we showcase the frameworks introduced in Sect. 2. We consider the Euler equations of compressible hydrodynamics as a prototypical example for a system of balance laws. The section starts by introducing the equations and their steady states. This is followed by a classification of well-balanced schemes for the Euler equations and the presentation of some representative example for each class. The section concludes by presentating several numerical test problems highlithing the performance of the well-balanced schemes.

### The Euler equations

The Euler equations describe the motion of ideal fluids subject to (gravitational) forces in which thermal conductivity and viscosity are unimportant. They express the conservation of fluid mass, momentum and energy:

166a $$\begin{aligned} \frac{\partial \rho }{\partial t} + \nabla \cdot \left( \rho \varvec{v} \right)&= 0 , \end{aligned}$$

166b $$\begin{aligned} \frac{\partial \rho \varvec{v}}{\partial t} + \nabla \cdot \left( \varvec{v} \rho \varvec{v} \right) + \nabla p&= - \rho \nabla \phi , \end{aligned}$$

166c $$\begin{aligned} \frac{\partial E}{\partial t} + \nabla \cdot \left[ \left( E + p \right) \varvec{v} \right]&= - \rho \varvec{v} \cdot \nabla \phi . \end{aligned}$$

Here $\rho$ is the fluid mass density, $\varvec{v}$ the velocity and *p* the pressure. The total fluid energy $E = \rho e + \frac{\rho }{2} v^{2}$ is composed of internal and kinetic energy densities. The pressure *p* is related to the density $\rho$ and the specific internal energy *e* through an equation of state (EoS) $p = p(\rho ,e)$. The latter determines all thermodynamic quantities by specifying any two values describing the state.^15^

The source terms on the right-hand side of Eqs. (166) model the effect of gravitational forces on the fluid. They are characterized by a gravitational potential, which may either be a given function of the coordinates, $\phi = \phi (\varvec{x})$, or prescribed by the fluid’s self-gravity depending on the concrete application. In the latter case, the potential is determined by the Poisson equation,

167 $$\begin{aligned} \nabla ^2 \phi = 4 \pi G \rho , \end{aligned}$$

where *G* is the gravitational constant.

By including the gravitational interaction explicitly into the conservation balance, as opposed to a generic external force, the Euler equations can be written in alternative forms that emphasize their total conservative character. In the case of self-gravitating flows, the momentum source term can be reformulated as the divergence of the gravitational stress tensor, allowing its inclusion into the momentum flux tensor (see, e.g., Shu 1992). Unfortunately, when gravity is described by an external static gravitational potential, the source term in the momentum equations cannot be eliminated. However, the energy Eq. (166c) can be reformulated as

168 $$\begin{aligned} \frac{\partial E_{T}}{\partial t} + \nabla \cdot \left[ \left( E + p \right) \varvec{v} + \varvec{F}_{G} \right] = 0 \end{aligned}$$

in both cases. Here $E_{T} = E + E_{G}$ is the total (fluid and gravitational) energy density and $\varvec{F}_{G}$ represents a “gravitational energy flux”. For a static gravitational field, the gravitational energy density is $E_{G} = \rho \phi$. If the gravitational potential is prescribed by the fluid’s self-gravity, the gravitational energy density is $E_{G} = \rho \phi /2$, where the factor 1/2 avoids the double-counting of pairs of fluid elements. For time-independent gravitational fields, as is the case at steady states which are our primal concern, this energy flux component becomes $\varvec{F}_{G} = \rho \varvec{v} \phi$. Hence, the energy equation can be written in conservation form

169 $$\begin{aligned} \frac{\partial E_{T}}{\partial t} + \nabla \cdot \left[ \left( E_{T} + p \right) \varvec{v} \right] = 0 . \end{aligned}$$

Below, we will also use a slightly different form that evolves directly the fluid energy *E* with the gravity source term expressed as follows

170 $$\begin{aligned} \frac{\partial E}{\partial t} + \nabla \cdot \left[ \left( E + p \right) \varvec{v} \right] = - \nabla \cdot \left( \rho \varvec{v} \phi \right) + \phi ~ \nabla \cdot \rho \varvec{v} . \end{aligned}$$

For static gravitational fields, the source term can then be discretized such that the total energy $E_{T}$ is preserved in a straightforward manner. We refer to the developments in Springel (2010), Hanawa (2019), Katz et al. (2016), Mullen et al. (2021) for an in-depth discussion and, in particular, the extension to self-gravitating flows.

Before we proceed with a discussion of some interesting steady states of the Euler equations, let us rewrite them in canonical balance law form. For simplicity, we restrict the presentation to a one-dimensional setting. The Euler Eqs. (166) then compactly read

171 $$\begin{aligned} \frac{\partial \varvec{u}}{\partial t} + \frac{\partial \varvec{f}}{\partial x} = \varvec{s}, \end{aligned}$$

where the conserved variables, fluxes, and gravitational source term are given by

172 $$\begin{aligned} \varvec{u} = \begin{bmatrix} \rho \\ \rho v_{x} \\ E \end{bmatrix} , \; \varvec{f}(\varvec{u}) = \begin{bmatrix} \rho v_{x} \\ \rho v_{x}^{2} + p \\ (E + p) v_{x} \end{bmatrix} , \; \varvec{s}(\varvec{u}) = - \begin{bmatrix} 0 \\ \rho \\ \rho v_{x} \end{bmatrix} \frac{\partial \phi }{\partial x} . \end{aligned}$$

Moreover, the primitive variables are denoted by $\varvec{w} = [\rho , v_{x}, p]^{T}$.

### Steady states of the Euler equations

The Euler Eqs. (166) allow for a myriad of non-trivial steady states. Many interesting astrophysical applications occur near or involve such equilibrium flows. A particular example is hydrostatic equilibrium,

173 $$\begin{aligned} \nabla p = - \rho \nabla \phi , \end{aligned}$$

which describes the mechanical balance between pressure and gravity forces. In the case of self-gravity, the hydrostatic equation can be combined with the Poisson Eq. (167) to yield (Landau and Lifshitz 1987)

174 $$\begin{aligned} \nabla \cdot \left( \frac{\nabla p}{\rho } \right) = - \nabla ^2 \phi = - 4 \pi G \rho . \end{aligned}$$

It must be emphasized that the hydrostatic equilibrium equation only describes a mechanical equilibrium. A certain thermal stratification has to be supplemented to integrate the equations.

Another steady-state example of the Euler Eqs. (166) is provided by steady adiabatic flow. Such steady flows are governed by Bernoulli’s equation (Landau and Lifshitz 1987)

175 $$\begin{aligned} \frac{v^2}{2} + h + \phi = {\text {const.}}, \end{aligned}$$

where *h* is the specific enthalpy. The above relation holds along each streamline, i.e., lines tangent to the velocity of the flow. In general, the constant may take different values for different streamlines. Flows near such steady states are ubiquitous in nature as in accretion and wind phenomena.

However, hydrostatic equilibrium and steady adiabatic flow are just two particular examples. In many astrophysical applications, the flow of interest takes place near more complex equilibrium configurations. One example is rotational hydrostatic equilibrium which describes the balance between pressure, gravitational and centrifugal forces. Under certain conditions, such equilibrium configurations fulfill Eq. (173) with the gravitational potential $\phi$ replaced by an effective potential $\varPhi$ including effects of rotation

176 $$\begin{aligned} \varPhi (\varpi , z) = \phi (\varpi , z) - \frac{1}{2} \varOmega ^{2} \varpi ^{2} , \end{aligned}$$

where $\varpi$ is the cylindrical radius and $\varOmega$ (the constant) angular velocity (see, e.g., Tassoul 1978).

The Euler equations are an idealized model for the description of flows. They lack any dissipative processes such as thermal conduction and viscous stresses. Including these effects leads to the Navier–Stokes equations (see, e.g., Landau and Lifshitz 1987). In astrophysical flows, a substantial amount of the energy density, momentum density and stress is in the form of radiation (e.g., photons, neutrinos). Such radiating flows are described by the equation of radiation hydrodynamics (see, e.g., Mihalas and Weibel-Mihalas 1984; Castor 2004). Similarly, magnetic fields play a prominent role and a fluid model is provided by the equations of magnetohydrodynamics (MHD). These more sophisticated physical models and resulting equations possess even richer classes of non-trivial steady states. For example, radiative hydrostatic equilibrium or two-dimensional plasma steady states as described by the Grad-Shafranov equation would be of interest, to name a couple. A (general) relativistic flow description would also be of interest. We hope that the following presentation for the Euler equations can serve as a useful guideline to develop well-balanced schemes for these extended physical models and their more intricate steady states.

### Well-balanced methods for the Euler equations

Many well-balanced schemes for the Euler equations have been developed in the literature. Although the schemes follow different design philosophies, they may be broadly classified based on the steady states of interest they preserve:

1. *A priori known steady states*
  The steady state of interest is assumed to be globally known.
2. *Barotropic steady states*
  The steady states of interest assume a certain barotropic relation, effectively imposing a thermal stratification of the equilibrium state.
3. *Discrete steady states*
  The steady states of interest are built from a consistent discretization of the defining PDE.

The order reflects the successive weakening of the made assumptions about the steady states of interest. Below we classify many of the well-balanced schemes for the Euler equations that are available in the literature into one of these categories. A representative is also presented for each category. This classification and representative selection is a more or less arbitrary choice of the author and as such is not perfect. For instance, some schemes may be classified in several categories, e.g., any barotropic or discrete steady states type scheme can be reduced to an *a priori* type scheme by simply freezing the piecewise steady reconstruction. Indeed, a taxonomy that fits it all may not even exist. Nevertheless, we consider it useful for a first, albeit rough, classification.

#### A priori known steady states

The first category of well-balanced schemes for the Euler equations that we will discuss assumes that the steady state of interest is globally known. This allows the construction of well-balanced finite volume, finite difference and discontinuous Galerkin schemes that can preserve any known steady state, making these methods extremely versatile. The only caveat is, of course, that the steady state has to be known a priori. However, this is not such a severe restriction as it may seem as long as the phenomena of interest don’t deviate too much from the fixed steady state.

Several well-balanced schemes of this category have been developed in the literature:

- Ghosh and Constantinescu (2015, 2016) developed high-order well-balanced finite difference schemes. The schemes are based on the source term decomposition method of Xing and Shu (2013) which is extended to types of equilibria encountered in atmospheric flow simulations. This includes isentropic atmospheres and stratified atmospheres with specified Brunt-Väisälä frequency.
- Li and Xing (2016a) developed high-order well-balanced finite volume schemes for isothermal and polytropic hydrostatic equilibrium on the basis of the source term decomposition method for finite volume schemes of Xing and Shu (2006b, 2013). Li and Xing (2018b) simplify the original procedure of Xing and Shu (2013) by saving some costly WENO reconstructions in the discrete source term evaluation. This is possible because the discrete source term can be evaluated once at the beginning of the simulation since the equilibrium state is known initially. Furthermore, they extend the formalism to more general steady states including isothermal and polytropic hydrostatic equilibrium. Robust high-order discontinuous Galerkin schemes are developed by Wu and Xing (2021) following the techniques introduced by Li and Xing (2016b). These schemes are capable of balancing any known hydrostatic equilibrium, have a guaranteed positivity-preserving property for general equation of states, and can handle unstructured meshes.
- Touma et al. (2016) elaborated a well-balanced second-order unstaggered central finite volume scheme that is able to preserve isothermal equilibrium. The necessary back-and-forth projections between the unstaggered and staggered cells are made equilibrium-preserving by using a variant of the surface gradient method of Zhou et al. (2001). The method provides discrete solutions on a single grid by using a “ghost” staggered grid that is only used in the discrete evolution steps (i.e., it is effectively an unstaggered central scheme).
- Bispen et al. (2017) developed well-balanced second-order finite volume schemes for low Mach-number applications in atmospheric flow. The schemes are based on an implicit-explicit (IMEX) time discretization coupled with a specialized finite volume discretization of the Euler equations in the low Mach number limit. The well-balanced property of the schemes is obtained by subtracting the hydrostatic background stratification from the dynamics.
- Gaburro et al. (2018) developed Arbitrary-Lagrangian-Eulerian (ALE) finite volume schemes on moving nonconforming meshes able to preserve rotational hydrostatic equilibrium. This is accomplished within the well-balanced path-conservative framework of Castro et al. (2008) where the source terms are treated as non-conservative products combined with a well-balanced reconstruction operator.
- Veiga et al. (2019) developed discontinuous Galerkin schemes that can exactly balance any known equilibrium. Furthermore, they systematically compare the well-balanced schemes with unbalanced standard schemes regarding the computational cost for increasingly higher-order schemes. They observe that well-balanced schemes pay off, especially in multi-dimensional settings.
- Berberich et al. (2019) developed second-order well-balanced finite volume schemes for arbitrary known hydrostatic equilibrium. The schemes can handle curvilinear grids and general equations of state (see also Berberich et al. 2018). They are based on known non-dimensionalized density $\alpha$ and pressure $\beta$ functions on which a piecewise steady reconstruction is built and combined with a well-balanced source term discretization. Hence, they term their formalism the $\alpha$-$\beta$ well-balanced method. Klingenberg et al. (2019) generalize the $\alpha$-$\beta$ method to arbitrary orders of accuracy with CWENO reconstruction procedures and Richardson extrapolation for the source term discretization. The latter reconstruction procedures are particularly well suited for the source term discretization as they avoid any negative stencil weights within the cell by construction. Thomann et al. (2020, 2019) combined the $\alpha$-$\beta$ method with relaxation Riemann solvers and implicit-explicit (IMEX) time integration tailored for the efficient treatment of low Mach-number flows.
- Li and Gao (2021) devised a strategy to build high-order well-balanced finite difference schemes by introducing specialized nonlinear WENO differential operators which fulfill a certain homogenization condition that guarantees the exact balance between the flux and source terms. The latter are discretized with the source term decomposition method.
- Berberich et al. (2021a) designed a general framework to construct high-order well-balanced finite volume schemes for any known solution of a given hyperbolic system. Their framework also preserves known time-dependent solutions, which may be interesting for certain applications such as uncertainty quantification. Kanbar et al. (2020) applied the formalism to unstaggered, second-order, central finite volume schemes.
- Edelmann et al. (2021) propose an interesting comparison of several well-balanced finite volume solvers for low Mach-number flows that are relevant for multi-dimensional stellar structure and evolution simulations. Moreover, they developed a multi-dimensional extension of the Cargo and LeRoux (1994) scheme. They emphasize that when combining well-balanced schemes with low Mach number solvers, care must be taken not to introduce spurious numerical artifacts. Low Mach-number solvers rely on special numerical flux functions that reduce the unphysical numerical diffusion in low Mach-number regimes. This can interfere negatively with the vanishing diffusion of well-balanced schemes at steady states.

We next sketch two representative of this category of well-balanced methods. We opted for the $\alpha$-$\beta$ well-balanced finite volume method for hydrostatic equilibrium of Berberich et al. (2018, 2019) and collaborators. Subsequently, we also briefly describe another method that relies on a slightly different principle than most of the methods of the present category.

#### $\alpha$-$\beta$ well-balanced method

For simplicity, we consider a one-dimensional setting and limit the spatial accuracy to second-order. The $\alpha$-$\beta$ well-balanced method assumes that the hydrostatic equilibrium Eq. (173) to be preserved is explicitly known in terms of two dimensionless scalar functions $\alpha (x)$ and $\beta (x)$,

177 $$\begin{aligned} \rho _{eq}(x) = \rho _{0} \alpha (x) \quad {\text {and}} \quad p_{eq}(x) = p_{0} \beta (x) , \end{aligned}$$

fulfilling

178 $$\begin{aligned} \frac{1}{\rho _{eq}} \frac{{\mathrm {d}}p_{eq}}{{\mathrm {d}}x} = - \frac{{\mathrm {d}}\phi }{{\mathrm {d}}x} . \end{aligned}$$

The constants $\rho _{0}$ and $p_{0}$ anchor the equilibrium density and pressure at some reference coordinate $x_{0}$. The steady state of interest $\varvec{U}_{eq}(x)$ in Eq. (45) is therefore explicitly known

179 $$\begin{aligned} \varvec{U}_{eq}(x) = \begin{bmatrix} \rho _{eq}(x) \\ 0 \\ \rho e_{eq}(x) \end{bmatrix} = \begin{bmatrix} \rho _{eq}(x) \\ 0 \\ \rho e(\rho _{eq}(x) , p_{eq}(x)) \end{bmatrix} = \begin{bmatrix} \rho _{0} \alpha (x) \\ 0 \\ \rho e \left( \rho _{0} \alpha (x), p_{0} \beta (x) \right) \end{bmatrix} . \end{aligned}$$

Here the equilibrium internal energy density is computed from the equilibrium density and pressure through the EoS. A well-balanced finite volume scheme based on the $\alpha$-$\beta$ could now be derived in a straightforward manner along the recipe in Sect. 2.4. However, we now switch to primitive instead of the conserved variables to follow Berberich et al. (2018, 2019)’s original presentation. The steady state of interest is then simply

180 $$\begin{aligned} \varvec{W}_{eq}(x) = \begin{bmatrix} \rho _{eq}(x) \\ 0 \\ p_{eq}(x) \end{bmatrix} = \begin{bmatrix} \rho _{0} \alpha (x) \\ 0 \\ p_{0} \beta (x) \end{bmatrix} , \end{aligned}$$

which also highlights the importance of the functions $\alpha (x)$ and $\beta (x)$.

We begin by the piecewise steady reconstruction in primitive variables. Berberich et al. (2018, 2019) use the relative form Eq. (52). Since the steady state of interest is explicitly known, the local equilibrium reconstruction within each cell $\varOmega _{i}$ is trivial. Indeed, the equilibrium projection and matching steps simplify to a formality

181 $$\begin{aligned} \overline{\varvec{W}}_{eq,i} = \begin{bmatrix} \overline{\rho }_{eq,i} \\ 0 \\ \overline{p}_{eq,i} \end{bmatrix} = \begin{bmatrix} \rho _{0} \overline{\alpha }_{i} \\ 0 \\ p_{0} \overline{\beta }_{i} \end{bmatrix} \quad {\text {and}} \quad \varvec{W}_{eq,i}(x) = \varvec{W}_{eq}(x) . \end{aligned}$$

The cell-averaged equilibrium density $\overline{\rho }_{eq,i}$ and pressure $\overline{p}_{eq,i}$ or, equivalently, the $\overline{\alpha }_{i}$ and $\overline{\beta }_{i}$ can be computed over the whole computational domain once at the beginning of the simulation and stored. The local relative equilibrium perturbation reconstruction Eq. (53) gives

182 $$\begin{aligned} \widetilde{\delta \varvec{W}}_{i}(x) = \begin{bmatrix} \mathcal {R} \left( x; \left\{ \frac{\overline{\rho }_{k}}{\overline{\rho }_{eq,k}} \right\} _{k\in S_{i}} \right) \\ \mathcal {R} \left( x; \left\{ v_{x,k} \right\} _{k\in S_{i}} \right) \\ \mathcal {R} \left( x; \left\{ \frac{p_{k}}{\overline{p}_{eq,k}} \right\} _{k\in S_{i}} \right) \end{bmatrix} , \end{aligned}$$

where the velocity and pressure are computed from the cell-averaged conserved variables

183 $$\begin{aligned} v_{x,k} = \frac{\overline{\rho v}_{x,k}}{\overline{\rho }_{k}} \quad {\text {and}} \quad p_{k} = p \left( \overline{\rho }_{k}, \overline{E}_{k} - \frac{\overline{\rho }_{k}}{2} v_{x,k}^2 \right) . \end{aligned}$$

The latter choice limits the spatial accuracy to formally second-order, regardless of whether a higher-order reconstruction procedure $\mathcal {R}$ is used. Note that the velocity component uses a standard reconstruction since it does not participate in the steady state of interest. If we further assume that the reconstruction procedure fulfills a certain scale invariance property^16^

184 $$\begin{aligned} \mathcal {R} \left( x; \left\{ C \overline{Q}_{k} \right\} _{k\in S_{i}} \right) = C \mathcal {R} \left( x; \left\{ \overline{Q}_{k} \right\} _{k\in S_{i}} \right) \end{aligned}$$

for any cell-averaged quantity $\overline{Q}_{k}$ and constant *C*, we obtain the following final expression of the piecewise steady reconstruction

185 $$\begin{aligned} \varvec{W}_{i}(x) = \begin{bmatrix} \alpha (x) \mathcal {R} \left( x; \left\{ \frac{\overline{\rho }_{k}}{\overline{\alpha }_{k}} \right\} _{k\in S_{i}} \right) \\ \mathcal {R} \left( x; \left\{ v_{x,k} \right\} _{k\in S_{i}} \right) \\ \beta (x) \mathcal {R} \left( x; \left\{ \frac{p_{k}}{\overline{\beta }_{k}} \right\} _{k\in S_{i}} \right) \end{bmatrix} . \end{aligned}$$

It is obvious that if equilibrium cell-averages $\overline{\varvec{W}}_{eq,i}$ are given to the above piecewise steady reconstruction, it reduces to the exact steady state Eq. (180) by construction.

It remains to discuss the well-balanced source term discretization. Berberich et al. (2018, 2019) use the alternative form Eq. (59). Since only the momentum source term is relevant at the steady state of interest, we directly obtain

186 $$\begin{aligned} \overline{S}_{\rho v_{x},i} = \frac{1}{2} \left( \frac{\rho _{i}(x_{i-1/2})}{\alpha (x_{i-1/2})} + \frac{\rho _{i}(x_{i+1/2})}{\alpha (x_{i+1/2})} \right) \frac{p_{0}}{\rho _{0}} \frac{\beta (x_{i+1/2}) - \beta (x_{i-1/2})}{\varDelta x} . \end{aligned}$$

Moreover, Berberich et al. (2018, 2019) use the conservative formulation of the total (fluid and gravitational) energy Eq. (169). The complete source term discretization thus reads

187 $$\begin{aligned} \overline{\varvec{S}}_{i} = \begin{bmatrix} 0 \\ \overline{S}_{\rho v_{x},i} \\ 0 \end{bmatrix} . \end{aligned}$$

It is straightforward to verify that the $\alpha$-$\beta$ method is able to preserve any known hydrostatic equilibrium Eq. (179) or Eq. (180). The extension to several space dimension is also uncomplicated (e.g., following the recipe in Sect. 2.6). Extending the method beyond second-order accuracy is slightly more tricky. The issue stems from the pressure reconstruction Eqs. (182) and (185), which estimates the pressure based on the cell-averaged conserved variables Eq. (183). We refer to Klingenberg et al. (2019) for the details.

#### Equilibrium truncation error annihilation method

Let us mention another approach that exists as “folklore” among practitioners. An exact balance of an *a priori* known steady state is simply obtained by subtracting the discretization error at steady state in each time step. At the analytical level and in the infinite resolution limit, this is tantamount to subtracting a zero from the equation (because the steady state fulfills the balance by definition, of course). In semi-discrete evolution form, this simply reads

188 $$\begin{aligned} \frac{{\mathrm {d}}\overline{\varvec{U}}_{i}}{{\mathrm {d}}t} = {\mathcal {L}}(\overline{\varvec{U}})_{i} - {\mathcal {L}}(\overline{\varvec{U}}_{eq})_{i} , \end{aligned}$$

where ${\mathcal {L}}$ is the spatial discretization operator (see Eqs. (7) and (95) for finite volume) and $\overline{\varvec{U}}_{eq}$ the explicitly computable cell averages of the known steady state $\varvec{U}_{eq}$. Similarly, the same can be ported to other spatial discretizations such as finite difference and discontinuous Galerkin methods. For example, this method was used by Dedner et al. (2001) for the simulation of waves in stratified stellar atmospheres. The latter approach is probably easier and computationally cheaper to implement in an existing solver since the equilibrium discretization error ${\mathcal {L}}(\overline{\varvec{U}}_{eq})$ can be calculated once and for all at the beginning of a simulation. However, this approach may interact in less predictable ways within the reconstruction steps than the previously discussed methods such as the $\alpha$-$\beta$ method.

#### Barotropic steady states

The second category of well-balanced schemes for the Euler equations is designed to preserve barotropic steady states. In barotropic fluids, the density is a function of pressure only. This establishes a thermal equilibrium stratification and that information is directly exploited by the well-balanced schemes in this category.

A prominent example is provided by isentropic conditions in which the specific entropy is constant. Consider the fundamental thermodynamic relation

189 $$\begin{aligned} {\mathrm {d}}h = T {\mathrm {d}}s + \frac{{\mathrm {d}}p}{\rho } , \end{aligned}$$

where *h* is the specific enthalpy, *T* the temperature and *s* the specific entropy. Hydrostatic equilibrium Eq. (173) under isentropic conditions (${\mathrm {d}}s = 0$) then gives

190 $$\begin{aligned} \frac{\nabla {p}}{\rho } = \nabla {h} = - \nabla {\phi } , \end{aligned}$$

which can be trivially integrated to

191 $$\begin{aligned} h + \phi = {\text {const}}. \end{aligned}$$

Note that this is an explicit expression for hydrostatic equilibrium under isentropic conditions (assuming the gravitational potential is known). Once the constant fixed at some reference coordinate, the hydrostatic stratification is fully determined. Bernoulli’s Eq. (175) is the generalization to steady adiabatic flow.

Along the same lines, isothermal hydrostatic equilibrium is derived from the fundamental thermodynamic relation

192 $$\begin{aligned} {\mathrm {d}}g = \frac{{\mathrm {d}}p}{\rho } - s {\mathrm {d}}T , \end{aligned}$$

where *g* is the specific Gibbs free energy, yielding

193 $$\begin{aligned} g + \phi = {\text {const.}} \end{aligned}$$

More generally, the expression

194 $$\begin{aligned} \varTheta = \int \frac{{\mathrm {d}}p}{\rho } \end{aligned}$$

is integrable for barotropic fluids and hydrostatic equilibrium takes the form

195 $$\begin{aligned} \varTheta + \phi = {\text {const}} . \end{aligned}$$

Here $\varTheta = \varTheta (\vartheta ,p)$ is a thermodynamic potential depending on the natural variables $\vartheta$ and *p*. For example, the general expression encompasses the isentropic ($\varTheta = h$, $\vartheta = s$), the isothermal ($\varTheta = g$, $\vartheta = T$) and the polytropic^17^ ($\varTheta = \frac{\gamma ^{\prime }}{\gamma ^{\prime } - 1} \frac{p}{\rho }$, $\vartheta = K$) cases.

The above explicit expressions for barotropic hydrostatic equilibrium can then be used for the design of well-balanced schemes. Many schemes of this category have been developed in the literature:

- Botta et al. (2004) designed a well-balanced second-order finite volume scheme for isentropic hydrostatic equilibrium as frequently encountered in numerical weather prediction and climate modeling. The scheme is based on a local piecewise steady reconstruction of isentropic hydrostatic states within each grid cell that are adapted to the local thermodynamic conditions at each time step.
- Fuchs et al. (2010a) constructed second-order finite volume schemes that preserve isothermal hydrostatic equilibrium. The schemes use a piecewise linear steady reconstruction of isothermal hydrostatic states explicitly exploiting the exponential stratification of density and pressure. Remarkably, the schemes are also well-balanced for certain magnetostatic equilibria such as used in the simulation of waves in stellar atmospheres (Rosenthal et al. 2002).
- LeVeque (2010) developed a well-balanced second-order method for polytropic gas dynamics using the *f*-wave approach of Bale et al. (2002). The particular source term average at cell interfaces to guarantee the exact preservation of steady states is constructed using the theory of path conservative methods. Gundlach and LeVeque (2011) used the approach to study the universality in the run-up of shock waves to stellar surfaces. Ahmad and Lindeman (2007) have applied the *f*-wave approach to atmospheric flow problems and they report promising results.
- Xu et al. (2010), Luo et al. (2011) constructed symplecticity-preserving gas-kinetic schemes for compressible Euler and Navier–Stokes equations with gravity. The second-order schemes represent the gravitational potential as a piecewise constant with a potential jump at every cell interface. The schemes are designed such that an isothermal hydrostatic state is preserved during the process of particle transport and collision, which necessitates the use of an exact Maxwellian velocity distribution. See also the recent approach by Chen et al. (2020).
- Xing and Shu (2013) designed high-order well-balanced finite difference schemes for isothermal hydrostatic equilibrium. The approach uses the source term decomposition method of Xing and Shu (2006a) (see Sect. 2.7.3) using the special form of isothermal hydrostatic states. Li and Xing (2016b) extend the formalism to discontinuous Galerkin schemes using the source term decomposition of Xing and Shu (2006b).
- Käppeli and Mishra (2014), Käppeli (2017) developed second-order finite volume schemes that retain barotropic hydrostatic states exactly (up to machine precision). The schemes are based on the piecewise steady reconstruction of barotropic hydrostatic states using thermodynamic potentials and are capable of dealing with general EoS. Grosheintz-Laval and Käppeli (2019); Grosheintz-Laval (2021) extended the schemes to (spatially) arbitrary order of accuracy and unstructured meshes. Grosheintz-Laval and Käppeli (2020) generalized the second-order schemes to adiabatic steady flows.
- Chandrashekar and Klingenberg (2015) constructed well-balanced second-order finite volume schemes for isothermal and polytropic hydrostatic equilibrium. The schemes rewrite the gravitational source terms in a specific form by exploiting the structure of the equilibrium state (similar to Xing and Shu 2013) and a piecewise steady reconstruction that uses equilibrium scaled variables.
- Chandrashekar and Zenk (2017) designed high-order nodal discontinuous Galerkin methods for isothermal and polytropic hydrostatic equilibrium. The schemes use a form of the source term decomposition method as Xing and Shu (2013) combined with Gauss-Lobatto-Legendre quadrature rules. The latter choice ensures that at an equilibrium the solution is continuous between the cells and the flux and source discretizations exactly match.
- Li and Xing (2018a) developed high-order (modal) discontinuous Galerkin schemes capable of preserving isothermal and polytropic hydrostatic states. The schemes are based on a generalized hydrostatic reconstruction with an equilibrium state recovery technique and a special projection operator guaranteeing the necessary continuity conditions of the numerical fluxes at the cell interfaces and a well-balanced source term discretization following Xing and Shu (2006c).
- Gómez-Bueno et al. (2021a, 2021b) developed a general framework for constructing high-order well-balanced finite volume schemes for one-dimensional balance laws. The schemes are based on a piecewise steady reconstruction that constructs local equilibrium by solving the steady-state defining ODEs numerically. (In general, the schemes could therefore also be classified in the discrete steady state category.) In the context of the Euler equations, high-order well-balanced schemes for adiabatic sub- and supersonic steady flows are designed within the framework. By assuming adiabatic flow, they derive a system of ODEs for the steady states that is then solved numerically to derive the piecewise steady reconstruction.

In the following, we briefly outline a representative out of this category of well-balanced schemes for barotropic hydrostatic equilibrium. We opt for the well-balanced finite volume schemes of Käppeli and Mishra (2014), Käppeli (2017) and their higher-order extension by Grosheintz-Laval and Käppeli (2019). This (admittedly not entirely impartial) choice is motivated by the versatility of the approach as it seamlessly adapts to any barotropic relation and relies only on fundamental thermodynamic relations (i.e., it works for any EoS). We now follow the recipe from Sect. 2.4 and prepare the necessary ingredients to design a one-dimensional well-balanced finite volume scheme for barotropic hydrostatic equilibrium. The multi-dimensional case is briefly addressed at the end of the description.

The starting point is to ensure the computability of the steady states of interest. The barotropic hydrostatic equilibrium Eq. (195) immediately gives the following equilibrium profiles

196 $$\begin{aligned} \vartheta _{eq}(x) = \vartheta _{0} \quad {\text {and}} \quad \varTheta _{eq}(x) = \varTheta _{0} + \phi _{0} - \phi (x) . \end{aligned}$$

Here $\vartheta _{0}$, $\varTheta _{0}$ and $\phi _{0}$ are the equilibrium’s thermodynamic natural variable and potential, and the gravitational potential evaluated at some reference coordinate $x_{0}$, respectively. The equilibrium conserved variables are then

197 $$\begin{aligned} \varvec{U}_{eq}(x) = \begin{bmatrix} \rho _{eq}(x) \\ 0 \\ \rho e_{eq}(x) \end{bmatrix} = \begin{bmatrix} \rho (\vartheta _{0},\varTheta _{eq}(x)) \\ 0 \\ \rho e(\vartheta _{0},\varTheta _{eq}(x)) \end{bmatrix} , \end{aligned}$$

where the equilibrium density $\rho _{eq}(x)$ and internal energy density $\rho e_{eq}(x)$ are computed through the EoS given the natural variable and thermodynamic potential. Note that we distinguish between the exact $\varvec{u}_{eq}(x)$ and approximate $\varvec{U}_{eq}(x)$ steady states of interest (see Eq. (45)). Although this may seem overly pedantic, it takes into account that the gravitational potential $\phi (x)$ may only be known approximately (say up to order $\mathcal {O}(\varDelta x^{\epsilon })$).

The next ingredient is the piecewise steady reconstruction from the cell-averaged conserved variables $\{\overline{\varvec{U}}_{i}\}$. It consists of reconstructing within each cell $\varOmega _{i}$ local equilibrium $\varvec{U}_{eq,i}(x)$ and perturbation $\delta \varvec{U}_{i}(x)$ parts. The local equilibrium is fixed in two substeps. First, the cell average $\overline{\varvec{U}}_{i}$ is projected onto a cell average that is consistent with the steady states of interest. This is of course trivial for the density and the momentum components. For the energy component, an estimate of the cell-averaged internal density is needed. A natural choice is provided by

198 $$\begin{aligned} \overline{E}_{eq,i} = \overline{\rho e}_{eq,i} \approx \overline{E}_{i} - \frac{1}{2} \frac{\overline{\rho v}_{x,i}^{2}}{\overline{\rho }_{i}} . \end{aligned}$$

Note that this choice is consistent with the steady states of interest (i.e., it is exact at equilibrium when $\overline{\rho v}_{x,i} \equiv 0$). With this we then have the local equilibrium projected cell-averaged conserved variables

199 $$\begin{aligned} \overline{\varvec{U}}_{eq,i} = \begin{bmatrix} \overline{\rho }_{i} \\ 0 \\ \overline{\rho e}_{eq,i} \end{bmatrix} . \end{aligned}$$

The second substep matches a steady state of interest profile Eq. (197) to the equilibrium projected cell averages $\overline{\varvec{U}}_{eq,i}$ by Eq. (47). To this end, the local equilibrium profiles are anchored at the cell center $x_{i}$^18^

200 $$\begin{aligned} \vartheta _{eq,i}(x) = \vartheta _{0,i} \quad {\text {and}} \quad \varTheta _{eq,i}(x) = \varTheta _{0,i} + \phi _{i} - \phi (x) , \end{aligned}$$

where the $\vartheta _{0,i}$, $\varTheta _{0,i}$ and $\phi _{i} = \phi (x_{i})$ are point values of the equilibrium’s natural variable, thermodynamic potential and gravitational potential at cell center. It is assumed that the gravitational potential $\phi (x)$ can be evaluated anywhere needed, either exactly or approximately (up to some order $\mathcal {O}(\varDelta x^{\epsilon })$). The anchor values $\vartheta _{0,i}$ and $\varTheta _{0,i}$ are then set by requiring that

201 $$\begin{aligned} &\overline{\rho }_{i}= \frac{1}{\varDelta x} {\mathcal {Q}}_{i} \left( \rho _{eq,i} \right) = \frac{1}{\varDelta x} \sum _{\alpha =1}^{N_{q}} \omega _{\alpha } ~ \rho _{eq,i}\left( \vartheta _{0,i}, \varTheta _{eq,i}(x_{i,\alpha })\right) , \\& \overline{\rho e}_{i} = \frac{1}{\varDelta x} {\mathcal {Q}}_{i} \left( \rho e_{eq,i} \right) = \frac{1}{\varDelta x} \sum _{\alpha =1}^{N_{q}} \omega _{\alpha } ~ \rho e_{eq,i}\left( \vartheta _{0,i}, \varTheta _{eq,i}(x_{i,\alpha })\right) . \end{aligned}$$

In general, Eq. (201) represents a system of two (non-linear) equations for the anchor values $\vartheta _{0,i}$ and $\varTheta _{0,i}$ of the local equilibrium reconstruction profile in cell $\varOmega _{i}$. The system can efficiently be solved by a (hybrid) Newton method (see, e.g., Press et al. 1993; Dennis and Schnabel 1996). We remark that the derivatives needed for the Jacobian matrix computation in Newton’s method are of thermodynamic nature and provided by any EoS. Moreover, the initial guesses for $\vartheta _{0,i}$ and $\varTheta _{0,i}$ can directly be computed from the local equilibrium projected cell averages $\overline{\varvec{U}}_{eq,i}$ through the EoS, i.e.,

202 $$\begin{aligned} \vartheta _{0,i} = \vartheta (\overline{\rho }_{i}, \overline{\rho e}_{eq,i}) \quad {\text {and}} \quad \varTheta _{0,i} = \varTheta (\overline{\rho }_{i}, \overline{\rho e}_{eq,i}) . \end{aligned}$$

Note that the latter values are second-order approximation of the values at cell center and are therefore already pretty close to the solution. Furthermore, these initial guesses are already the solution of the system Eq. (201) for a second-order scheme that uses the midpoint rule. Equipped with the local equilibrium reconstruction,

203 $$\begin{aligned} \varvec{U}_{eq,i}(x) = \begin{bmatrix} \rho _{eq,i}(x) \\ 0 \\ \rho e_{eq,i}(x) \end{bmatrix} , \end{aligned}$$

the local equilibrium perturbation $\delta \varvec{U}_{i}(x)$ can be computed following the recipe in Sect. 2.4.1. As a result, we obtain a piecewise steady reconstruction ${\mathcal{WR}}$ for barotropic hydrostatic equilibrium. Likewise, the well-balanced source term discretization is obtained as described in Sect. 2.4.2. The latter can also be constructed such that the scheme is total (fluid and gravitational) energy-conserving. This can be achieved either by evolving the total energy Eq. (169) directly or by discretizing the source term in Eq. (170) appropriately.^19^

This concludes the description of the required ingredients to assemble a one-dimensional finite volume scheme that preserves a discrete form of barotropic hydrostatic equilibrium. A particularity of that steady state Eq. (195) is its validity in several space dimensions. Therefore, a well-balanced finite volume scheme for multi-dimensional barotropic hydrostatic equilibrium can readily be developed according the recipe in Sect. 2.6.

#### Discrete steady states

The third category of well-balanced schemes for the Euler equations avoid any assumption on the thermal stratification of the steady states. In effect, they aim at preserving a consistent discretization of the PDE underlying the steady states of interest. For hydrostatic equilibrium, this is

$$\begin{aligned} \nabla {p} = - \rho \nabla {\phi } , \end{aligned}$$

which the methods in this category solve numerically (given the density and gravitational acceleration) for the hydrostatic pressure. Therefore, the methods in this category are, in some sense, truly in the spirit of the piecewise steady reconstruction as early advocated by van Leer (1984), Eulderink and Mellema (1995), Mellema et al. (1991). However, this endeavor is difficult in general, especially in several space dimensions. We will come back briefly to this issue at the end of the section.

Many schemes of this type have been developed in the literature:

- LeVeque and Bale (1999) developed second-order finite volume schemes that preserve hydrostatic equilibrium *and* steady adiabatic flow (see also LeVeque et al. 1998). The schemes are based on the quasi-steady wave-propagation algorithm of LeVeque (1998). The method replaces the piecewise constant solution representation within a cell with two constant states separated by a single discontinuity at the middle of the cell. The jump within the cell is chosen such that it exactly cancels out the source term. This leads to modified Riemann problems at cell interfaces that only encode perturbations from the steady state. The steady states are preserved with second-order accuracy by construction, and perturbations are propagated with the same accuracy within the wave-propagation algorithm.
- Fuchs et al. (2011) presented well-balanced second-order finite volume schemes for stratified non-isothermal magnetic atmospheres (see also Fuchs et al. 2010b). The schemes are based on an extension beyond the isothermal case developed by Fuchs et al. (2010a) and are capable of preserving certain non-isothermal magnetostatic equilibria. The schemes are applied to the simulation of waves in the outer solar (chromosphere and corona) and other stellar atmospheres. A similar approach is used by Krause (2019).
- Vides et al. (2014) developed a second-order finite volume schemes for self-gravitating astrophysical flows. Although the schemes are not strictly well-balanced^20^ (according to the authors), they nevertheless display a substantial improvement over a standard scheme. The schemes are based on a relaxation-type Riemann solver that incorporates the gravity source terms, which effectively couples more strongly the fluid to the gravitational forces. Padioleau et al. (2019) extend the method to low Mach number flow regimes and apply it to compressible convection.
- Desveaux et al. (2015) constructed a well-balanced finite volume scheme to capture non-explicit hydrostatic equilibria (see also Desveaux 2013; Desveaux et al. 2014). It is based on a relaxation-type Riemann solver able to resolve hydrostatic equilibrium by directly including the gravity source terms. The resulting scheme preserves a spatially second-order accurate discrete form of hydrostatic equilibrium, but perturbations are only evolved with first order accuracy.
- Käppeli and Mishra (2016) designed well-balanced second-order finite volume schemes for hydrostatic sates. They are based on a local discrete hydrostatic reconstruction that directly integrates the equilibrium pressure given the density and gravity forces (see also Käppeli and Mishra 2015). Popov et al. (2019) present some first applications of the schemes to multi-dimensional stellar structure calculations. Moreover, they present an improved discretization of the gravity source term in the energy equation that avoids unphysical energy changes when simulating quasi-stationary convection for extended time periods. Grosheintz-Laval (2021) adapted the schemes to semi-structured icosahedral grids. Berberich et al. (2021b) generalize the second-order schemes to arbitrary orders of accuracy.
- Franck and Mendoza (2016) constructed well-balanced asymptotic-preserving finite volume schemes for the Euler equations with gravity and friction source terms. The schemes are based on a Lagrange+remap approach in combination with a relaxation procedure specifically designed to capture the asymptotic limit induced by the friction source term (i.e., the scheme produces consistent and stable approximations for arbitrarily high friction coefficients). Moreover, the schemes are capable of preserving an arbitrary high-order discretization of hydrostatic equilibrium, but perturbations are only evolved with first order accuracy. An extension to two-dimensional unstructured meshes is also presented.
- Chertock et al. (2018) developed second-order central-upwind finite volume schemes capable of preserving hydrostatic equilibrium. The schemes are based on a purely conservative reformulation of the equations that avoids the source terms by introducing global fluxes, which essentially corresponds to an integration of the hydrostatic pressure over the whole computational domain. The inherent viscosity of the central-upwind scheme is tweaked by a smooth cut-off function that tends towards zero when the computed solution is locally close to a steady state. See also Gascón and Corberán (2001), Caselles et al. (2009) for a closely related approach.
- Varma and Chandrashekar (2019) extended the approach of Chandrashekar and Klingenberg (2015) to arbitrary thermal stratification. To construct the hydrostatic reconstruction and well-balanced source term discretization, they first rewrite the hydrostatic equilibrium in a particular form and discretize the resulting expressions. Effectively, this allows the parametrization of the assumed subcell thermal equilibrium in the piecewise steady reconstruction.

In the following, we briefly sketch in a one-dimensional setting the well-balanced schemes of Käppeli and Mishra (2016) in their arbitrary high-order formulation by Berberich et al. (2021b). Again, this (to be sure not fully impartial) choice is motivated by the flexibility of the approach as it easily adapts to any EoS. The plan is to prepare all the necessary ingredients to apply the recipe outlined in Sect. 2.4. As mentioned earlier, it is currently a challenge to extend the well-balanced schemes of this category to the multidimensional case. Some discussion of this issue is provided at the end of the one-dimensional description.

First of all, we need to be able to compute the steady states of interest. For one-dimensional hydrostatic equilibrium,

204 $$\begin{aligned} \frac{{\mathrm {d}}p_{eq}}{{\mathrm {d}}x} = - \rho _{eq} \frac{{\mathrm {d}}\phi }{{\mathrm {d}}x} , \end{aligned}$$

we obtain by direct integration

205 $$\begin{aligned} p_{eq}(x) = p_{0} - \int _{x_{0}}^{x} \rho _{eq} \frac{{\mathrm {d}}\phi }{{\mathrm {d}}x} {\mathrm {d}}x . \end{aligned}$$

Here, $\rho _{eq}$ and $p_{eq}(x)$ denote the hydrostatic density and pressure, and $p_0{}$ is the pressure at some reference coordinate $x_{0}$. The equilibrium conserved and primitive variables are then

206 $$\begin{aligned} \varvec{U}_{eq}(x) = \begin{bmatrix} \rho _{eq}(x) \\ 0 \\ \rho e_{eq}(x) \end{bmatrix} = \begin{bmatrix} \rho _{eq}(x) \\ 0 \\ \rho e(\rho _{eq}(x), p_{eq}(x)) \end{bmatrix} , \quad \varvec{W}_{eq}(x) = \begin{bmatrix} \rho _{eq}(x) \\ 0 \\ p_{eq}(x) \end{bmatrix} , \end{aligned}$$

where the equilibrium energy density $\rho e_{eq}(x)$ is computed through the EoS. Note that we are again distinguishing the exact $\varvec{u}_{eq}(x)$ and the approximate $\varvec{U}_{eq}(x)$ hydrostatic equilibrium state (see Eq. (45)). Both, the equilibrium density and the gravitational potential will be approximated by (polynomial) reconstruction and interpolation (up to some desired accuracy $\mathcal {O}(\varDelta x^{\epsilon })$).

The second ingredient is the piecewise steady reconstruction, whose goal is to build accurate equilibrium subcell profiles from the cell-averaged conserved variables $\{\overline{\varvec{U}}_{i}\}$ that are consistent with the steady states of interest. This local equilibrium profile is found by fitting an equilibrium Eq. (206) to the cell’s average $\overline{\varvec{U}}_{i}$. The first substep is to perform an equilibrium projection of $\overline{\varvec{U}}_{i}$ onto a cell average $\overline{\varvec{U}}_{eq,i}$ consistent with hydrostatic equilibrium. A convenient option is as in the barotropic equilibrium case provided by

207 $$\begin{aligned} \overline{\varvec{U}}_{eq,i} = \begin{bmatrix} \overline{\rho }_{i} \\ 0 \\ \overline{\rho e}_{eq,i} \end{bmatrix} , \end{aligned}$$

where the cell-averaged equilibrium internal energy density is estimated directly from the cell average $\overline{\varvec{U}}_{i}$ as

208 $$\begin{aligned} \overline{E}_{eq,i} = \overline{\rho e}_{eq,i} \approx \overline{E}_{i} - \frac{1}{2} \frac{\overline{\rho v}_{x,i}^{2}}{\overline{\rho }_{i}} . \end{aligned}$$

This choice is exact at hydrostatic equilibrium $\overline{\rho v}_{x,i} \equiv 0$ (i.e., it is consistent with the steady states of interest). The second substep matches a hydrostatic equilibrium profile Eq. (205) to the equilibrium projected cell average $\overline{\varvec{U}}_{eq,i}$ by Eq. (47). For that purpose, the local equilibrium pressure profile $p_{eq,i}(x)$ is anchored at the cell center $x_{i}$^21^

209 $$\begin{aligned} p_{eq,i}(x) = p_{0,i} - \int _{x_{i}}^{x} \rho _{eq,i}(x) \left( \frac{{\mathrm {d}}\phi }{{\mathrm {d}}x}\right) _{i}(x) ~ {\mathrm {d}}x , \end{aligned}$$

where $p_{0,i}$ is a point value of the equilibrium pressure at cell center, $\rho _{eq,i}(x)$ is the local equilibrium density reconstruction and $\left( {\mathrm {d}}\phi /{\mathrm {d}}x\right) _{i}(x)$ the gravitational acceleration in cell $\varOmega _{i}$. Before we can evaluate Eq. (209), we have to choose a subcell representation for these quantities. The gravitational acceleration is computed by (polynomially) interpolating the gravitational potential $\{\phi _{i}\}$ and differentiating (even if the potential is analytically known and the derivative could be evaluated exactly). An obvious choice for the local equilibrium density reconstruction $\rho _{eq,i}(x)$ is the one provided by the standard reconstruction procedure for the density (Berberich et al. 2021a):

210 $$\begin{aligned} \rho _{eq,i}(x) = \rho _{i}(x) = \mathcal {R}\left( x; \{\overline{\rho }_{k}\}_{k \in S_{i}} \right) . \end{aligned}$$

**Fig. 7:**
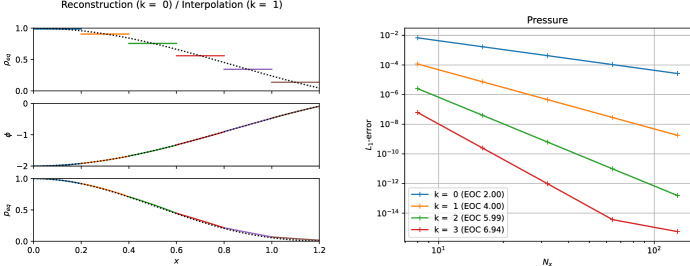
Equilibrium pressure profile for a polytrope of index $n = 1$ ($\gamma = 2$) over the radial interval [0, 1.2] discretized by 6 cells: Examples for the equilibrium density reconstruction $\rho _{eq,i}(x)$ (based on the symmetric stencil $\{\overline{\rho }_{-k}, \dots , \overline{\rho }_{+k}\}$ of size $2 k + 1$) and gravitational potential interpolation $\phi _{i}(x)$ (based on the symmetric stencil $\{\phi _{-k-1}, \dots , \phi _{+k+1}\}$ of the size $2 k + 3$) for the construction of the hydrostatic pressure profile $p_{eq,i}(x)$ from Eq. (209). The left panel displays the equilibrium density as reconstructed with piecewise constants ($k = 0$), the gravitational potential as interpolated with piecewise parabolic ($k = 1$) and the resulting piecewise linear equilibrium pressure profile. The right panel shows the accuracy of the equilibrium pressure profile for several stencil size choices ($k=0, 1, 2, 3$) together with the estimated order of convergence (EOC). We empirically observe that the equilibrium pressure profile has accuracy $\epsilon = 2 (k + 1)$, which is increased by one than one would have expected from the order of the equilibrium density reconstruction. We (presumably) attribute this increase in accuracy to a similar phenomena appearing in numerical integration, where even-degree (i.e., symmetric) Newton–Cotes quadrature rules have a by one higher order of accuracy than expected

However, other choices are possible. See Fig. 7 for a few possibilities. This influences (unsurprisingly) the accuracy $\mathcal {O}(\varDelta x^{\epsilon })$ to which the equilibrium profiles are computed and the overall stencil size (Berberich et al. 2021a). Note that since the integrand in Eq. (209) is a simple polynomial, the integral can be easily evaluated analytically. The anchor value $p_{0,i}$ is then fixed by requiring that the pressure profile Eq. (209) matches with the cell-averaged equilibrium internal energy density

211 $$\begin{aligned} \begin{aligned} \overline{\rho e}_{eq,i}&= \frac{1}{\varDelta x} {\mathcal {Q}}_{i}\left( \rho e(\rho _{eq,i} , p_{eq,i} ) \right) \\&= \frac{1}{\varDelta x} \sum _{\alpha =1}^{N_{q}} \omega _{\alpha } ~ \rho e \left( \rho _{eq,i}(x_{i,\alpha }) , p_{0,i} - \int _{x_{i}}^{x_{i,\alpha }} \rho _{eq,i}(x) \left( \frac{{\mathrm {d}}\phi }{{\mathrm {d}}x}\right) _{i}(x) ~ {\mathrm {d}}x \right) . \end{aligned} \end{aligned}$$

This is a scalar equation for the pressure $p_{0,i}$ at the cell center and it can be efficiently solved iteratively by, e.g., a (hybrid) Newton method (see, e.g., Press et al. 1993; Dennis and Schnabel 1996). The iteration starts with the pressure estimated from the cell-average mass and internal energy density Eq. (208) through the EoS. The initial guess is a second-order approximation of the pressure point value at the cell center and is, therefore, already quite close to the sought solution. Moreover, it can be solved analytically for second-order accurate schemes or simple EoS like the ideal gas law (arbitrary order). The local equilibrium reconstruction in cell $\varOmega _{i}$ is then

212 $$\begin{aligned} \varvec{U}_{eq,i}(x) = \begin{bmatrix} \rho _{eq,i}(x) \\ 0 \\ \rho e(\rho _{eq,i}(x), p_{eq,i}(x)) \end{bmatrix} , \quad \varvec{W}_{eq,i}(x) = \begin{bmatrix} \rho _{eq,i}(x) \\ 0 \\ p_{eq,i}(x) \end{bmatrix} . \end{aligned}$$

The local equilibrium perturbation $\delta \varvec{U}_{i}(x)$ can now be computed as described by the recipe in Sect. 2.4.1. This gives us the complete piecewise steady reconstruction ${\mathcal{WR}}$ for arbitrarily stratified hydrostatic equilibrium. Along the same lines, the well-balanced source term discretization follows from Sect. 2.4.2:

213 $$\begin{aligned} \overline{\varvec{S}}_{i} = - \frac{1}{\varDelta x} \int _{\varOmega _{i}} \begin{bmatrix} 0 \\ \rho _{i}(x) \\ \rho v_{x,i}(x) \end{bmatrix} \left( \frac{{\mathrm {d}}\phi }{{\mathrm {d}}x}\right) _{i}(x) ~ {\mathrm {d}}x . \end{aligned}$$

Remarkably, this is the same discretization as provided by a standard (high-order) finite volume scheme. This is a welcome simplification when implemented into an existing solver. As for the barotropic well-balanced schemes, the source term in the energy equation can be avoided by either evolving the total (fluid and gravitational) energy or by discretizing appropriately the source terms in Eq. (170). Such total energy-conserving schemes are advantageous for the long-term simulation of near-equilibrium configurations.

Unfortunately, the just described one-dimensional well-balanced scheme does not generalize to the multi-dimensional case in a straightforward manner (e.g., by following the recipe in Sect. 2.6). The culprit is that the discrete (re)construction of the hydrostatic equilibrium profile via approximation of Eq. (205),

214 $$\begin{aligned} p_{eq}(\varvec{x}) = p_{0} - \int _{\varGamma } \rho _{eq} \nabla \phi \cdot {\mathrm {d}}\varvec{x} , \end{aligned}$$

is typically dependent on the path $\varGamma$ (from the reference coordinate $\varvec{x}_{0}$ to $\varvec{x}$). Only under special circumstances, such as constant density or alignment of gravity forces with one coordinate axis, a unique construction of the hydrostatic profile in such a direct way is feasible. As a matter of fact, the numerical integration of the hydrostatic profile via Eq. (214) gives sensible results (i.e., path independent) only if a discrete curl operator applied to the reconstructed integrand ($\rho _{eq} \nabla \phi$) vanishes everywhere (i.e., the integrability condition for hydrostatic equilibrium is fulfilled in a discrete sense). Therefore, the direct (line) integration approach seems hopeless. A more promising approach may rely on trying to solve a certain boundary value problem locally:

215 $$\begin{aligned} \nabla ^{2} p = - \nabla \cdot \left( \rho \nabla \phi \right) . \end{aligned}$$

For example, a relaxation method could solve the elliptical PDE within a cell to construct its local hydrostatic pressure distribution (assuming the pressure in the neighboring cells is given). At an equilibrium state, the hope is that the relaxation approach realizes that the cell’s pressure is in equilibrium with its surrounding cells and local gravity forces. Away from an equilibrium state, certain criteria have to be devised to select a plausible local equilibrium state. However, this is beyond the scope of the present review.

However, let us end the present section on an optimistic note. Although not well-balanced in a multi-dimensional sense, it turns out that the above well-balanced scheme considerably improves the simulation of nearly hydrostatic configurations even in this suboptimal case.

### Numerical examples

In this section, we showcase some typical numerical test problems used to assess the performance of well-balanced schemes in the context of the Euler equations. We here deliberately focus on standard test problems that are easily replicable by an interested reader wishing to test his or her implementation/scheme. Using well-balanced schemes in concrete astrophysical applications usually brings in a plethora of complications (initial conditions, appropriate boundary conditions, microphysics, etc.) and goes beyond the scope of this review. Before we proceed, we give general comments on how the problems below are initialized, boundary conditions are handled, the timescale the simulations are run, and how the accuracy is measured.

Given a point-wise defined initial condition in conserved variables $\varvec{u}^{0} = \varvec{u}^{0}(x)$, the initialization of the simulation depends on the nature of the chosen scheme. For finite volume schemes, the cell-averaged conserved variables at the initial time $\overline{\varvec{U}}_{i}^{0}$ are computed by (sufficiently accurate) numerical integration

216 $$\begin{aligned} \overline{\varvec{U}}_{i}^{0} = Q_{i}(\varvec{u}^{0}) . \end{aligned}$$

An appropriate multi-dimensional quadrature rule is used for multi-dimensional problems. Often, it is convenient to formulate the initial conditions in a different set of variables, such as the primitive variables $\varvec{w} = [\rho , \varvec{v}, p]^{T}$, and apply a point-wise transformation $\varvec{u}^{0}(x) = \varvec{u}(\varvec{w}^{0}(x))$ in the above initializations.

Boundary conditions are usually a delicate and strongly application-dependent matter, even more so when the dynamics of interest occurs close to a steady state. Typically, the local equilibrium profile found in the piecewise steady reconstruction in the last physical cells is extrapolated into an appropriate number of ghost cells. For finite volume schemes, the cell averages in the ghost cells are computed by

217 $$\begin{aligned} \begin{aligned} \overline{\varvec{U}}_{k}^{0}&= Q_{k}(\varvec{U}_{eq,1})\quad {\text { for }} k< 1, \\ \overline{\varvec{U}}_{k}^{0} &= Q_{k}(\varvec{U}_{eq,N}) \quad {\text { for }} N < k. \end{aligned} \end{aligned}$$

Another option is to freeze the data in the ghost cells to the initial equilibrium conditions. In multiple dimensions, the boundary conditions are applied in a direction-by-direction manner.

To characterize a timescale on which a model reacts to perturbations of its equilibrium, we define the sound crossing time

218 $$\begin{aligned} \tau _{\mathrm {sound}} = 2 \int _{\varGamma _{eq}} \frac{{\mathrm {d}}x}{c_{s}} , \end{aligned}$$

where $c_{s}$ denotes the speed of sound and the integral has to be taken over the extent of the steady state of interest $\varGamma _{eq}$. The sound crossing time corresponds to the time it takes for a sound wave to propagate back and forth through the equilibrium configuration. It gives a measure of how quickly a steady configuration reacts to any perturbations of its equilibrium.

The accuracy of the schemes is quantified by computing the errors

219 $$\begin{aligned} Err = \Vert q - q^{\mathrm {ref}}\Vert , \end{aligned}$$

where $\Vert .\Vert$ denotes some norm, usually the $L_{1}$-norm. Here *q* is a selected quantity of interest (e.g., density, pressure, velocity, ...) and $q^{\mathrm {ref}}$ is a reference solution. The reference solution is the stationary state to be maintained discretely or a numerically obtained solution on a very fine grid. Although the comparison with a numerically obtained reference solution does not provide rigorous evidence of convergence, it nevertheless indicates a meaningful measure of the errors.

#### Hydrostatic atmospheres

The simplest setup one can image is that of a one-dimensional hydrostatic atmosphere subject to a constant gravitational acceleration *g*:

220 $$\begin{aligned} \frac{{\mathrm {d}}p}{{\mathrm {d}}x} = - \rho g . \end{aligned}$$

Despite its apparent simplicity, it has applications in situations where gravitational forces change slowly with respect to other quantities of interest, such as numerical weather prediction, climate modeling of (exo-) planets, and simulation of waves in stellar atmospheres.

As a matter of fact, Eq. (220) describes only a mechanical equilibrium of the atmosphere. To uniquely integrate Eq. (220), one needs in addition to a density $\rho _{0}$ and pressure $p_{0}$ at the base of the atmosphere $x_{0}$ also a thermal stratification. This is simply because the pressure depends on one^22^ additional thermodynamic quantity besides density such as the temperature *T* ($p = p(\rho ,T)$) or the specific entropy *s* ($p = p(\rho ,s)$). We assume a monoatomic ideal gas law EoS

221 $$\begin{aligned} p = R \rho T = e^{s/c_{v}} \rho ^{\gamma } = (\gamma - 1) \rho e , \end{aligned}$$

where *R* is the gas constant, $c_{v}$ the specific heat at constant volume and $\gamma$ the ratio of specific heats.

For an isothermal atmosphere $T = T_{0} = {\text {const.}}$, one can then immediately integrate Eq. (220) to yield

222 $$\begin{aligned} p_{eq}(x) = p_{0} e^{-\frac{x - x_{0}}{H_{0}}} \quad {\text {and}} \quad \rho _{eq}(x) = \rho _{0} e^{-\frac{x - x_{0}}{H_{0}}} . \end{aligned}$$

Here $H_{0} = R T_{0}/g$ is the so-called scale height. Similarly, under isentropic conditions $s = s_{0} = {\text {const.}}$, Eq. (220) can be analytically integrated to

223 $$\begin{aligned} p_{eq}(x) = e^{s_{0}/c_{v}} \rho (x)^{\gamma } \quad {\text {and}} \quad \rho _{eq}(x) = \left( \rho _{0}^{\gamma - 1} - \frac{g}{e^{s_{0}/c_{v}}} \frac{\gamma - 1}{\gamma } (x - x_{0}) \right) ^{\frac{1}{\gamma - 1}} \end{aligned}$$

with $s_{0} = c_{v}\ln (p_{0}/\rho _{0}^{\gamma })$. Note that the isentropic atmosphere has a surface at a finite height while the isothermal one extends to infinity.

**Fig. 8:**
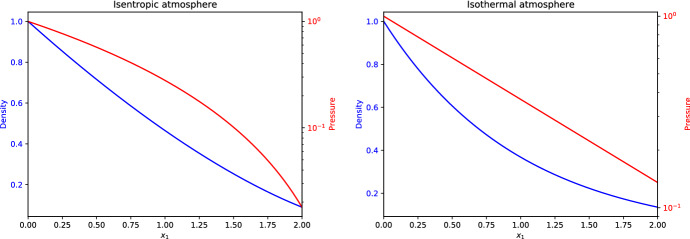
Density and pressure profiles for the isentropic (left panel) and isothermal (right panel) atmospheres

Following Käppeli and Mishra (2016), we set the computational domain to $\varOmega = [0,2]$, and the EoS parameters to $R = 1$, $c_{v} = 3R/2$ and $\gamma = 5/3$. For the isentropic atmosphere, we set the density and pressure to $\rho _{0} = 1$ and $p_{0} = 1$ at the base $x_{0} = 0$. The resulting isentropic atmosphere has a scale height $H(x) = - \frac{{\mathrm {d}}x}{{\mathrm {d}}\ln P}$ decreasing linearly from $H(0) = 1$ at the bottom to $H(2) = 0.2$ at the top and a sound crossing time of $\tau _{\mathrm {sound}} \approx 4.3$. Similarly for the isothermal atmosphere, we set the density and pressure to $\rho _{0} = 1$ and $p_{0} = 1$ at the base $x_{0} = 0$. The resulting isothermal atmosphere has a constant scale height of $H_{0} = 1$ and a sound crossing time of $\tau _{\mathrm {sound}} \approx 3.1$. The density and pressure profiles of both atmospheres are shown in Fig. 8. Similar setups have been used in most (if not all) publications for designing well-balanced methods for the Euler equations with gravity.

Next, we present several test cases based on the simple setting of isothermal and isentropic hydrostatic atmospheres. The results are obtained with the well-balanced second-order finite volume scheme of Käppeli and Mishra (2016), also outlined in Sect. 3.3.3. Below it will be termed as the “well-balanced (WB) discrete scheme” as it belongs to the category of well-balanced schemes with an underlying discrete piecewise steady reconstruction. For the sake of comparison, we also show the results obtained with a standard second-order finite volume scheme obtained by switching off the piecewise steady reconstruction. Below it will be termed the “standard (STD) scheme.”

#### Well-balanced property

The first thing to verify is, of course, the well-balanced property of the scheme: Is it able to preserve the steady state it was designed for up to machine precision? Indeed, due to the finite precision of the computer’s approximation of real numbers, exact balance can, in general, not be expected.

To this end, one initializes the computation with so-called well-prepared initial data, i.e., the discrete steady state that the well-balanced method is designed to balance. These well-prepared initial conditions are then evolved with the well-balanced scheme for a certain time characteristic for the considered steady state, e.g., a multiple of the sound crossing time Eq. (218). At the end of the computation, one computes the difference between the initial and final states in some norm (e.g., the $L_{1}$ norm).

**Fig. 9:**
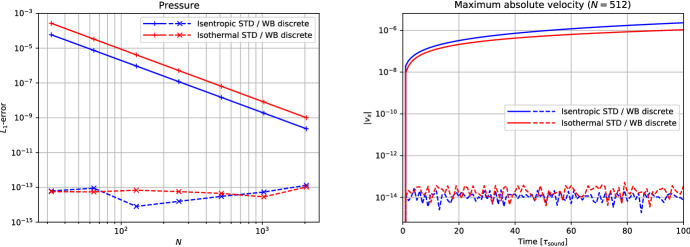
Left panel: $L_1$-norm of the difference between the initial and final pressure for the isentropic (solid and dashed blue lines) and isothermal (solid and dashed red lines) atmospheres after two sound crossing times for several resolutions. The solid and dashed lines represent the simulation performed with the standard and the well-balanced (WB) schemes, respectively. Right panel: The maximum absolute velocity as a function of time for $N = 512$

The results of such one-dimensional computations with the well-balanced and the standard scheme are shown in Fig. 9. From the left panel of the figure, it is clear that the well-balanced scheme maintains the discrete hydrostatic equilibrium down to machine precision. On the other hand, the standard scheme cannot preserve the discrete equilibrium. However, note that the error for the standard scheme gets smaller with increasing resolution.^23^ Indeed, in the limit of an extremely high resolution, the unbalanced scheme can also preserve the equilibrium. This is a matter of consistency of the numerical method with the PDE.

The right panel of Fig. 9 shows the evolution of the maximum absolute velocity for a given resolution ($N = 512$ cells) and up to one hundred sound crossing times. The advantage of well-balanced methods for long-term integration of near-equilibrium flows becomes quite clear: the local truncation errors of the standard scheme piles up in each step and create spurious flow. Conversely, only the (unavoidable) round-off errors accumulate for the well-balanced scheme. This makes such well-balanced schemes well suited to study numerically natural phenomena that occur on a hydrostatic background.

#### Wave propagation: shake the base

The next test assesses the ability of a scheme to propagate waves on top of a hydrostatic atmosphere. For brevity, only the results for the isentropic atmosphere are shown. Fuchs et al. (2010a) suggest to excite waves at the bottom of the atmosphere by imposing a periodic velocity perturbation in the lower boundary

$$\begin{aligned} v_{x,m}(t) = A \sin \left( 6 \frac{2 \pi t}{t_{f}} \right) , \end{aligned}$$

where $m < 1$ is the boundary cell index (i.e., ghost cell index) and *A* is the amplitude of the perturbation. The simulation is stopped shortly before the excited waves hit the upper boundary at $t_{f} = 1.8$. Practically, this test is a simplified version of similar setups used in the study of wave propagation in stellar atmospheres (see, e.g., Bogdan et al. 2003; Rosenthal et al. 2002; Fuchs et al. 2010c and references therein).

**Fig. 10:**
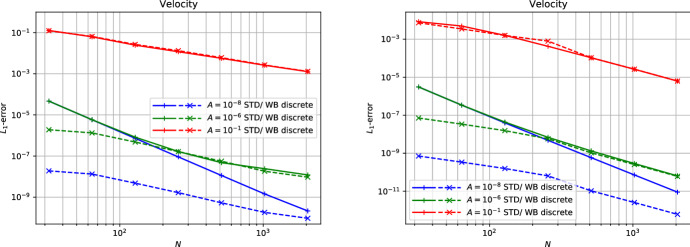
$L_1$-norm error between the numerical and reference ($N = 8192$) velocity for the isentropic (left panel) and isothermal (right panel) atmospheres. The blue, red and green line correspond to the wave amplitudes $A = 10^{-8}, 10^{-6}, 10^{-1}$, respectively. The solid and dashed lines represent the simulation performed with the standard (STD) and the well-balanced (WB) schemes, respectively

The setup is run for three amplitudes $A = 10^{-8}, 10^{-6}, 10^{-1}$ for several resolutions with the well-balanced/standard scheme. The obtained results are compared with a numerical reference solution computed with the well-balanced scheme and an $N = 8192$ resolution. The left panel of Fig. 10 displays the errors in velocity.

**Fig. 11:**
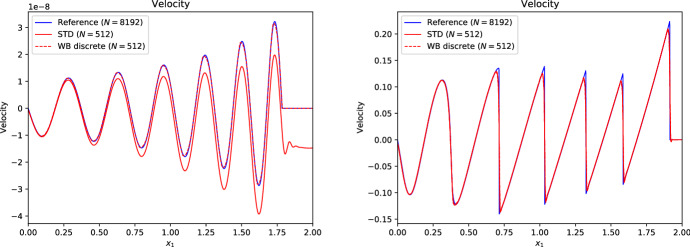
Plot of the velocity profile for the small (left) and large (right) amplitude waves propagating up the isentropic atmosphere. The solid/dashed red lines are the solutions obtained with the standard/well-balanced scheme with $N = 512$, respectively. In solid blue is also shown a reference solution obtained with the well-balanced scheme and $N = 8192$

For the small amplitude $A = 10^{-8}$ case, we observe the superiority of the well-balanced versus the standard scheme, i.e., the committed velocity errors are orders of magnitude smaller. The well-balanced scheme shows a rough second-order convergence. Although way off, the standard scheme seems to show third-order (super)convergence already observed in the well-balanced property test. The left panel of Fig. 11 shows the velocity profile for the standard and the well-balanced schemes for N = 512, together with the reference solution. The well-balanced scheme can resolve the wave pattern very accurately. On the other hand, the standard scheme shows spurious deviations because of its inability to resolve the hydrostatic background properly.

The standard and well-balanced schemes do equally well for the large amplitude $A = 10^{-1}$ case. Both show an order of convergence close to one, which is expected because the large amplitude waves quickly steepen into saw-tooth waves, propagating up the atmosphere. The velocity profile for both schemes and the reference solution are shown in the right panel of Fig. 11. This case shows that the well-balanced piecewise steady reconstruction does not destroy the shock-capturing properties of the base scheme.

The intermediate amplitude $A = 10^{-6}$ case is interesting. The well-balanced scheme is clearly superior at low resolutions ($\le 128$). The standard scheme shows superconvergence in this regime with roughly order three. This is the regime where the hydrostatic atmosphere dominates the committed error, while the wave pattern is totally unresolved. At higher resolutions ($> 128$), the wave pattern dominates the committed error, and the expected second-order accuracy is recovered. The well-balanced scheme shows an order of convergence of roughly two over the entire resolution range.

#### Wave propagation: pressure bump

The next test was introduced by LeVeque and Bale (1999). A pressure perturbation is added at the center of the atmosphere,

$$\begin{aligned} p(x) = p_{eq}(x) \left( 1 + A e^{- 200 (x - 1)^{2}} \right) , \end{aligned}$$

which excites two acoustic pulses propagating downwards/upwards. The simulations are stopped shortly before the pulses reach the domain boundaries. An advantage of this setup is that boundary conditions do not play a role, which can be delicate, especially for high-order schemes. For this test, we only show results for the isothermal atmosphere. Similar results are obtained for the isentropic atmosphere.

**Fig. 12:**
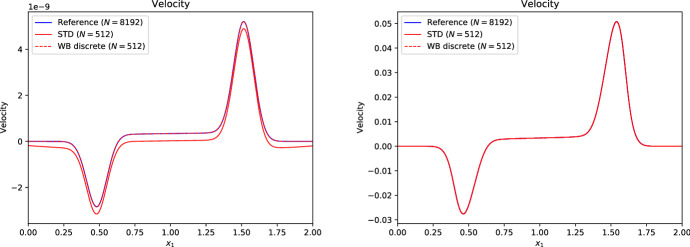
Plot of the velocity profile for the small (left) and large (right) amplitude pressure perturbations on the isothermal atmosphere. The solid/dashed red lines are the solutions obtained with the standard/well-balanced scheme with $N = 512$, respectively. In solid blue is also shown a reference solution obtained with the well-balanced scheme and $N = 8192$

The setup is also run until $t_{f} = 0.4$ for three different amplitudes $A = 10^{-8}, 10^{-6}, 10^{-1}$ and several resolution with the well-balanced/standard scheme. The results are compared to a numerically obtained reference solution computed with the well-balanced scheme at an $N = 8192$ resolution. The right panel of Fig. 10 shows the errors in velocity and Fig. 12 shows the velocity profiles for the small and large amplitudes cases. The figures show clearly that similar conclusions to the preceding test can be drawn. Hence, we do not repeat them here for brevity.

#### Polytrope

The next series of test problems model considers very simple stellar models known as polytropes. A polytrope is a static configuration of an adiabatic gaseous sphere held together by self-gravitation (see, e.g., Chandrasekhar 1967; Kippenhahn et al. 2012). These model stars are constructed from hydrostatic equilibrium

224 $$\begin{aligned} \frac{{\mathrm {d}}p}{{\mathrm {d}}r} = - \rho \frac{{\mathrm {d}}\phi }{{\mathrm {d}}r} \end{aligned}$$

and Poisson’s equation

225 $$\begin{aligned} \frac{1}{r^{2}} \frac{{\mathrm {d}}}{{\mathrm {d}}r} \left( r^{2} \frac{{\mathrm {d}}\phi }{{\mathrm {d}}r}\right) = 4 \pi G \rho \end{aligned}$$

in spherical symmetry. Here *r* is the radial coordinate and *G* is the gravitational constant.

The purely mechanical equilibrium constraints are supplemented by a thermal equilibrium in the form of a barotropic relation as

226 $$\begin{aligned} p = p(\rho ) = K \rho ^{\gamma } . \end{aligned}$$

A relation of this form is called a polytropic relation with polytropic constant *K* and polytropic exponent $\gamma$. Hence the name polytrope. Although a polytropic relation is often only an approximate model, it is an exact relation when the pressure is dominated by a (non- or relativistic) completely degenerate electron gas. For instance, the latter is the case in white dwarfs and iron cores of evolved massive stars. With the polytropic relation Eq. (226), Eqs. (224) and (225) can be combined into a single equation

227 $$\begin{aligned} \frac{1}{r^{2}} \frac{{\mathrm {d}}}{{\mathrm {d}}r}\left( r^{2} \gamma K \frac{{\mathrm {d}}\rho }{{\mathrm {d}}r} \right) = - 4 \pi G \rho , \end{aligned}$$

which is known as the Lane–Emden equation. For three values $\gamma = 6/5, 2, \infty$, the Lane–Emden equation can be solved analytically. Specifically for $\gamma = 2$, the density and pressure are given by

228 $$\begin{aligned} \rho _{eq}(r) = \rho _{C} \frac{\sin (\alpha r)}{\alpha r} \quad {\text {and}} \quad p_{eq}(r) = K \rho (r)^{2} . \end{aligned}$$

Here $\rho _{C}$ is the central density of the polytrope and

229 $$\begin{aligned} \alpha = \sqrt{\frac{4 \pi G}{2 K}} . \end{aligned}$$

The gravitational potential is given by

230 $$\begin{aligned} \phi (r) = - 2 K \rho (r) . \end{aligned}$$

Note that a $\gamma =2$ polytrope has a finite surface radius $r_{\mathrm {surface}} = \pi / \alpha$. Moreover, it fulfills the isentropic equilibrium $h(r) + \phi (r) = const = 0$ for any $r \ge 0$.

**Fig. 13:**
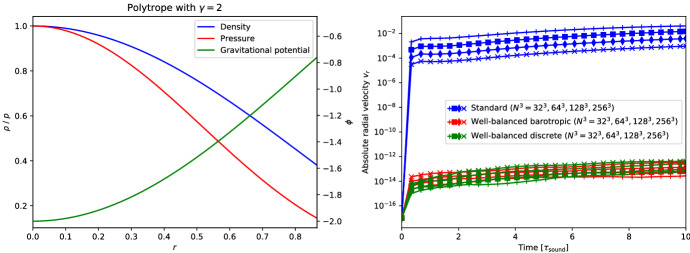
Left panel: Radial density, pressure and gravitational potential profile of the polytrope up to the corners of the three-dimensional domain ($r = \sqrt{3}/2 \approx 0.87$). Right panel: Maximum absolute radial velocity as a function of time in the simulations of the three-dimensional hydrostatic polytrope for the standard unbalanced/barotropic/discrete well-balanced second-order finite volume schemes

Following the setup of Käppeli and Mishra (2014), we set the computational domain to a three-dimensional cube $\varOmega = [-1/2, +1/2]^{3}$ uniformly discretized by $N^{3}$ cells. The model constants are set to $\rho _{C} = G = K = 1$ and we assume an ideal gas law EoS with $\gamma = 2$. The resulting radial density, pressure and gravitational potential profiles are shown in the left panel of Fig. 13. The polytrope has a sound crossing time from its center $r = 0$ to $r = 1/2$ of $\tau _{\mathrm {sound}} \approx 0.74$. Although the static configuration is built from an inherently three-dimensional physical problem, a star, it can also be applied in a Cartesian one- and two-dimensional setting as a test with non-constant gravitational acceleration (see, e.g., Li and Xing 2018b, a, 2016a; Qian et al. 2018; Grosheintz-Laval and Käppeli 2019; Berberich et al. 2021b). The simulation of a polytrope in a general relativistic context with well-balanced schemes was also considered by Gosse (2015).

We present several test cases based on the polytrope. We compare the results of a standard, unbalanced scheme with two well-balanced schemes. The first well-balanced scheme is the second-order finite volume scheme of Käppeli and Mishra (2014), also outlined in Sect. 3.3.2, based on a barotropic piecewise hydrostatic reconstruction—termed the barotropic well-balanced scheme below. The second well-balanced scheme is the second-order finite volume scheme of Käppeli and Mishra (2016), also outlined in Sect. 3.3.3, based on a discrete piecewise hydrostatic reconstruction—termed the discrete well-balanced scheme below. The standard scheme is obtained by switching off the piecewise steady reconstruction in the discrete well-balanced scheme, resulting in a second-order finite volume scheme based on piecewise linear reconstruction in primitive variables^24^.

#### Well-balanced property

The first verification is again the well-balanced property of the scheme. The three-dimensional simulation is initialized with the hydrostatic polytrope,

$$\begin{aligned} \rho (x,y,z) = \rho _{eq}(r) , \quad \varvec{v}(x,y,z) = 0 , \quad p(x,y,z) = p_{eq}(r), \quad \phi (x,y,z) = \phi (r) , \end{aligned}$$

with $r = \sqrt{x^2 + y^2 + z^2}$ using the midpoint rule to compute the cell-averaged conserved variables and is evolved for twenty sound crossing times $t_{f} = 20 ~ \tau _{\mathrm {sound}} \approx 14.8$ with the three schemes.

We remark that the initial conditions fulfill the isentropic equilibrium $h + \phi = {\text {const.}}$ exactly in a point-wise fashion. Therefore, the initial cell-averages correspond exactly to the discrete hydrostatic equilibrium preserved by the barotropic well-balanced scheme.

The errors in density and pressure at final time $t_{f}$ for the standard unbalanced/barotropic/discrete well-balanced schemes are summarized in Table 1. We first observe that the barotropic well-balanced scheme produces errors on the order of the machine precision (for double-precision) as is expected. On the other hand, the standard unbalanced scheme suffers from large spurious deviations. This corresponds to the pile-up of the truncation errors at each time step. The errors also show the expected behavior with increasing resolution for a second-order scheme. Interestingly, also the discrete well-balanced schemes shows errors on the order of the machine precision. It turns out that for the special ideal EoS with a ratio of specific heats $\gamma = 2$, the discrete well-balanced scheme preserves isentropic hydrostatic equilibrium ($h + \phi = {\text {const.}}$) exactly, even in multiple dimensions, without any requirement of alignment of grid axes and gravity force. As a result, the truncation error vanishes by design for the well-balanced schemes and only the (unavoidable) round-off errors sum up. This is further highlighted in Fig. 13, where the maximum absolute radial velocity is shown as a function of time. It is clear that the standard scheme produces spurious deviations from the hydrostatic state.

**Table 1:** $L_{1}$-error in density and pressure after twenty sound crossing times for the three-dimensional hydrostatic polytrope computed with the standard unbalanced/barotropic/discrete well-balanced second-order finite volume schemes

N	Density	Pressure
32	4.06E-02/1.47E-15/3.33E-15	4.99E-02/6.04E-16/2.82E-16
64	1.14E-02/2.70E-15/6.36E-15	1.45E-02/1.80E-16/2.50E-16
128	3.28E-03/4.83E-15/1.15E-14	4.30E-03/2.06E-16/3.05E-16
256	8.60E-04/8.52E-15/2.05E-14	1.14E-03/3.33E-16/1.18E-15

#### Wave propagation: pressure bump

To test the capability of the schemes to evolve small perturbations of the multidimensional hydrostatic equilibrium, we add a small Gaussian hump in pressure at the center of the model star

$$\begin{aligned} p(x,y,z) = p_{eq}(r) \left( 1 + A e^{-r^{2}/w^{2}} \right) , \quad \end{aligned}$$

with amplitude $A = 10^{-3}$ and width $w = 0.1$. The problem is stopped at $t_{f} = 0.2$ just before the excited waves reach the boundary. A reference solution was computed in one-dimensional spherical symmetry using the well-balanced barotropic scheme with resolution $N = 8192$.

**Fig. 14:**
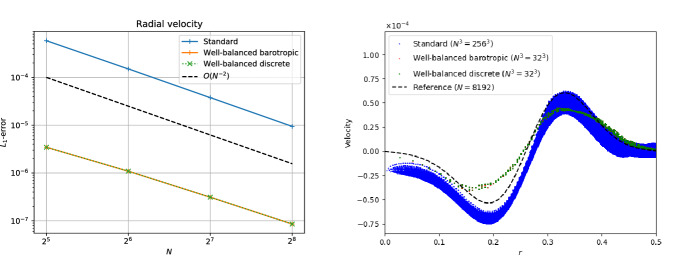
Small perturbation on a three-dimensional polytrope. Left panel: $L_{1}$-error of the radial velocity as a function resolution for the standard unbalanced, barotropic and discrete well-balanced second-order finite volume schemes. Right panel: Radial velocity scatter plot as a function of radius

The errors in radial velocity are displayed in the left panel of Fig. 14. We note that all the schemes show the expected second-order accuracy. However, we also observe that both well-balanced schemes show roughly three orders of magnitude smaller errors than the standard unbalanced scheme. Furthermore, we note that the errors for the well-balanced scheme on the coarsest resolution are smaller than the respective errors of the unbalanced scheme on the finest resolution. This fact is further highlighted in the right panel of Fig. 14 showing scatter plots of radial velocity for the standard scheme at the highest resolution ($N^{3} = 256^{3}$), both well-balanced schemes at the coarsest resolution ($N^{3} = 32^{3}$) and the reference solution. This underlines the superiority and computational efficiency of well-balanced schemes for simulating small disturbances on top of a stationary state, especially in a multi-dimensional setting. The errors in density and pressure show the same trends.

#### Wave propagation: explosion

To check the schemes’ robustness and shock-capturing properties, we add a localized overpressure region at the center of the model star

$$\begin{aligned} p(x,y,z) = \alpha (r) ~ p_{eq}(r) \quad {\text {with}} \quad \alpha (r) = {\left\{ \begin{array}{ll} 10 , &{} r \le 0.1, \\ 1 , &{} r > 0.1. \end{array}\right. } \end{aligned}$$

The triggered outward propagating explosion is evolved until $t_{f} = 0.15$. A reference solution was computed in one-dimensional spherical symmetry using the well-balanced barotropic scheme with resolution $N = 8192$.

**Fig. 15:**
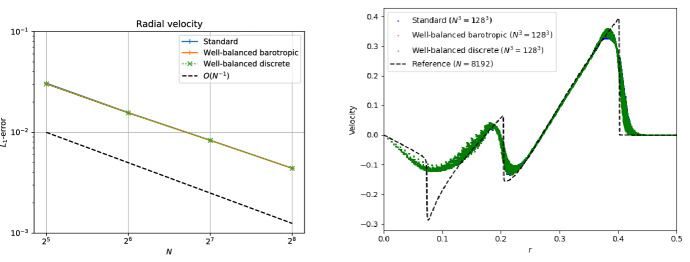
Large perturbation on a three-dimensional polytrope. Left panel: $L_{1}$-error of the radial velocity as a function resolution for the standard unbalanced, barotropic and discrete well-balanced second-order finite volume schemes. Right panel: Radial velocity scatter plot as a function of radius

The left panel of Fig. 15 displays the errors in radial velocity as a function of resolution. We observe that all schemes show comparable errors and order of convergence close to unity, as expected for discontinuous solutions. Therefore, we conclude that the piecewise steady reconstruction in both well-balanced schemes does not diminish the robustness of the standard, base shock-capturing scheme. The errors in density and pressure show similar tendencies. The right panel of Fig. 15 shows scatter plots of radial velocity as a function of radius for all the schemes, including the one-dimensional reference solution for comparison. The over-pressurized central region quickly expands driving a first strong shock wave outward. As the shock wave moves out, gravity starts to pull back some matter behind it, driving a collapse which then eventually leads to a rebound in the center. This rebound then drives another outward running shock wave. At the final time $t_{f} = 0.15$, this cycle has been repeated twice (hence the two strong shock waves) and is about to happen again as the matter is pulled back (the negative velocities below $r < 0.1$).

## Footnotes

1 Another commonly encountered regime where difficulties also arise is when the source term becomes stiff, i.e., when the timescales of the considered balance law are hugely disparate. Prominent examples are reacting flows such as in combustion and detonation phenomena. However, we will not tackle this problem in the present text.

2 In general, the fluxes and source terms may also depend explicitly on space, i.e. $\varvec{f} = \varvec{f}(\varvec{u},x)$ and $\varvec{s} = \varvec{s}(\varvec{u},x)$. For sake of notation, we suppress this dependence here.

3 Semi-discrete because only the spatial domain has been discretized. The time domain is still continuous and will be discretized in a second step.

4 In principle, the source terms depend on point values of the conserved variables, which are obtained from the reconstruction procedure, making it *r*-th order accurate. However, it could be that the source terms also involve further dependencies requiring approximations, e.g., derivatives of spatial functions, which we assume to be *s*-th order accurate.

5 Indeed, the solution may be far away from a steady state.

6 This is of course also true for the alternative piecewise steady reconstructions and source term discretizations with some additional implementation logic handling the fall back to a standard high-order finite volume scheme.

7 We stress the subtle distinction between the steady state of interest $\varvec{u}_{eq}(x)$ and its discrete approximation $\varvec{U}_{eq}(x)$ is necessary because we do not require the steady states of interest to be known analytically (see Eq. (45)). If they are, then we of course simply have $\varvec{U}_{eq}(x) \equiv \varvec{u}_{eq}(x)$.

8 Instead of the cell center, the reconstruction could be centered at the centroid of the cell.

9 Alternatively, the midpoint rule could be replaced by the centroid rule that evaluates the integrand at the centroid of the cell.

10 The weight function would correspond to the volume element of the considered curvilinear coordinates.

11 To guarantee accurate and non-oscillatory reconstruction properties, the smoothness of quantity *Q* should imply smoothness of the other quantity *W* and reciprocally.

12 In this subsection, we are explicit about the dependency of the source term on the spatial variable.

13 If the reconstruction is performed with a local characteristic variables projection, the *m*-th component of the projected flux is simply used as a smoothness measures.

14 Consider the Euler Eq. (143): the continuity equation is a conservation law, and the momentum and energy equations are balance laws due to gravitational forces.

15 If the chemical composition is important, values characterizing it need also to be given. We neglect this possibility for the sake of presentation.

16 For commonly used piecewise linear reconstructions this is obvious. For high-order (C)WENO reconstruction, see Li and Gao 2021; Don et al. 2022.

17 A polytropic relation is given by $p = K \rho ^{\gamma ^{\prime }}$, where *K* is the polytropic constant and $\gamma ^{\prime }$ the polytropic exponent (see, e.g., Chandrasekhar 1967).

18 Basically, any anchor location is fine, but, as we will see below, the cell center is a convenient choice.

19 To maintain higher-order accuracy (i.e., beyond second), the Richardson extrapolation technique can be applied to the second term on the right-hand side of Eq. (170).

20 In the sense that they don’t preserve certain steady states exactly (up to machine precision).

21 Any other anchor point is possible. However, the cell center is again a convenient choice.

22 We neglect any further dependencies such as composition.

23 Interestingly, the standard second-order base scheme shows a third-order convergence towards the steady state. Such a superconvergence has been observed before and attributed to the fact that the base scheme may be asymptotically high order, i.e., it has a higher order of accuracy at a steady state than its design order of accuracy (see Fjordholm et al. 2011).

24 Similarly, the standard scheme could be obtained by switching off the piecewise steady reconstruction in the barotropic well-balanced scheme, resulting in a second-order finite volume scheme based on piecewise linear reconstruction in conserved variables. However, both standard schemes give similar results and, therefore, only the results obtained with piecewise linear reconstruction in the primitive variables are shown below.
